# A Tutorial Review of Functional Connectivity Analysis Methods and Their Interpretational Pitfalls

**DOI:** 10.3389/fnsys.2015.00175

**Published:** 2016-01-08

**Authors:** André M. Bastos, Jan-Mathijs Schoffelen

**Affiliations:** ^1^Department of Brain and Cognitive Sciences, The Picower Institute for Learning and Memory, Massachusetts Institute of TechnologyCambridge, MA, USA; ^2^Neurobiology of Language Department, Max Planck Institute for PsycholinguisticsNijmegen, Netherlands; ^3^Donders Centre for Cognitive Neuroimaging, Donders Institute for Brain, Cognition and Behaviour, Radboud University NijmegenNijmegen, Netherlands

**Keywords:** functional connectivity (FC), coherence analysis, phase synchronization, granger causality, electrophysiology, oscillations

## Abstract

Oscillatory neuronal activity may provide a mechanism for dynamic network coordination. Rhythmic neuronal interactions can be quantified using multiple metrics, each with their own advantages and disadvantages. This tutorial will review and summarize current analysis methods used in the field of invasive and non-invasive electrophysiology to study the dynamic connections between neuronal populations. First, we review metrics for functional connectivity, including coherence, phase synchronization, phase-slope index, and Granger causality, with the specific aim to provide an intuition for how these metrics work, as well as their quantitative definition. Next, we highlight a number of interpretational caveats and common pitfalls that can arise when performing functional connectivity analysis, including the common reference problem, the signal to noise ratio problem, the volume conduction problem, the common input problem, and the sample size bias problem. These pitfalls will be illustrated by presenting a set of MATLAB-scripts, which can be executed by the reader to simulate each of these potential problems. We discuss how these issues can be addressed using current methods.

## Introduction

Different cognitive or perceptual tasks require a coordinated flow of information within networks of functionally specialized brain areas. It has been argued that neuronal oscillations provide a mechanism underlying dynamic coordination in the brain (Singer, 1999; Varela et al., 2001; Fries, 2005, 2015; Siegel et al., 2012). These oscillations likely reflect synchronized rhythmic excitability fluctuations of local neuronal ensembles (Buzsáki and Wang, 2012), and may facilitate the flow of neural information between nodes in the network when the oscillations are synchronized between those nodes (Womelsdorf et al., 2007). The neural information transmitted from one region to another is reflected by the action potentials, where the action potentials themselves may be temporally organized in bursts. These bursts may occur during oscillations and may further enhance the reliability of the information transmission (Lisman, 1997) or contribute to the establishment of long-range synchronization (Wang, 2010). The brain could dynamically coordinate the flow of information by changing the strength, pattern, or the frequency with which different brain areas engage in oscillatory synchrony.

The hypothesis that neuronal oscillations in general, and inter-areal synchronization of these oscillations in particular, are instrumental for normal brain function has resulted in widespread application of quantitative methods to evaluate neuronal synchrony in electrophysiological data. These data can be obtained with invasive or non-invasive recording techniques, and in a context that involves an experimental manipulation or in a context that is task-free. Irrespective of the recording technique and context, once the data have been collected the experimental researcher is faced with the challenge to quantify neuronal interactions as well as to provide a valid interpretation of the findings. We feel that this is challenging for several reasons. First, the methods literature provides a multitude of metrics to quantify oscillatory interactions (for example, a recent review characterized 42 distinct methods, Wang et al., 2014), often described with a large amount of technical detail. Some methods, such as coherence and Granger causality, are based on rigorous statistical theory of stochastic processes, while others, such as Phase-Locking Value (PLV) are modifications to these methods that may be somewhat *ad hoc*, but nevertheless useful. Each of these metrics has their own advantages and disadvantages, and their own vigorous adherents and opponents. It is often difficult to choose and justify which method to use, even for the technically initiated. Second, since the algorithmic implementation of a particular interaction metric can be quite complicated, some research groups may have an idiosyncratic implementation of their championed interaction measures, with limited accessibility to the wider research community. This complicates applicability of these particular metrics and the comparison to other metrics. Third, the interpretation of the findings is typically not straightforward and results are therefore prone to being over-interpreted.

The purpose of this paper is to provide a (non-exhaustive) review and tutorial for the most widely used metrics to quantify oscillatory interactions, and to provide the reader with an intuitive understanding of these metrics. First, we provide a general taxonomy of metrics to estimate functional connectivity. Next, we provide a more formal definition of the most commonly used metrics, where possible illustrating the principles behind these metrics using intuitive examples. In the last part, we discuss various analysis pitfalls that one can encounter in applying these methods to real data. Using simulations, we generate synthetic time series to demonstrate how a multitude of practical issues can arise to generate spurious functional connectivity, which should not be interpreted in terms of underlying neuronal interactions. The different problems we discuss are signal-to-noise ratio (SNR) differences, limits in sample size, volume conduction/electromagnetic field spread, the choice of reference, and the problem of unobserved inputs. The generation of the synthetic time series as well as their analysis has been performed in MATLAB, using FieldTrip (Oostenveld et al., 2011), which will enable the comparison of how different data features affect the metrics of oscillatory interactions, and how different metrics perform on the same data. For each simulation of common pitfalls, where possible we present practical steps that can be used to mitigate the concerns. These simulations are put forward as toy examples of some of the problems that can occur, and are not intended to reproduce the full complexity of real data. Yet, we hope that they will serve as a useful guide to common interpretation issues and the current state of the art in addressing them. Finally, we also discuss where future methods might be useful in order to deal with limitations of current methods.

## Taxonomy of functional connectivity metrics

**Figure 1:**
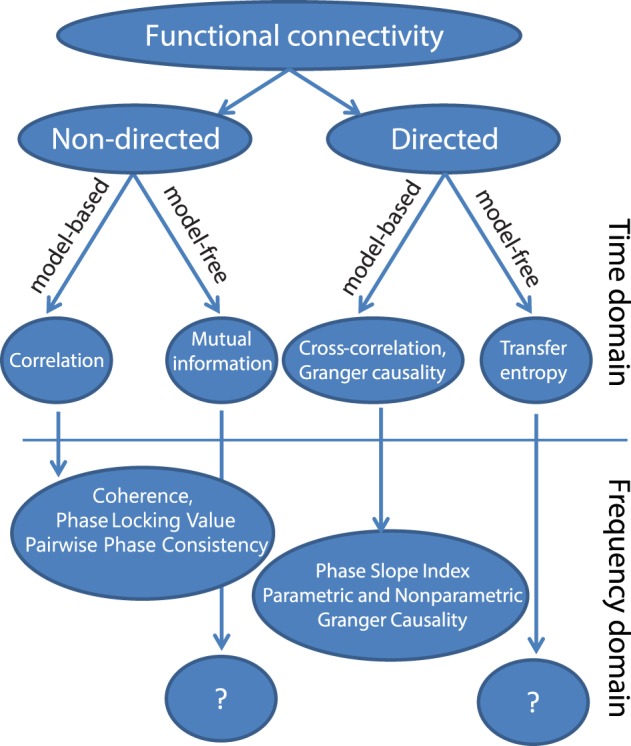
**A taxonomy of popular methods for quantifying functional connectivity**.

In this section, we will present a possible taxonomy of commonly used metrics for functional connectivity (Figure 1) and briefly describe the main motivations for each of those methods. A first subdivision that can be made is based on whether the metric quantifies the direction of the interaction. Non-directed functional connectivity metrics seek to capture some form of interdependence between signals, without reference to the direction of influence. In contrast, directed measures seek to establish a *statistical* causation from the data that is based on the maxim that causes precede their effects, and in the case of Granger causality and transfer entropy, that causes in some way predict their effects. These definitions of statistical causality were originally developed by Wiener (1956) and later practically implemented using auto-regressive models by Granger (1969). In neuroscience, a rich and growing literature has evolved that used these particular methods to quantify neuronal interactions, which has been reviewed elsewhere (Bressler and Seth, 2011; Sakkalis, 2011; Seth et al., 2015).

Within both directed and non-directed types of estimates, a distinction can be made between model-free and model-based approaches. The model-based approaches depicted in Figure 1 all make an assumption of linearity with respect to the kinds of interactions that may take place between two signals. The simplest measure for non-directed model-based interactions is the Pearson correlation coefficient, which measures the linear relationship between two random variables. In the general linear modeling framework the squared correlation coefficient (*R*^2^) represents the fraction of the variance of one of the variables (or signals) that can be explained by the other, and vice versa. A more generalized approach that does not assume a linear relationship is mutual information (Kraskov et al., 2004), which measures the generalized (linear and non-linear) interdependence between two or more variables (or time series) using concepts from information theory.

The Pearson correlation coefficient (and mutual information) as such are non-directed measures of interaction. Also, these measures ignore the temporal structure in the data, and treat the time series as realizations of random variables. In other words, this latter property means that the estimated connectivity will be the same irrespective of whether the time series have been randomly shuffled or not. Yet, when we shift the two time series with respect to one another before the correlation is computed (and do this shift at multiple lags), we will obtain the cross-correlation function, and evaluation of the cross-correlation as a function of time lag does account for temporal structure in the data. In particular, in some well-behaved cases it may sometimes be used to infer directed neuronal interactions. Specifically, the cross-correlation function has been effective to study neuronal systems containing dominant unidirectional interactions that exert their largest influence at a specific time delay (e.g., the retino-geniculate or geniculocortical feedforward pathways, Alonso et al., 1996; Usrey et al., 1998). In these cases, the time lag of maximal correlation and the magnitude of correlation can be informative about information flow between brain areas (e.g., Alonso et al., 1996). However, the interpretation of the cross-correlation function becomes complicated when it is estimated from neuronal signals with bidirectional interactions, which is the dominant interaction scenario in the majority of cortico-cortical connections. The cross-correlation functions of these interactions typically lack a clear peak, and have significant values at both positive and negative lags, indicating complex, bi-directional interactions that occur at multiple delays.

To address this limitation, other methods can be used, which assess the extent to which past values of one time series are able to predict future values of another time series, and vice versa. This notion is formally implemented in the metric of Granger causality. This metric can be computed using a linear auto-regressive model fit to the data or through non-parametric spectral matrix factorization (described in more detail later), and allows for an estimation of directed interactions. In particular, it allows for a separate estimate of interaction from signal *x* to signal *y*, and from signal *y* to signal *x*.

Finally, model-free approaches have also been developed to detect directed interactions. For instance, transfer entropy is a generalized, information-theoretic approach to study delayed (directed) interactions between time series (Schreiber, 2000; Lindner et al., 2011). Transfer entropy is a more generic implementation of the maxim that causes must precede and predict their effects, and is able to detect non-linear forms of interaction, which may remain invisible to linear approaches like Granger causality. In addition, transfer entropy has also been extended to specifically measure directed interactions between ongoing phase estimates of separate signals, which is useful to study non-linear oscillatory synchronization (Lobier et al., 2014). However, due in part to its generality, it is more difficult to interpret this measure. While the model-free approaches may be useful in quantifying non-linear neuronal interactions, this review will focus on the model-based, linear methods. Linear methods are sufficient to capture a wide-array of oscillatory interactions which are expected to take place under the hypothesis that oscillatory phase coupling governs neuronal interactions. For example, if we are interested in determining whether neuronal oscillations at similar frequencies in brain areas A and B engage in oscillatory coupling with a preferred phase difference, linear measures such as coherence or PLV will capture this interaction. If on the other hand we are interested in non-linear forms of coupling, such as cross-frequency coupling (where the phase or amplitude of frequency f1 interacts with the phase or amplitude of frequency f2, where f1 ≠ f2), then other metrics would be necessary. Therefore, the choice of method or data analysis should always be guided by the underlying hypothesis that is being tested.

A particularly important distinction if we wish to study oscillations is the distinction between metrics that are computed from the time or frequency domain representation of the signals. In order to identify individual rhythmic components that compose the measured data, and specifically to study rhythmic neuronal interactions, it is often convenient to represent the signals in the frequency domain. The transformation to the frequency domain can be achieved by the application of non-parametric (Fourier decomposition, wavelet analysis, or Hilbert transformation after bandpass filtering) or parametric techniques (autoregressive models). Subsequently, frequency-domain functional connectivity metrics can be estimated to evaluate the neuronal interactions. As we will see, many of these metrics in some way or another quantify the consistency across observations of the phase difference between the oscillatory components in the signals. A non-random distribution of phase differences could be indicative of functionally meaningful synchronization between neural populations.

We also note that there are a diverse group of methods that operate purely on the amplitude (envelope) of the oscillations to quantify amplitude and power correlations independent of phase relations. These methods have been fruitfully used to quantify large-scale brain networks by several groups (Hipp et al., 2012; Vidal et al., 2012; Foster et al., 2015). Indeed, there is now a growing debate about whether phase-relations or amplitude relations govern large-scale brain networks, with evidence existing for both perspectives (see Foster et al. this issue). In the following section we will focus on frequency-domain metrics that require an estimate of the phase of the oscillations, as opposed to metrics that quantify amplitude relations. Again, the choice of method depends on the underlying hypothesis that is being tested. Phase-based methods are appropriate for testing hypotheses where phase and moment-by-moment changes in synchronization are considered to be mechanisms for neuronal communication (e.g., Fries, 2005; Bastos et al., 2014). We will also not review methods that quantify phase-amplitude coupling as they are beyond the scope of this review and have been discussed extensively elsewhere (Canolty and Knight, 2010; Aru et al., 2015).

## Measures of synchronization

**Figure 2:**
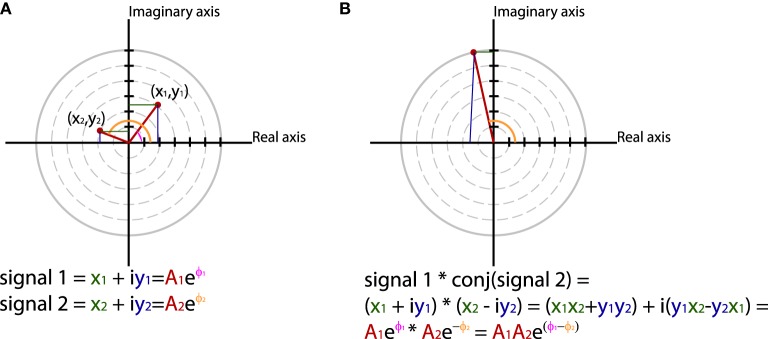
**Using polar coordinates and complex numbers to represent signals in the frequency domain**. **(A)** The phase and amplitude of two signals. **(B)** The cross-spectrum between signal 1 and 2, which corresponds to multiplying the amplitudes of the two signals and subtracting their phases.

**Figure 3:**
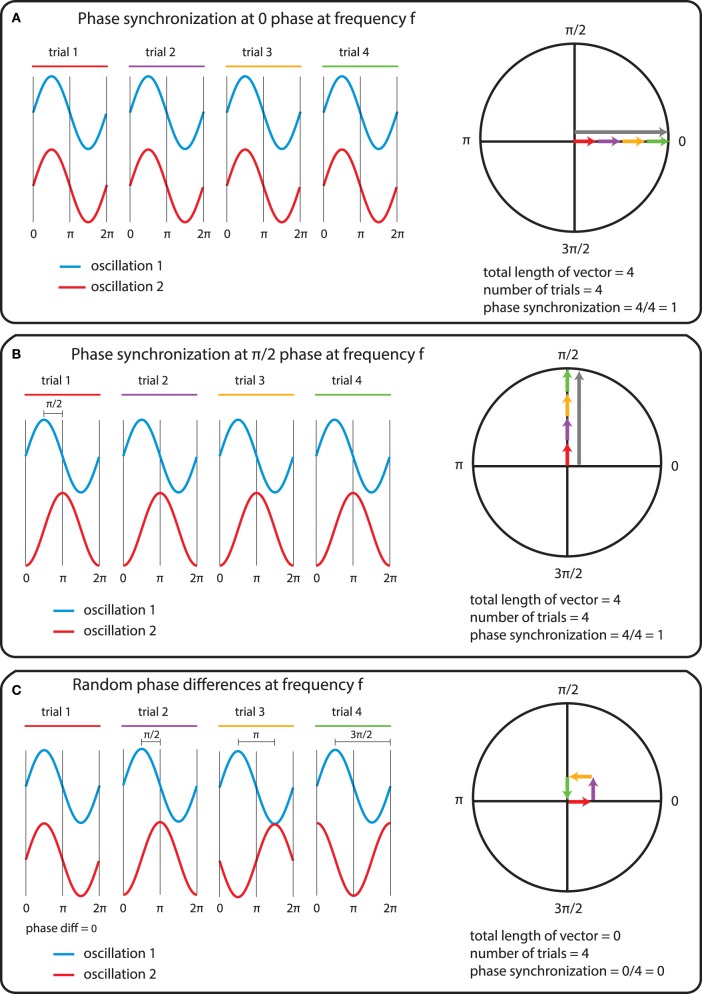
**The mechanics of the computation of phase synchrony**. **(A)** An instance of perfect phase alignment at 0 radians. **(B)** An instance of perfect synchronization at a difference of π/2 radians. **(C)** Absence of phase synchronization due to inconsistent phase differences.

In general measures of phase synchrony are computed from the frequency domain representation of a pair of signals, which represents across a set of observations (epochs or time windows), and for a set of frequency bins, an estimate of the amplitude and the phase of the oscillations. Mathematically, it is convenient to represent these amplitudes and phases combined into complex-valued numbers, *Ae*^*iφ*^, or equivalently *x* + *iy*, which can be geometrically depicted as points in a 2-dimensional Cartesian coordinate system, where the magnitude of the vector connecting the point with the origin reflects the amplitude, and the angle of the vector with the X-axis reflects the phase (see Figure 2A; equivalently the *x* and *y* coordinates of this number represent the Real and Imaginary parts, respectively). The spectral representation of individual signals is combined to obtain the cross-spectral density (the frequency domain equivalent of the cross-covariance function), by means of frequency-wise multiplication of the spectral representation of one of the signals with the complex conjugate of the spectral representation of the other signal, where complex conjugation is defined as taking the negative of the phase angle. This multiplication results in a complex number, which geometrically depicts a vector in 2-dimensional space, where the magnitude of the vector reflects the product of the two signals' amplitudes, and the angle between the vector and the X-axis reflects the two signals' difference in phase (see Figure 2B). Measures of phase synchrony now aim to capture some property of the probability distribution of the single observation cross-spectral densities, quantifying the consistency of the distribution of phase differences. One way to combine the cross-spectral densities would be to take a weighted sum, which geometrically amounts to drawing all vectors head to tail, and normalize the end result. The idea is now that if there is some consistency across observations of the phase difference between the two oscillatory signals, the length of the weighted sum will have a non-zero value (because the vectors efficiently add up), whereas it will be close to zero when the individual observations' phase differences are evenly distributed between 0 and 360°. Figure 3 displays three “toy scenarios” to illustrate this concept. Imagine two oscillators that have a consistent zero-degree phase relation over many trials or observation epochs. This is depicted graphically in the time domain in the left panels of Figure 3, showing two signals (oscillation 1 and oscillation 2) that are observed for four trials. The right panels of Figure 3 show the vector sums of the cross-spectral densities. For the time being we assumed the amplitude of the oscillations to have a value of 1. In the first scenario (Figure 3A) the phase difference is the same (and 0) for each of the observations, yielding a vector sum that has a length of 4. In the second scenario, the phase difference is also consistent across observations (i.e., 90° each time). In the third scenario however, the phase difference is not consistent across observations. In this example, the individual observations' phase differences were 0, 90, 180, and 270° respectively, resulting in individual observation cross-spectral density vectors pointing right, up, left and down. This results in a vector sum that has zero length, which coincides with the fact that there was no consistent phase difference in this case. Note that real data will fall between the two extremes of perfect phase synchronization (vector sum normalized by number of epochs equals 1) and a zero phase synchronization (vector sum to zero), even in the absence of any true phase synchronization due to sample size bias (see the section on sample size bias for an in depth discussion of this issue and how it can be mitigated).

### The coherence coefficient

One widely used metric quantifying phase synchrony between a pair of measured signals is the coherence coefficient. Mathematically, the coherence is the frequency domain equivalent to the time domain cross-correlation function. Its squared value quantifies, as a function of frequency, the amount of variance in one of the signals that can be explained by the other signal, or vice-versa, in analogy to the squared correlation coefficient in the time domain. The coherence coefficient is a normalized quantity bounded by 0 and 1, and is computed mathematically as:

(1) $$\begin{array}{l} {coh}_{xy}\left(\omega \right)=\frac{\left|\frac{1}{n}\sum_{k\;=\;1}^{n}A_{x}\left(\omega ,k\right)A_{y}\left(\omega ,k\right)e^{i\left(\varphi_{x}\left(\omega ,k\right){-\varphi }_{y}\left(\omega ,k\right)\right)}\right|}{\sqrt{\left(\frac{1}{n}\sum_{k=1}^{n}A_{x}^{2}\left(\omega ,k\right)\right)\left(\frac{1}{n}\sum_{k\;=\;1}^{n}A_{y}^{2}\left(\omega ,k\right)\right)}} \end{array}$$

The numerator term represents the length of the vector average of the individual trial cross-spectral densities between signal *x* and *y* at frequency ω. The denominator represents the square root of the product of the average of the individual trial power estimates of signals *x* and *y* at frequency ω.

It is usually more convenient to represent the averaged cross-spectral density in a single matrix, omitting the complex exponentials in the notation:

$$\begin{array}{l} S\left(\omega \right)=\left[\begin{array}{c} S_{xx}\left(\omega \right)S_{xy}\left(\omega \right)\\ S_{yx}\left(\omega \right)S_{yy}\left(\omega \right) \end{array}\right] \end{array}$$

The diagonal elements reflect the power estimates of signals *x* and *y*, and the off-diagonal elements reflect the averaged cross-spectral density terms. The coherence can then be concisely defined as:

(2) $$\begin{array}{l} {coh}_{xy}\left(\omega \right)=\frac{\left|S_{xy}\left(\omega \right)\right|}{\sqrt{S_{xx}\left(\omega \right)S_{yy}\left(\omega \right)}} \end{array}$$

### Coherency and the slope of the phase difference spectrum

When the magnitude operator (|…|) is omitted from the numerator in equation above, we obtain a complex-valued quantity called the coherency, where the phase difference angle may be interpretable (if there is a clear clustering of phase difference angles across trials) in terms of temporal delays. This is based on the notion that a consistent phase lag (or lead) across a range of frequency bins translates to a time lag (or lead) between the two time series. Note that the phase difference estimate in a single frequency bin may be ambiguous in its interpretation, because a, say, −150° phase delay cannot be disentangled from a +210° phase delay. This is due to the fact that the phase difference is circular modulo 360°. However, observing the phase difference over a range of frequencies may give an unambiguous interpretation of temporal delays. A disambiguated estimate of the time delay can be obtained when the rhythmic interaction can be described as a predominantly unidirectional time-lagged linear interaction. This estimate is then based on an estimate of the slope of the phase difference as function of the frequency range in which there is substantial coherence. This is because a fixed time delay between two time series leads to a phase difference that is a linear function of frequency. As an example, consider a time lag of 10 ms and a rhythmic process with substantial oscillatory power in the frequency range between 8 and 12 Hz. At 8 Hz, i.e., at a cycle duration of 125 ms, a time shift of 10 ms amounts 10/125 (0.08) of an oscillatory cycle (which amounts to 28.8°). At 10 Hz, the same time shift amounts to 10/100 of an oscillatory cycle (36°), and at 12 Hz, a time shift of 10 ms amounts to 10/83.33 (0.12) of an oscillatory cycle (43.2°). We would like to emphasize, however, that an interpretation of the estimated slope of the phase difference spectrum in terms of a temporal delay (and thus as an indicator of directionality) is only valid under rather ideal circumstances, where the interaction is predominantly unidirectional and well-captured under the assumption of linearity. Under non-ideal circumstances, the phase difference spectrum is a complicated function of frequency, and using it alone to assign directionality is un-principled (Witham et al., 2011; Friston et al., 2012).

### Phase slope index

Compared to the slope of the phase difference spectrum, which assumes time-lagged and linear interactions, the phase slope index (PSI) is a more generic quantity to infer dominant unidirectional interactions (Nolte et al., 2008). It is computed from the complex-valued coherency, and quantifies the consistency of the direction of the change in the phase difference across frequencies. Given a pre-specified bandwidth parameter, it computes for each frequency bin the change in the phase difference between neighboring frequency bins, weighted with the coherence. As a consequence, if for a given frequency band surrounding a frequency bin the phase difference changes consistently across frequencies, *and* there is substantial coherence, the PSI will deviate from 0. The sign of the PSI informs about which signal is temporally leading the other one. As discussed in the previous section, under situations where interactions are bi-directional, the phase difference spectrum (and consequently PSI) may fail at correctly describing the directionality. For further discussion of this, the reader is referred to Witham et al. (2011) and Vinck et al. (2015).

### Imaginary part of coherency

When the complex-valued coherency is projected onto the imaginary axis (y-axis) we obtain the imaginary part of the coherency (Nolte et al., 2004). This measure has gained some momentum over the past years, in particular in EEG/MEG connectivity studies (García Domínguez et al., 2013; Hohlefeld et al., 2013). Discarding contributions to the connectivity estimate along the real axis explicitly removes instantaneous interactions that are potentially spurious due to field spread, as discussed in depth in a later section.

### Phase locking value

When applying the formula for the computation of coherence (Equation 1) to amplitude normalized Fourier transformed signals, we get the phase locking value (PLV) (Lachaux et al., 1999):

$$\begin{array}{l} {plv}_{xy}\left(\omega \right)=\frac{\left|\frac{1}{n}\sum_{k=1}^{n}1_{x}\left(\omega ,k\right)1_{y}\left(\omega ,k\right)e^{i\left(\varphi_{x}\left(\omega ,k\right){-\varphi }_{y}\left(\omega ,k\right)\right)}\right|}{\sqrt{\left(\frac{1}{n}\sum_{k=1}^{n}1_{x}^{2}\left(\omega ,k\right)\right)\left(\frac{1}{n}\sum_{k=1}^{n}1_{y}^{2}\left(\omega ,k\right)\right)}}\\ =|\frac{1}{n}\sum_{k=1}^{n}e^{i\left(\varphi_{x}\left(\omega ,k\right){-\varphi }_{y}\left(\omega ,k\right)\right)}| \end{array}$$

As a result of these individual observation normalizations, the PLV is computed as the length of the vector-average of a set of unit-length phase difference estimates. In motivating the use of PLV, as opposed to coherence, it is often claimed that the former reflects more strictly phase synchronization than coherence, because the latter confounds the consistency of phase difference with amplitude correlation. From a mathematical point of view this may be true, but on the other hand one could argue that it is more “difficult” to obtain a meaningful non-zero coherence value in the absence of consistent phase differences, compared to when there are no amplitude correlations. Intuitively, when all individual observation cross-spectral density estimates are pointing in random directions (no phase synchrony), even in the presence of perfect amplitude correlations, expected value of their vector average will still be comparatively small. On the other hand, if all individual cross-spectral density estimates are pointing more or less into the same direction (strong phase synchrony), even in the absence of amplitude correlations, the expected value of their vector average will still be appreciable. Also, one could argue, in the case of coherence, that assigning a stronger weight to observations with a large amplitude product, one is favoring those observations that have a higher quality phase difference estimate. This realistically assumes that a higher amplitude reflects a higher SNR of the sources of interest and thus, a better quality phase estimate.

### Other measures to quantify consistent phase differences

In addition to the quantities described in the previous sections, over the past years various other metrics have been defined to quantify synchronized interactions between neuronal signals. The motivation for the development of these metrics is that most connectivity metrics suffer from interpretational difficulties. These difficulties, which are discussed and illustrated in more detail below, have prompted methods developers to define metrics that are less prone to suffer from these problems. We have already discussed the imaginary part of coherency and the phase slope index, and this section describes two additional measures that are increasingly popular, the phase lag index (PLI), and pairwise phase consistency (PPC).

The PLI is a metric that evaluates the distribution of phase differences across observations. It is computed by averaging the sign of the per observation estimated phase difference (Stam et al., 2007). It is motivated by the fact that non-zero phase differences cannot be caused by field spread (just like with the imaginary part of coherency and the PSI). More recently, some adjustments to the PLI have been proposed, yielding the weighted PLI and debiased weighted PLI to make the metric more robust against field spread, noise and sample-size bias (Vinck et al., 2011).

The PPC is a measure that quantifies the distribution of phase differences across observations (Vinck et al., 2010). Unlike the PLV, which is computed directly as a vector average of the relative phase across observations, the PPC is computed from the distribution of all pairwise differences (between pairs of observations) of the relative phases. The idea behind this is that, similar to the situation when investigating the distribution of relative phases directly, the distribution of pairwise differences in the relative phases will be more strongly clustered around an average value in the presence of phase synchronization. When no phase synchronization is present, the individual relative phase vectors are distributed around the unit circle, as are all pairwise differences of these relative phase vectors. The advantage of PPC beyond PLV is that this metric is not biased by the sample size that is used for the estimation. This means that the expected value of PPC does not change as a function of trial number (see section titled “The sample size bias problem” for more details).

In essence, many of the measures mentioned as such are not based on a principled mathematical approach (as opposed to for instance coherence and Granger causality which are rooted in the theory of stochastic processes). For instance PLI, the imaginary part of the coherency, and the phase slope index are pragmatic measures primarily put forward to address the interpretational problem of field spread, and by design intend to capture similar features of the interaction between time series. It is often an empirical question as to which of the measures is most suited to be used, and ideally one should expect that the conclusions drawn do not strongly depend on the measure that was chosen. Therefore, in general it is advisable to interrogate the data using several of these measures, in order to get a feel for how the estimates relate to one another.

### Going beyond pairwise interactions

So far we have discussed connectivity metrics that are defined between pairs of signals. Often, however, more than two signals have been recorded, and it may be relevant to investigate in more detail the pattern of multiple pairwise interactions. For this, graph theoretic approaches can be employed (Sporns, 2011). These approaches build on a valid quantification of the connectivity, are discussed extensively elsewhere and are beyond the scope of this tutorial. It is brought up here as a prelude to the next section, where we discuss the problem of common input. In general, the problem at stake is the correct inference of a direct interaction in the presence of other (possibly unobserved) sources. Although latent, unobserved sources pose a fundamental and irresolvable problem, information from sources that have been observed can be used to remove indirect influences on the estimation of connectivity. One of the ways in which this can be done in the context of coherence analysis, is by means of using the partial coherence (Rosenberg et al., 1998). If multiple signals have been recorded, one can compute for any signal pair the so-called partialized cross-spectrum, which is obtained by removing the linear contribution from all the other signals. From the partialized cross-spectrum, the partial coherence can be easily obtained. The partialized cross-spectrum can be obtained as follows. Starting from the full cross-spectral density matrix,

$$S\left(\omega \right)=\left[\begin{array}{ccccc} S_{xx}\left(\omega \right) & S_{xy}\left(\omega \right) & S_{xz_{1}}\left(\omega \right) & \ldots & S_{xz_{n}}\left(\omega \right)\\ S_{yx}\left(\omega \right) & S_{yy}\left(\omega \right) & S_{yz_{1}}\left(\omega \right) & \ldots & S_{yz_{n}}\left(\omega \right)\\ S_{z_{1}x}\left(\omega \right) & S_{z_{1}y}\left(\omega \right) & S_{z_{1}z_{1}}\left(\omega \right) & \ldots & S_{z_{1}z_{n}}\left(\omega \right)\\ \vdots & \vdots & \vdots & \ddots & \vdots \\ S_{z_{n}x}\left(\omega \right) & S_{z_{n}y}\left(\omega \right) & S_{z_{n}z_{1}}\left(\omega \right) & \ldots & S_{z_{n}z_{n}}\left(\omega \right) \end{array}\right]$$

we can partialize for the linear contributions from signals *z*_1_*-z*_*n*_ to the cross spectrum between signal *x* and *y* as follows:

$$\begin{array}{l} S_{\backslash z}\left(\omega \right)=\left[\begin{array}{cc} S_{xx}(\omega ) & S_{xy}\left(\omega \right)\\ S_{yx}(\omega ) & S_{yy}\left(\omega \right) \end{array}\right]-\left[\begin{array}{ccc} S_{xz_{1}}\left(\omega \right) & \ldots & S_{xz_{n}}\left(\omega \right)\\ S_{yz_{1}}\left(\omega \right) & \ldots & S_{yz_{n}}\left(\omega \right) \end{array}\right]\\ \;{\left[\begin{array}{ccc} S_{z_{1}z_{1}}\left(\omega \right) & \ldots & S_{z_{1}z_{n}}\left(\omega \right)\\ \vdots & \ddots & \vdots \\ S_{z_{n}z_{1}}\left(\omega \right) & \ldots & S_{z_{n}z_{n}}\left(\omega \right) \end{array}\right]}^{-1}\left[\begin{array}{cc} S_{z_{1}x}\left(\omega \right) & S_{z_{1}y}\left(\omega \right)\\ \vdots & \vdots \\ S_{z_{n}x}\left(\omega \right) & S_{z_{n}y}\left(\omega \right) \end{array}\right]\\ \;=\left[\begin{array}{cc} S_{xx\backslash z}\left(\omega \right) & S_{xy\backslash z}\left(\omega \right)\\ S_{yx\backslash z}\left(\omega \right) & S_{yy\backslash z}\left(\omega \right) \end{array}\right] \end{array}$$

### Spike-field coherence

With invasive electrophysiological signals, it is possible to record action potentials from single neurons and/or clusters of neurons (sometimes referred to as multiunit activity). In this situation, it can be informative to examine how ongoing oscillations in the simultaneously recorded LFP or EEG are related to the spikes. To this end, spikes from a given unit can be represented as a time series of ones and zeros, at the same sampling rate as the continuous LFP/EEG signals (note that it is possible to represent the spikes as a vector of spike times—this can sometimes lead to a sparser representation of the data). Once a suitable representation of the spikes is chosen, we can compute any of the above-mentioned metrics of synchronization between spikes and fields, and indeed also between different spike trains. This can often reveal significant modulation of spike timing relative to field oscillations (e.g., significant spike-field coherence), which can also be sensitive to task variables (e.g., Fries et al., 2001; Gregoriou et al., 2009). Spike-field analysis comes with its own host of caveats and challenges, which are beyond the scope of this review (interested readers can refer to Vinck et al., 2012; Ray, 2015).

## Quantification of frequency resolved, directed interactions with granger causality

So far, we have reviewed connectivity metrics that at best infer directionality of interactions based on the sign of the phase difference of the band-limited oscillatory signal components. Yet, the practical applicability of these techniques is limited to cases where there is a consistently time-lagged and predominantly uni-directional interaction. Granger causality and related metrics are capable of quantifying bi-directional interactions and provides two estimates of directed connectivity for a given signal pair, quantifying separately the directed influence of signal *x* on signal *y*, and the directed influence of signal *y* on signal *x*. Originally, the concept of Granger causality was applied to time series data in the field of economics (Granger, 1969), and extension of the concept to the frequency domain representation of time series was formulated by Geweke (1982). Although excellent introductory texts exist that explain the concept of frequency domain Granger causality and their application to neuroscience data (Ding et al., 2006), we briefly review the essential concepts in some detail in the Appendix (Supplementary Material). At this point, we restrict ourselves to the necessary essentials.

### Time domain formulation

In essence, Granger causality represents the result of a model comparison. It is rooted in the autoregressive (AR) modeling framework, where future values of time series are modeled as a weighted combination of past values of time series. Specifically, the quality of an AR-model can be quantified by the variance of the model's residuals, and Granger causality is defined as the natural logarithm of a ratio of residual variances, obtained from two different AR-models. One of these models reflect a univariate AR-model, where values of time series *x* are predicted as a weighted combination of past values of time series *x*. The other model is a bivariate AR-model, where the values of time series *x* are predicted not only based on past values of *x*, but also based on past values of another time series *y*. A substantial reduction of the variance of the residuals comparing the univariate model to the bivariate model implies that the inclusion of information about the past values of signal *y* in the prediction of signal *x* (above and beyond inclusion of only past values of signal *x*) leads to a better model for time series *x*. In these cases, the variance ratio is larger than 1, which leads to a Granger causality value that is larger than 0, signal *y* is said to Granger cause signal *x* (Granger, 1969; Ding et al., 2006; Bressler and Seth, 2011). Applying the same logic but now building autoregressive models to predict signal *y* will yield an estimate of Granger causality from signal *x* to *y*.

### Frequency domain formulation

The concept of Granger causality can also be operationalized in the frequency domain (Geweke, 1982). We refer the interested reader to the Appendix (Supplementary Material and the references mentioned therein) for more details. Here, it is sufficient to state that computation of Granger causality in the frequency domain requires the estimation of two quantities: the spectral transfer matrix (*H(*ω*)*), which is frequency dependent, and the covariance of the AR-model's residuals (Σ**)**. The following fundamental identity holds: *H*(ω)Σ*H*(ω)^*^ = *S*(ω), with *S(*ω*)* being the cross-spectral density matrix for signal pair *x, y* at frequency ω. In other words, the conjugate-symmetric cross-spectral density can be obtained by sandwiching the covariance matrix of the residuals between the spectral transfer matrix. From the cross-spectrum, the spectral transfer matrix and the residuals' covariance matrix, the frequency-dependent Granger causality can be computed as follows:

$$\begin{array}{l} {\text{GC}}_{x\to y}\left(\omega \right)=\ln\;\left(\frac{S_{yy}\left(\omega \right)}{S_{yy}\left(\omega \right)-\left(\Sigma_{xx}-\frac{\Sigma_{yx}^{2}}{\Sigma_{yy}}\right){\left|H_{yx}\left(\omega \right)\right|}^{2}}\right) \end{array}$$

### Relationship between frequency domain granger causality and coherence

Just as in the time domain formulation of Granger causality, it is possible to define a measure of total interdependence, based on the cross-spectral density estimates [more details are provided in the Appendix (Supplementary Material)]:

$$\begin{array}{l} {\text{GC}}_{x,y}\left(\omega \right)=-\ln\;\left(1-\frac{{\left|S_{xy}\left(\omega \right)\right|}^{2}}{S_{xx}\left(\omega \right)S_{yy}\left(\omega \right)}\right) \end{array}$$

The fraction between the brackets is equivalent to the squared coherence coefficient, as shown in an earlier section. In other words, there is a one-to-one relationship between the coherence coefficient and the total interdependence. In analogy to the time domain formulation, the frequency specific total interdependence can be written as a sum of three quantities: *GC*_*x, y*_(ω) = *GC*_*x*→*y*_(ω) + *GC*_*y*→*x*_(ω) + *GC*_*x*·*y*_(ω), where the instantaneous causality term is defined as:

$$\begin{array}{l} {\text{GC}}_{x\cdot y}\left(\omega \right)=\\ \ln\;\left(\frac{\left(S_{yy}\left(\omega \right)-\left(\Sigma_{xx}-\frac{\Sigma_{yx}^{2}}{\Sigma_{yy}}\right){\left|H_{yx}\left(\omega \right)\right|}^{2}\right)\left(S_{xx}\left(\omega \right)-\left(\Sigma_{yy}-\frac{\Sigma_{xy}^{2}}{\Sigma_{xx}}\right){\left|H_{xy}\left(\omega \right)\right|}^{2}\right)}{\text{det}\left(S\left(\omega \right)\right)}\right) \end{array}$$

This latter term reflects the part of the total interdependence that cannot be accounted for by time-lagged (phase-shifted) interactions between signals *x* and *y*, reflecting instantaneous common input from latent sources. Note that in the case of zero-phase lag synchronization the instantaneous causality term is not necessarily larger relative to the causal terms. Specifically, in a system with bidirectional interactions, where the magnitude and phase delay of the transfer function are approximately equal in both directions (i.e., from *x* to *y*, and from *y* to *x*), the cross-spectral density matrix can report strong synchronization at zero-phase delay that is nonetheless brought about by strong time-delayed causal interactions in both directions. Note, however, that theoretical work points out that zero-lag phase coupling is much more likely to occur in the presence of a dynamic relay through a third source, which gives common input to stabilize phase relations across the network at zero-lag (Vicente et al., 2008). Practically, this situation can be distinguished from a purely bi-directional interaction only if the two nodes with zero-lag synchronization were observed simultaneously with the third node that may (or may not) provide common inputs. More details on this scenario and how to detect it in physiological data are provided in the section “The common input problem.”

### Non-parametric vs. parametric computation of granger causality

**Figure 4:**
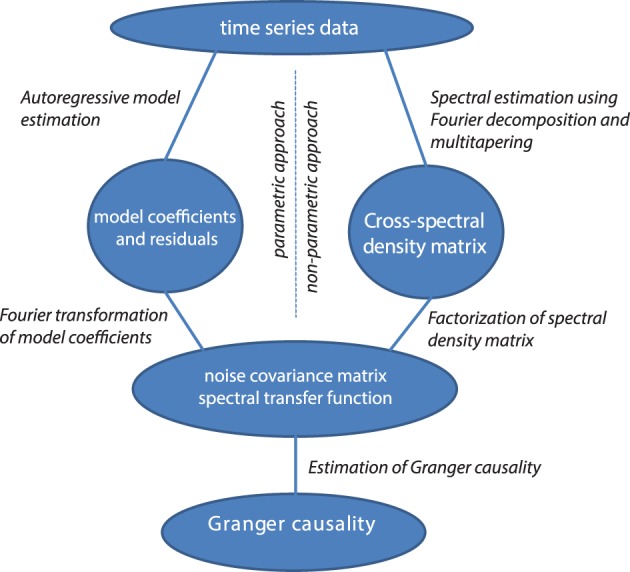
**Data processing pipeline for the computation of Granger causality, using the parametric or non-parametric approach**.

Granger causality in the frequency domain can be calculated with parametric methods (with auto-regressive models, as discussed, left half of Figure 4) or with non-parametric methods (with Fourier or wavelet-based methods). These approaches differ in how the covariance of the residuals and the transfer matrices are computed (see the right half of Figure 4). The non-parametric approach is based on the fact that the cross-spectral density matrix for a given frequency is equal to the model's residuals covariance matrix sandwiched between the transfer matrix for that frequency, as outlined above:

$$\begin{array}{l} S\left(\omega \right)=H\left(\omega \right)\Sigma H^{*}\left(\omega \right). \end{array}$$

Starting from the cross-spectral density matrix (and thus going into the opposite direction) it is possible to factorize the cross-spectral density matrix into a “noise” covariance matrix and spectral transfer matrix by applying spectral matrix factorization (Wilson, 1972)—which provides the necessary ingredients for calculating Granger causality (see Equation 4, Dhamala et al., 2008). It has been shown that parametric and non-parametric estimation of Granger causality yield very comparable results, particularly in well-behaved simulated data (Dhamala et al., 2008).

The main advantage in calculating Granger causality using this non-parametric technique is that it does not require the determination of the model order for the autoregressive model. The particular choice of the appropriate model order can be problematic, because it can vary depending on subject, experimental task, quality and complexity of the data, and model estimation technique that is used (Kaminski and Liang, 2005; Barnett and Seth, 2011). In contrast, the non-parametric estimation of Granger causality utilizes data points from the entire frequency axis, the number of which is essentially determined by the number of samples in the data window that is used for the analysis.

In comparison to the parametric estimation technique, the non-parametric spectral factorization approach requires more data and a smooth shape of the cross-spectral density (i.e., no sharp peaks as a function of frequency) to converge to a stable result. This can be seen most acutely when attempting to compute Granger causality from single trials. Although both parametric and non-parametric techniques can be used for single-trial estimates of directed connectivity, it appears that parametric estimates are more sensitive than non-parametric estimates, especially when the model order is known (Brovelli, 2012; Richter et al., 2015).

### Bivariate vs. multivariate spectral decomposition

It is relevant to note that, in multichannel recordings, the spectral transfer matrix can be obtained in two ways, irrespective of whether it is computed from a fitted autoregressive model, or through factorization of a non-parametric spectral density estimate. One can either fit a full multivariate model (or equivalently, do a multivariate spectral decomposition), where all channels are taken into account, or one can do the analysis for each channel pair separately. The latter approach typically yields more stable results (e.g., because it involves the fitting of fewer parameters), but the advantage of the former approach is that information from all channels is taken into account when estimating the interaction terms between any pair of sources. In this way, one could try and distinguish direct from indirect interactions, using an extended formulation of Granger causality, called partial Granger causality (Guo et al., 2008), or conditional Granger causality (Ding et al., 2006; Wen et al., 2013). Also, the multivariate approach yields a spectral transfer matrix that can be used to compute a set of connectivity metrics, which are related to Granger causality. These metrics are the directed transfer function (DTF) with its related metrics (Kamiñski and Blinowska, 1991), and partial directed coherence (PDC) with its related metrics (Baccalá and Sameshima, 2001). These quantities are normalized between 0 and 1, where the normalization factor is either defined as the sum along the *rows* of the spectral transfer matrix (for DTF), or as the sum along the *columns* of the inverse of the spectral transfer matrix (for PDC). By consequence of these normalizations, DTF from signal *y* to *x* reflects causal inflow from *y* to *x* as a ratio of the total inflow into signal *x*, while in its original formulation PDC from signal *y* to signal *x* reflects the causal outflow from *y* to *x* as a ratio of the total outflow from signal *y*. These measures, and their derivatives, along with motivations for preferring one over the other metric, are discussed in more detail in for example (Blinowska, 2011).

## Limitations and common problems of functional connectivity methods

The following section outlines some issues that warrant caution with respect to the interpretation of the estimated connectivity. The core issue at stake is whether the estimate of the connectivity (or the estimate of the difference in connectivity between experimental conditions) reflects a genuine effect in terms of (a change in) neuronal interactions. As we will illustrate by means of simple simulations, there are various situations that cause non-zero estimates of connectivity in the absence of true neuronal interactions. The main cause of these spurious estimates of connectivity is the fact that in MEG/EEG/LFP recordings the signals that are used for the connectivity estimate always to some extent reflect a (sometimes poorly) known mixture of a signal-of-interest (which is the activity of a neuronal population that we are interested in) and signals-of-no-interest (which we will call noise in the remainder of this paper). Another cause of spurious estimates is more related to interpretation of the observed connectivity patterns. This relates to the fact that it is impossible to state whether an observed connection is a direct connection, or whether this connection is mediated through an unobserved connection.

In what follows, we describe five common practical issues that warrant caution with respect to the interpretation of connectivity estimates. We illustrate these problems using simple simulations that are based on MATLAB-code and the FieldTrip toolbox, allowing the interested reader to get some hands-on insight into these important issues. The code has been tested on MATLAB 2013a/b and 2014a/b, using Fieldtrip version “fieldtrip-20150816.” The first three problems (the common reference problem, the volume conduction/field spread problem, and the SNR problem) are a consequence of the fact that the measured signals are always a mixture of signal-of-interest and noise, and the 4th is the problem of unobserved common input. The 5th problem is caused by unequal numbers of epochs or observation periods used to compute metrics of functional connectivity when making comparisons between conditions or across subjects.

## The common reference problem

**Figure 5:**
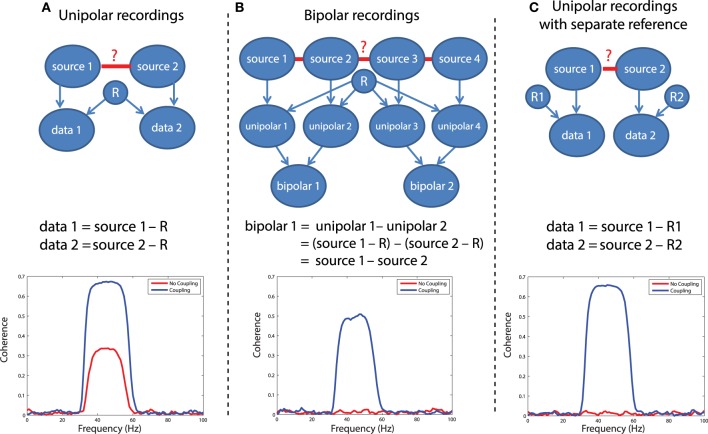
**Illustration of different referencing schemes and how each effects the calculation of coherence with and without true neuronal coupling**. **(A)** The case of unipolar recordings, which introduce spurious coherence values in the absence of coherence. **(B)** The bipolar derivation technique, which largely resolves the common reference problem. **(C)** The separate reference scheme, which also is not sensitive to common reference problems.

In LFP or EEG recordings, spurious functional connectivity estimates can result from the usage of a common reference channel. This problem is depicted graphically in Figure 5A. Imagine two recorded time series, data 1 and data 2. Each of these signals reflects the difference of the electric potential measured at the location of the electrode and at the location of the reference electrode. If the same reference electrode is used for both electrodes that are subsequently used for the connectivity estimation, the fluctuations in the electric potential at the reference location will be reflected in both time series, yielding spurious correlations at a zero time lag. Any connectivity metric that is sensitive to correlations at a zero time lag will in part be spurious. The extent to which the estimated connectivity is spurious depends on the relative strength of the potential fluctuations at the recording and reference sites. Obviously, the large majority of EEG and LFP recordings use a single reference electrode in the hardware. The following simulation illustrates the effect of a common reference electrode. We will simulate 30–60 Hz oscillatory activity in two neuronal sources, and this activity is measured by two electrodes that share a common reference electrode (which is also assumed to have an oscillatory component). We will estimate the coherence between the measured signals, in the presence and in the absence of coupling between the underlying sources.

In this simulation, we have assumed a generative model consisting of a linear mixture between various sources that each have an oscillatory component in the 30–60 Hz range. Independent sources are projected directly to the relevant data, denoted by arrows between e.g., source 1 and data 1 in Figure 5A. The common reference is treated as a shared signal that is projected with equal weight to each data channel (note the arrows between R and each of the data channels in Figure 5). Coupling between sources is simulated as a shared signal that is projected into each source in some mixture, specified in the script (*sim_commonreference.pdf*). Using this generative model, it is evident that when no real coupling is present between source 1 and source 2, there is significant artifactual coherence between data 1 and data 2, due to the common reference (Figure 5A, red trace). This is a problem, because in the absence of any underlying neuronal interaction, any connectivity estimate should be close to zero. When real coupling is introduced between source 1 and source 2, as one can see in Figure 5A, the coherence between data 1 and data 2 increases—this reflects the presence of both the common reference and real coupling. This simulation was performed assuming strong coupling between the sources. In the presence of weak coupling, the artifactual coherence may sometimes predominate—and obscure—the real coherence caused by true interactions.

One solution to the problem is to record additional channels and perform bipolar derivation to remove the common reference, and then calculate connectivity between locally-rereferenced bipolar derivations that do not share a common unipolar channel (Figure 5B). This procedure has for instance been applied in Bollimunta et al. (2009) and in Bosman et al. (2012), and relies on two assumptions: first, that the reference is equally present in the unipolar channels that will be subtracted, and second, that each of the unipolar channels reflects a different mixture of the underlying neuronal sources—otherwise, the same logic behind eliminating the common reference through bipolar derivation would also result in a cancelation of the neuronal signal. For a more rigorous discussion of the bipolar derivation procedure and its applications to neural data, see Trongnetrpunya et al. this issue. In Figure 5B each unipolar channel is depicted as reflecting the activity of an independent source, but each unipolar channel could also reflect a mixture of underlying sources, and the same logic would apply. In this scenario, the bipolar derivation removes the common reference (see the equation in Figure 5B), and the resulting bipolar signal is a subtraction of two local source activities. When coherence is now calculated directly between bipolar site 1 and bipolar site 2, it is not biased by the common reference, as shown in the lower panel of Figure 5B: when no coupling is present between the sources, coherence is close to zero (how close will depend on how many trials have gone into the analysis and the resulting bias—see section on the sample size bias), but in the presence of coupling, coherence reaches a value of 0.5.

A second possible solution to this problem is shown in Figure 5C, which is to separately reference each channel. In this case (lower panel of Figure 5C), there is again no artifactual coherence component. Therefore, while the separate referencing scheme does present a solution in principle, it may not be practical for large-scale, high density recordings. The simulations above can be realized by running the code in script *sim_commonreference.pdf*. Note that in these simulations, the reference was also assumed to have an oscillatory 30–60 Hz component—however, if the reference consisted of white noise, or a mixture of white and colored noise, and coherence were calculated between channels which both had a common reference, then the respective coherence spectrum would reflect whatever the underlying spectral shape of the reference was—due to its instantaneous mixture into both channels.

## The volume conduction/field spread problem

Another important issue in the quantification and interpretation of neuronal interactions, particularly when the estimates are based on non-invasive recordings, is caused by volume conduction. Strictly speaking, volume conduction refers to the currents flowing in the tissues surrounding active neuronal sources. Colloquially it has been adopted as a term to reflect more generally the phenomenon of the spatial spread of electromagnetic fields, which cause one recording channel or sensor to pick up the activity of multiple neuronal sources. In the case of magnetic field recordings it is sometimes more aptly referred to as field spread. At its worst, field spread can create purely artifactual coherence or phase-locking, meaning that the presence of functional connectivity between two signals would indicate not the presence of a true neuronal interaction, but instead the presence of activity from the same underlying source at the two channels. An important property of volume conduction and field spread, at least in the frequency range that is relevant for neuroscience, is that its effects are instantaneous. That is, if a dominant rhythmic neuronal source is visible at two sensors at once, the phase observed at these sensors is the same (or at a difference of 180°, when each of the sensors “sees” an opposite pole of the dipole). This property of instantaneity can be exploited to use connectivity measures that discard contributions of 0 (or 180) degrees phase differences. This will be explained in more detail below.

When considering the different electrophysiological recording techniques, field spread is considered the least problematic in invasive recordings. Spiking activity of individual neurons is by definition very focal spatially, and volume currents associated with this spiking can only be picked up at a distance of a few tens or hundreds of microns. Therefore, for the quantification of spike-spike or spike-field interactions, field (Kajikawa and Schroeder, 2011) spread is hardly an issue. In the interpretation of synchronization estimated between two LFP signals, volume conduction needs to be taken into account. The reason is that the local field potentials may reflect volume currents propagating over larger distances, exceeding 1 cm (Kajikawa and Schroeder, 2011). Field spread is by far the most problematic when performing non-invasive measurements, because of the large distance between the sensors and the neural sources, and because of the spatial blurring effect of the skull on the electric potential distribution on the scalp with EEG (Nunez et al., 1997, 1999; Srinivasan et al., 2007; Winter et al., 2007; Schoffelen and Gross, 2009). By consequence, a single underlying neuronal source will be seen at multiple EEG or MEG sensors—causing spurious correlation values between the sensors.

In the presence of field spread any given source is “visible” on multiple sensors/electrodes at once, and several strategies may be employed to alleviate the adverse effects of field spread. The first strategy attempts to “unmix” the measured signals to derive an estimate of the underlying sources. In the context of EEG/MEG recordings one should think of the application of source reconstruction algorithms (Schoffelen and Gross, 2009). In the context of LFP recordings one should think of the application of a re-referencing scheme, using for example a local bipolar derivation (as reviewed in the previous section), or estimating the current source density. The second strategy relates to the use of well-controlled experimental contrasts. The assumption here is that volume conduction affects the connectivity estimates in a similar way in both conditions, and subtraction will effectively get rid of the spurious estimates. A third strategy, as already mentioned above, would be to use connectivity metrics that capitalize on the out-of-phase interaction, discarding the interactions that are at a phase difference of 0 (or 180°). Examples of these metrics are the imaginary part of the coherency, the (weighted) phase lag index, or the phase slope index. At the expense of not being able to detect true interactions occurring at near zero-phase difference, these metrics at least provide an account of true (phase-lagged) interactions between signal components. Unfortunately, neither the application of source reconstruction techniques (which moreover add a level of complexity to the analysis of the data), nor the use of experimental contrasts or phase-lagged interaction measures will fully mitigate the effects of volume conduction. This has been described in more detail elsewhere (Schoffelen and Gross, 2009).

The script *sim_volumeconduction.pdf* illustrates some of the issues mentioned above, as well as the approaches that partially overcome them. The general approach in this set of simulations is that a set of signals is simulated at 50 “measurement locations” (MEG/EEG channels, or at “virtual electrodes”). Each of the signals consists of a mixture (due to field spread) of the activity of the underlying sources. The source activity time courses are generated by means of a generative autoregressive model. The source-to-signal mixing matrix was defined as a spatial convolution of the original source activations with a 31-point Hanning window. In other words we can consider the signals to consist of a weighted combination of the original source and the 15 most nearby sources on either side, with a spatial leakage that is dependent on the distance.

**Figure 6:**
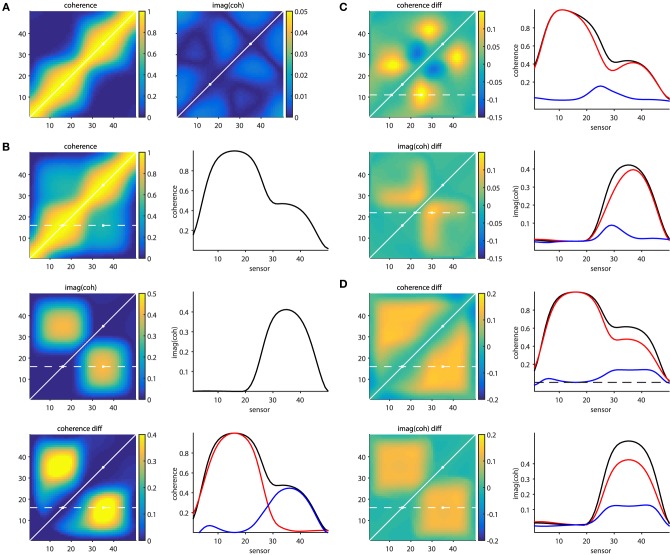
**Effects of field spread on the estimation of connectivity**. **(A)** In absence of connectivity, field spread leads to spurious coherence (left panel), while the imaginary part of coherency mitigates this effect (right panel). **(B)** In the presence of time-lagged interactions, “seed blur” caused by field spread leads to only a “shoulder” in the coherence, and not to a distant peak (top right panel). The imaginary part of coherency correctly identifies a distant peak (middle right panel). Bottom panels: the distant coherence peak is revealed when taking the difference (blue line) between the connected (black line) and unconnected (red line) situations. **(C)** Spurious differential effects show up both in the coherence (top panels) and in the imaginary part of coherency (bottom panels) when the SNR changes from one condition to another. **(D)** Spurious differential effects show up both in the coherence (top panels) and in the imaginary part of coherency (bottom panels) when the amplitude of one of the active sources changes from one condition to another.

Figure 6A shows connectivity matrices for all sensor pairs, and the source signals were constructed such that none of the underlying sources were interacting (i.e., there were no cross-terms in the autoregressive model coefficients). The noise covariance matrix had only non-zero values on the diagonal, with large values for the 16th and the 35th source. The panel on the left, displaying coherence, illustrates the seed blur as a consequence of the field spread. Each row (column) in the matrix reflects the coherence with respect to a specific seed sensor (indexed by the row number). The points closer to the diagonal are the locations that suffer most from this leakage. It can be seen that the seed blur is considerable, even if the signal is low (outside the two “active” points indicated with the white circles). Also, in the presence of a signal (i.e., when seeding from the “active” locations), the seed blur is spatially more widespread. The panel on the right shows the imaginary part of coherency, and demonstrates that (1) this quantity is low in the absence of time-lagged interactions and (2) that the seed blur is abolished. Figure 6B shows a simulation where the 16th and 35th source were actually “connected” with a non-zero time lag. The panels on the left show the coherence matrix, the imaginary part of the coherency, and the difference between the top left subpanels in b and a. The panels on the right display the connectivity in one of the rows in these matrices (indicated with the dotted white line on the left), reflecting the connectivity seeded from the 16th source. The bottom right subpanel in Figure 6B shows the coherence from the top left subpanel in Figure 6B in black, the coherence from the top left subpanel in a in red, and their difference in blue. So far, these simulations demonstrate in a very simplified scenario the effects of field spread, as well as the remedial effects provided by subtracting two conditions with different levels of connectivity between the active sources, or by focusing on the time-lagged component of the connectivity.

Real experimental data, however, is hardly ever as well-behaved as the simulated data presented above. The following provides some non-exhaustive examples of cases where the remedial strategies may yield results that can be wrongly interpreted. Figure 6C shows a situation where a true connection exists between the 16th and the 35th source, which doesn't change in strength from one condition to another. In one of the conditions an additional source is active (at location 27), which is not connected to any of the other sources. The difference maps, however, show quite some spatial structure, which is due to the differential effects exerted by the field spread on the connectivity estimates. For example, seeding from the 11th source using coherence as a connectivity metric yields a spurious non-zero difference around the 25th source. This is shown in the right panel of Figure 6C, where the individual conditions' seed-based connectivity estimates are displayed as red and black lines, and their difference is displayed in blue. Likewise, seeding from the 22nd source using the imaginary part of coherency yields a spurious non-zero difference around the 28th source. The implication of this is that differences in the activity in the contributing sources can yield spurious differences in estimated connectivity. One could argue that an appropriate seed selection would have prevented this erroneous interpretation. Had we focused on the active sources to begin with, the problem may have been less severe. Although there may be some truth in this, in practice it is often difficult to select the proper seeds a priori, for example because the relevant sources are not necessarily the ones that have the highest amplitude, and even if the seed locations are appropriately selected, spurious connectivity can still arise.

This is illustrated in Figure 6D, where two conditions are compared, with unchanging connectivity between the activated sources (16 and 35), but with a change in power for one of them. The panels on the right show the seeded connectivity (from source 16) for the condition with low (red lines) and high (black lines) power for source 35, as well as their difference (blue lines).

The difficulty of interpreting connectivity estimates due to field spread, as described above, can also be problematic for a correct inference of directed connectivity, e.g., by means of Granger causality, or related quantities. The reason for this is that the linear mixing of the source signals due to the field spread leads to signal to noise ratio differences between channels (discussed more in the next section), and inaccuracies in the estimation of the spectral transfer matrix and the noise covariance. Although some simulation work indicates that the true underlying network connectivity may be recovered by applying a renormalized version of PDC (Elsegai et al., 2015), Vinck et al. (2015) showed that instantaneous mixing is problematic for an accurate reconstruction of Granger causality. These authors propose to use a heuristic based on the investigation of the instantaneous interaction between signals in relation to their time-delayed interactions, in order to discard or accept the estimated directed connectivity to be trustworthy.

## The signal-to-noise ratio problem

Another issue that leads to interpretational problems of estimated connectivity is what we call here the SNR problem. In some way this problem is related to the field spread problem discussed above, since its underlying cause is the fact that the measured signals contain a poorly known mixture of signal-of-interest, and “noise.” Some consequences of this have already been illustrated to some extent above, where the comparison between conditions leads to spurious differences in connectivity, due to differences in SNR across conditions. Apart from posing a problem for correctly inferring a difference in connectivity across conditions, SNR differences can also be problematic when inferring differences in directional interactions between sources. It should be noted that this problem can also arise in the absence of volume conduction (e.g., when computing connectivity from locally-rereferenced LFP recordings), and therefore we believe that this problem merits a separate discussion. The core issue here is that spurious directional connectivity estimates can be obtained from two signals that have each been observed with different amounts of signal and noise. Such a situation can for instance arise when across LFP recording sites there are differences in sensor noise (e.g., due to differences in amplifier characteristics), or differences in distance to the active sources of interest, causing a difference in signal strength. While this is a problematic issue for many metrics of directed connectivity, in this section we focus specifically on how estimates of Granger causality can be corrupted by differences in SNR between signals. We illustrate the issue by presenting some simulations, which can be realized by running the code in script *sim_signaltonoise.pdf*.

**Figure 7:**
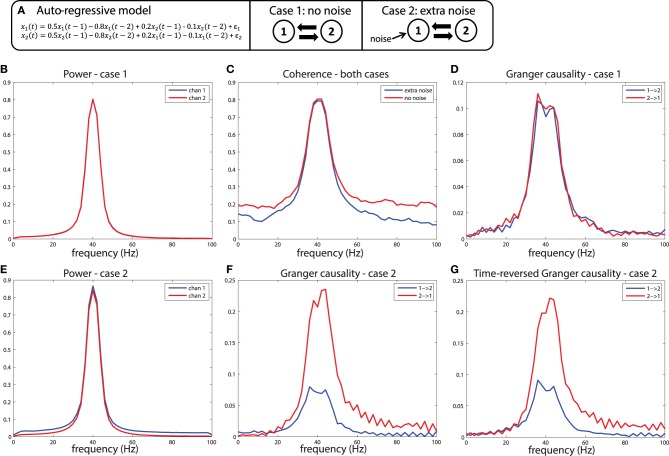
**A simulation of the signal to noise ratio problem**. **(A)** Two nodes interact bidirectionally with equal connectivity strengths in the two directions, and the data is observed without (case 1) or with (case 2) measurement noise. **(B)** Power for case 1, **(C)** Coherence for case 1 and 2, and **(D)** Granger causality estimates for case 1. **(E)** Power, **(F)** Granger causality estimates for case 2. **(G)** Granger causality estimates after time-reversing the data produced by case 2.

To understand why differences in SNR are likely to be problematic for the estimation of Granger causality, it is useful to remember that one variable has a Granger causal influence to another if the past of one time series can enhance predictions about another. In general, this approach will work to detect true connectivity, however, when applied to noisy data, this definition of causality based on prediction can lead to unexpected consequences. Imagine a situation shown in Figure 7A: variables *x*_1_and *x*_2_ are generated by an auto-regressive process that is a function of their own past at time lags 1 and 2 (the auto terms), and also a function of the past of the other variable (the cross terms), also at time lag 1 and 2. Crucially, the auto-regressive coefficients of both the auto-terms and the cross terms are identical, and the variance of the innovations (ε_1_, ε_2_) of both processes are also identical, meaning that the two variables influence each other with equal strength. Therefore, by construction, Granger causality from *x*_1_ to *x*_2_ should be identical compared to Granger causality from *x*_2_ to *x*_1_. This is indeed the case when the outputs of such a generative process are observed without measurement noise, illustrated in Figures 7B–D. In this case, as expected, both variables have nearly identical power spectra peaking at 40 Hz (Figure 7B), have a coherence spectrum that also peaks at 40 Hz (Figure 7C), and have approximately equal Granger causality at 40 Hz in both directions (Figure 7D). Note that the slight difference in Granger causality from *x*_1_ to *x*_2_ vs. *x*_2_ to *x*_1_ is due to estimation error, which approaches zero as the number of realizations of the model and subsequent observations are repeated.

Now let us consider case 2, where we observe the same system, but in the presence of noisy measurements. In this case, white noise is added to *x*_1_, but not to *x*_2_. The first result of this manipulation is that the power spectrum of *x*_1_ is shifted upward (Figure 7E), a result of adding power at all frequencies (i.e., white noise) to that channel. The coherence between the channels is also modulated by this manipulation, shown in Figure 7C, with a reduction in coherence at a broad range of frequencies when extra noise is present. When looking at the Granger causal estimates, shown in Figure 7F, we observe an asymmetric relationship, where *x*_2_ has a stronger Granger causal influence on *x*_1_ than *x*_1_ on *x*_2_. This is the consequence of the fact that the additional noise on *x*_1_ has weakened its predictive power of *x*_2_, causing an apparent asymmetry in the directionality. Note that this asymmetry is exactly in line with the definition of Granger causality based on a log ratio of prediction errors (comparing the univariate to the bivariate model), and in that sense it is not “wrong.” However, this does lead to a divergence between “Granger causality” and what we as experimentalists would like to infer as “true causality,” where we would like to infer a dominant direction of information flow from an asymmetry in Granger causal estimates.

**Figure 8:**
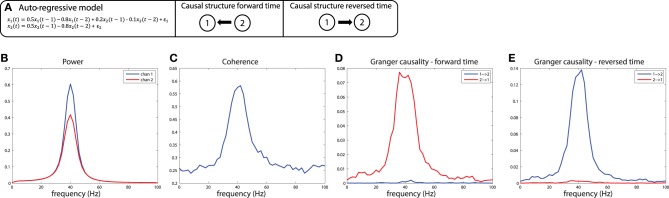
**Time reversed Granger testing reveals the presence of strong asymmetries**. **(A)** An auto-regressive model specifying a unidirectional system in which *x*_2_influences *x*_1_ at lags 1 and 2. **(B)** Power, **(C)** Coherence, and **(D)** Granger causality estimates for the forward time direction. **(E)** Granger causality estimates for the reversed time direction. Note that power and coherence remain the same when estimated from data in either the forward or reversed time directions.

These simulations depict the “worst case scenario,” when a massive difference in SNR exists between channels (SNR of *x*_1_ was 1, SNR of *x*_2_ was maximal because there was no noise added). Asymmetries in Granger causality that are driven by SNR differences have been defined by Haufe et al. as “weak asymmetries,” as opposed to “strong asymmetries” caused by true time-lagged, causal relationships (Haufe et al., 2012). In order to be able to make a distinction between weak and strong asymmetries in the interpretation of the estimated Granger causality, Haufe et al. suggest to investigate Granger causality after time-reversal of the signals. The underlying rationale is that time-reversal causes a flip in the dominant direction of interaction only in the presence of strong asymmetries, because the time-reversal operation does not affect the SNR of the individual signals (the AR coefficients of the auto-terms in the univariate AR-models are the same in the forward and backward time directions, and therefore the power spectrum and “self-prediction” is insensitive to time reversal), and thus, will not affect differences in directional interactions if these are caused by weak asymmetries. The time-reversed Granger causality computed from the data we simulated earlier is shown in Figure 7G. Evidently, time-reversal reveals that the dominant Granger causal direction (*x*_2_ to *x*_1_) remains unchanged, indicative of a weak asymmetry. On the other hand, when actual strong asymmetries underlie a directional difference in Granger causality, time-reversed testing would lead to a change in causal flow in the time-reversed compared to normal directions. In the simulations depicted in Figure 8, a strong asymmetry is created, which is a unidirectional flow from *x*_2_ to *x*_1_, leading to an asymmetric Granger causality in the appropriate direction (Figure 8D). When the signals are time-reversed, the directionality of Granger causality also flips (Figure 8E), as expected. Therefore, in practice, spurious directional differences in Granger causality that are caused only by differences in SNR may in principle be diagnosed with reversed Granger testing, which strongly reduces the false positive rate of detecting spurious Granger causality, also in the presence of linear mixing (Haufe et al., 2012; Vinck et al., 2015).

Of course, whenever possible, the SNR problem should be addressed at the experimental level, where care should be taken to minimize noise and ensure that SNR is equalized as much as possible over channels. For example, with EEG or invasive LFP recordings, impedances can be equated as much as possible prior to data recordings, and sensors should be positioned in order to maximize SNR. However, this may not be always possible in practice, for instance in non-invasive recordings (field spread), or due to intrinsic differences in activity of the underlying sources. In such cases, one could resort to stratification techniques (Bosman et al., 2012) where across conditions, the distributional properties of signal power are made as similar as possible, by means of trial subsampling. Alternatively, it has been suggested to use analytic approaches to correct for measurement noise *post-hoc*. For example, some authors have proposed algorithms to separate signal and noise components in recorded signals using state-space modeling (Nalatore et al., 2007; Sommerlade et al., 2015). This approach assumes that observed time-series represent a combination of the signals-of-interest (modeled as an auto-regressive model) and some amount of superimposed noise, and aims at separating these two quantities. In simulations with uncorrelated, Gaussian noise, this approach largely corrects the problem, yielding Granger causality estimates that are not contaminated by noise. Yet another approach is to use Dynamic Causal Models (DCM), where the generative model is an actual biophysically plausible neuronal model, rather than an autoregressive description of time series (Bastos et al., 2015a). Within the DCM framework, it is possible to make a distinction between the dynamics of the underlying biophysical process (the activation and interaction of the underlying neuronal groups) and the measurements that are made of these processes. Therefore, both correlated and uncorrelated observation noise can be taken into account, and consequently connectivity estimates are more robust to changes in SNR (Friston et al., 2014).

## The common input problem

**Figure 9:**
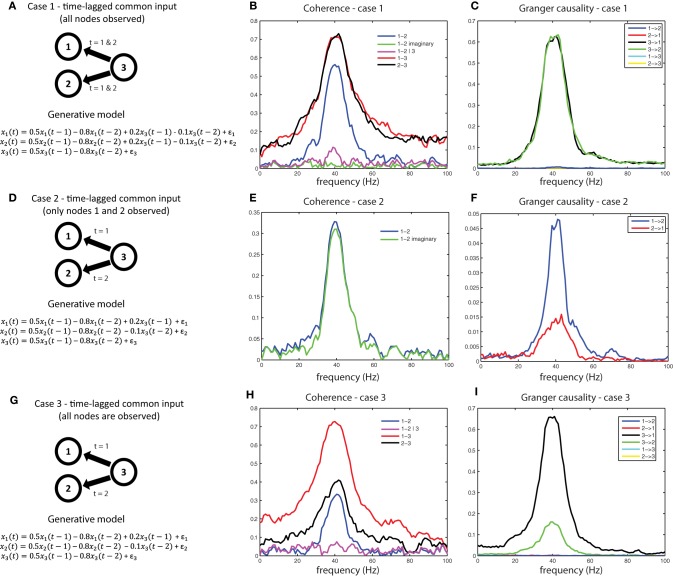
**A simulation of the common input problem**. **(A)** The auto-regressive form of a model that simulates common input from *x*_3_ to *x*_1_ and *x*_2_. **(B)** spurious coherence between *x*_1_ and *x*_2_ caused by common input. Imaginary coherence (green) and partial coherence (purple) are close to zero, indicating an interaction that is both instantaneous and mediated (by *x*_3_), respectively. **(C)** Granger causal estimates detect the common input as distinct from directed interactions between *x*_1_ and *x*_2_ (which are near zero). **(D–F)** When time-lagged common input from *x*_3_ is present and only *x*_1_ and *x*_2_ are observed, the data will appear to reflect a time-lagged interaction. This situation can only be interpreted correctly by recording the common driver **(G,H)**, and applying partial coherence **(H**) or multivariate Granger causality **(I**).

Another interpretational problem in making inferences about estimated interactions from measured data is the difficulty in distinguishing direct from indirect interactions. In other words, if a functional interaction between a pair of signals is detected, this could be caused by common input from a third source, that has not been taken into account. For example, consider the generative model shown in Figure 9A. We used a generative autoregressive model to simulate a connectivity structure between three source (*x*_1_, *x*_2_, and *x*_3_), where *x*_1_ and *x*_2_ are not directly connected, but receive common input from *x*_3_ at time lags 1 and 2. As a consequence of the common input, there will be shared variance between signals *x*_1_ and *x*_2_. Figure 9B shows the respective coherence spectra for case 1. Note the clear peak in the coherence spectrum between *x*_1_ and *x*_2_ at 40 Hz (blue trace). If we were to interpret this coherence spectrum as evidence of a direct neuronal interaction, it would be a spurious inference. Therefore, to aid in the interpretation of this coherence spectrum, we need to explicitly consider whether the shared variance that causes the observed coherence is due to common input. If we observe all three nodes, this can be done by considering coherence between *x*_1_and *x*_2_ after partializing out the contribution of *x*_3_(magenta trace in Figure 9B), which reports substantially reduced coherence values between *x*_1_and *x*_2_, indicative of shared variance (coherence) due to common input. Another metric to look at in this case would be the imaginary part of coherency (green trace), which is also close to zero, indicating that shared variance between *x*_1_ and *x*_2_ can be accounted for entirely by the zero-lag component, as could be expected by field spread or, in this case, by common input that arrives simultaneously and at the same lags at *x*_1_and *x*_2_.

We can also examine whether the shared variance between *x*_1_and *x*_2_ is due to effects of their direct interaction or common input by computing Granger causality. Figure 9C shows the Granger causality estimates between *x*_1_, *x*_2_, and *x*_3_ based on a multivariate decomposition of the spectral density matrix, i.e., observing all three nodes. By decomposing the undirected connectivity into directed connectivity using Granger causality, only connections with true underlying coupling (connections *x*_3_ 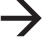
*x*_1_ and *x*_3_
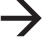
*x*_2_) survive, and the spurious connections (*x*_1_
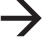
*x*_2_ and *x*_2_
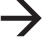
*x*_1_) are suppressed, because the shared variance is absorbed by their instantaneous interactions which are not part of a causal (lagged) interaction. This would also be a situation where conditional or partial Granger causality would be useful for diagnosing the true underlying connectivity between *x*_1_, *x*_2_, and *x*_3_. These simulations can be realized by running the code in script *sim_commoninput.pdf*.

Note that we have assumed that the common input is provided with equal coupling strength, at the same time delay, and that recordings are performed with an equal signal to noise ratio. In real data, as these assumptions are progressively violated, the estimates of Granger causality will also start to reflect spurious directional coupling. This is because changes in any of these parameters will affect how well the variance in one node can predict variance in other nodes and therefore change Granger causality values, as demonstrated in the section on signal to noise ratio. This can be especially problematic if one has not recorded from the node providing common input. This situation is simulated in the next example (case 2, Figure 9D), where *x*_3_ provides time-lagged common input by influencing *x*_1_ at lag 1 and *x*_2_ at lag 2. Coherence and Granger causality, computed after observing only *x*_1_and *x*_2_ are shown in Figures 9E,F. Coherence shows a strong coupling, which is phase-delayed due to the time lag offset in the common drive (the imaginary part of coherency, the green trace, reports almost identical values as ordinary coherence). This situation results in a significantly asymmetric Granger causality metric, with the *x*_1_ 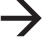
*x*_2_ influence dominant over the *x*_2_
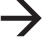
*x*_1_influence (case 3, Figure 9G). In this particular example, the only way to correctly infer the true connectivity structure would be to observe all three nodes and perform multivariate Granger causality (Figures 9G–I). Figure 9H shows that there exists strong coherence between all three nodes, even though the coherence between *x*_1_and *x*_2_ is a result of (time-lagged) common input. This can again be detected by performing partial coherence (Figure 9H, magenta trace) which removes the linear contribution of *x*_3_ onto the coherence between *x*_1_ and *x*_2_ (driving it close to zero). Furthermore, multivariate Granger causality (Figure 9I) correctly decomposes the true causal structure.

For networks with more than three nodes, there has been some progress toward inferring true network causality even in the presence of unobserved time-lagged common inputs. In particular, Elsegai and colleagues develop a technique based on renormalized PDC together with sub-network analysis, although the method can only resolve the correct connectivity in a subset of possible interactions (Elsegai et al., 2015). Therefore, common inputs can still lead to spurious inference, and currently the only guaranteed way to overcome this problem is by directly measuring nodes that could putatively provide common input.

## The sample size bias problem

Sample size bias can be a problem when connectivity measures are being compared between two or more conditions where the number of observations used to compute connectivity is different between the conditions. In general, measures of connectivity (with a few exceptions, such as PPC) are often biased quantities, where under the null hypothesis of no connectivity the estimates will deviate from zero. One of the reasons for this is that many of these metrics reflect the magnitude of a vector quantity, which always has a positive value, which is just a different way of stating that the expected value will never be zero (except in highly contrived scenarios such as Figure 3C). The amount of bias is often dependent on the sample size: the smaller the sample size, the larger the bias. The consequence of this is that, in the comparison of connectivity metrics estimated from samples with different numbers of observations, there may be a tendency to overestimate the connectivity in the condition with the smallest sample size. Also, more importantly, even in the absence of true interactions, non-zero difference may be found and reported.

**Figure 10:**
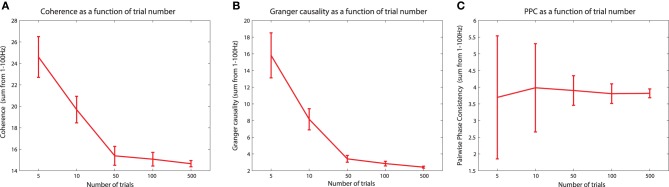
**Sample size bias for coherence and Granger causality estimates**. **(A–C)** For each respective metric, simulations based on 5, 10, 50, 100, and 500 trials were run, and coherence **(A)**, Granger causality **(B)**, and PPC **(C)** were calculated. Each panel reflects the average ± 1 standard deviation across 100 realizations.

In order to obtain estimates of phase synchronization using non-parametric spectral estimation techniques, a necessary step is to take a vector summation operation which is then normalized by the total number of trials. When fewer trials are used to estimate the phase consistency, even in the absence of phase synchrony, these vectors can all align in the same direction more easily (thereby summing to a non-zero coherence value) compared to when many trials are used. This bias is shown in Figure 10A (derived from the script *sim_samplesizebias.pdf*), where 100 simulations of a simple auto-regressive model were realized, after calculating coherence based on 5, 10, 50, 100, and 500 trials. Estimating coherence based on lower trial numbers yields an average coherence value, which is substantially higher and also more variable over different estimates compared with higher trial counts. Figure 10B shows the same simulation but using Granger causality as connectivity measure. This shows that measures like coherence, PLV, and Granger causality are positively biased when estimated using non-parametric techniques, and this fact should be taken into account for a proper inference. To estimate how much bias is present in a given estimate, one can create a reference distribution by re-arranging data segments and destroying the temporal alignment between channels. This would preserve the power spectrum of each signal but should force the cross terms of the cross spectrum to approach zero. After repeating the procedure of temporal re-alignment and connectivity estimation, one can compare the empirically observed connectivity estimate to the reference distribution and determine an appropriate level of significance. To estimate the absolute value of a connectivity metric like Granger causality, one can subtract the estimated bias to obtain a de-biased estimate (Barnett and Seth, 2014). Note that this approach is only successful in eliminating the sample-size bias associated with connectivity estimates. Other phenomena that inflate the connectivity estimates such as field spread are not properly taken into account by trial-shuffling procedures and must be dealt with in other ways.

Several approaches can be taken to account for the sample size bias. In some experimental situations, where the number of observations per condition is more or less balanced by design (but in practice turns out to be different, e.g., after artifact rejection), one strategy could be to equate the number of trials across conditions to be compared by randomly deleting observations from the condition with higher trial numbers until the trial numbers are equated. This approach is ideally embedded in a bootstrap procedure, where the random deletion of trials is repeated multiple times, to obtain a more robust estimate. In addition, for some metrics (for instance for coherence) analytic estimates of the bias can be computed and applied (Bokil et al., 2007). For coherence this usually goes hand in hand with a variance stabilizing Z-transform, which has the added advantage to facilitate a meaningful group statistical inference. Another possibility is to account for the different sample size bias within the inferential statistical framework, using a non-parametric statistical test (Maris et al., 2007). Or, in the specific case of coherence, one can consider the distributional properties and do parametric statistical inference using a jackknife procedure (Bokil et al., 2007). Alternatively, one could use a metric for detecting synchronization which is inherently unbiased, such as PPC (see Figure 10C). Finally, one could also estimate the bias associated with any particular measure of functional connectivity (for example, by shuffling the trial sequence of one channel relative to others, and calculating the metric of functional connectivity), and then subtract away this estimate of bias from the empirically observed connectivity value.

## Conclusion

In this paper, we have presented a non-exhaustive review of different methods currently used to quantify rhythmic functional connectivity. We described a taxonomy to emphasize how the methods are related to one another. For some of the most commonly used metrics, we also described the intuition behind them and some of the mathematical formulation. This was meant to demystify what data features are reflected in these connectivity metrics and how they are computed. Next we presented some important issues that may confound a correct interpretation of the connectivity, estimated from electrophysiological recordings. The description of these issues was accompanied by a series of simple simulations.

We hope that these simulations have impressed upon the reader that a naïve application of connectivity analysis to observed electrophysiological time series data can result in a myriad of spurious inferences. The simulations presented in this manuscript were meant to illustrate some of the most common cases where interpretational difficulties await the eager experimental scientist. These include problems associated with use of a common reference, the presence of volume conduction/field spread, differences in signal to noise ratio, observed or unobserved common inputs, and bias related to differences in sample size. In addition, by making available the MATLAB scripts that were used to perform the simulations, we hope that readers are encouraged run these simulations themselves, and that they will not shy away from changing the parameters in the scripts to explore in more detail the ramifications of these potential interpretational confounds. Also, the scripts can be used as a controlled environment where different solutions to these confounds can be developed and explored before they are applied on real data.

Where possible, we illustrated potential solutions to the interpretational pitfalls that we have explored. For the common reference problem, we discussed how local referencing via bipolar derivation or current source density estimation can be effective at removing common signal components. Another possible approach is to use a metric which explicitly discards the zero lag component of the interaction. Next, we discussed how the volume conduction/field spread problem can be approached in three distinct ways: by source reconstruction or local re-referencing, via the use of experimental contrasts (which makes the sometimes invalid assumption that volume conduction effects are constant between conditions), or by using methods that are insensitive to volume conduction such as the imaginary part of coherency. Field spread also affects the estimates of directed functional connectivity. Currently, there is no guaranteed method to discard volume conduction effects in this case. One way to protect against false positive inferences is to investigate the instantaneous interaction to determine whether field spread effects are too large to warrant an analysis (Vinck et al., 2015). Another possibility in principle is to include volume conduction within the estimation framework of Granger causality, which would require further development of model-based approaches to connectivity estimation.

The problem of a different signal to noise ratio across channels was discussed in the light of the potential misinterpretation of the dominant direction of interaction using directed connectivity estimates, in particular Granger causality. We illustrated a test based on time-reversed Granger causality (Haufe et al., 2012) to determine whether Granger causal asymmetries are due to strong vs. weak asymmetries in the data. In addition, we pointed out novel approaches that are based on state-space modeling and DCM, which explicitly consider the separate contribution of signal and noise to the estimated connectivity.

The problem of common input was also discussed, which is particularly relevant when researchers want to make claims with respect to whether the interaction between two neuronal groups is direct, or mediated by a third source. In general it is impossible to distinguish direct from indirect interactions if not all nodes of the functionally connected network are sampled. The problem is not a theoretical one. For instance, there is increasing evidence that some cortico-cortical interactions are mediated by the thalamus (Sherman and Guillery, 2011). Using LFP recordings from the thalamus and two cortical areas (V4 and TEO) Saalmann and colleagues showed that alpha-band cortico-cortical coherence between V4 and TEO was almost entirely eliminated after conditioning on the thalamic drive (Saalmann et al., 2012). The problem of common input can be theoretically dealt with by actually recording from the nodes that potentially provide common input, and by calculating a multivariate decomposition and conditional Granger causality or partial coherence. In practice this solution may not always be feasible due to limitations in the experimental set up. Yet, over the past years much progress has been made in increasing the coverage of neuroscience methods. For example, single cell resolution and close to full brain sampling can now be accomplished in the neonatal zebrafish, using Calcium imaging (Ahrens et al., 2013). In addition, methods that allow for significant brain coverage and dense spatial sampling of LFP and spiking activity are now becoming increasingly available in both animals (Rubehn et al., 2009; Salazar et al., 2012; Berényi et al., 2014; Schwarz et al., 2014; Lewis et al., 2015) as well as human patients undergoing neurosurgical treatments (Chang, 2015; Khodagholy et al., 2015).

Finally, we discussed the effect of sample size bias in connectivity estimates. This problem can be dealt with by equalizing sample sizes between conditions or subjects, by using statistical methods that explicitly take the sample size bias into account, or by using connectivity methods which do not suffer from sample size bias (PPC or debiased methods).

These simulations were implemented using the Fieldtrip Toolbox (Oostenveld et al., 2011). We note that other toolboxes are also available for estimation of functional connectivity data, most notably EEGLAB and the SIFT toolbox (Delorme et al., 2011), the MVGC toolbox (Barnett and Seth, 2014), the Hermes toolbox (Niso et al., 2013), the TRENTOOL toolbox (Lindner et al., 2011) for transfer entropy calculation, and the Chronux toolbox (Mitra and Bokil, 2008). Many of these tools are open source, and benefit from a wide community of users and contributors, which help to improve the tools and contribute to a larger-scale adoption.

We end by emphasizing that the difficulties we have noted do not preclude a meaningful analysis of connectivity in electrophysiological data, even when some of these issues are present. One relevant aspect that briefly needs to be mentioned is that the appropriate use of an inferential statistical procedure is a crucial step in the application of connectivity estimation techniques to neuroscience data, and in the correct interpretation of the results. Statistical tests are used to both establish significance of connectivity (against the null hypothesis of no significant coupling) as well as to establish significant changes in coupling between conditions (against the null hypothesis of no significant changes). This is typically done within a non-parametric statistical testing framework (Maris et al., 2007) and/or using bootstrap sampling and trial-reshuffling (see Methods Section in Bastos et al., 2015b). Yet, a statistical procedure used inappropriately may still lead to an over-interpretation of results (e.g., inferring significant coherence when it is actually artifactual or inferring a difference in interaction in the presence of differences in signal to noise ratio across conditions). We hope that the explicit discussion of the interpretational issues described in this review raises awareness in the wider neuroscientific community. At the same time, new methods are being continuously developed to address these problems. Overall, one can be optimistic for the future as better data, more nuanced hypotheses and experiments, and improved analysis tools continue to provide new insights into the brain's dynamic connectivity.

### Conflict of interest statement

The authors declare that the research was conducted in the absence of any commercial or financial relationships that could be construed as a potential conflict of interest. The reviewer Craig Geoffrey Richter declares that, despite having collaborated on research projects with the authors Andre M. Bastos and Jan-Mathijs Schoffelen, the review was conducted objectively and no conflict of interest exists.
