# Supplementary material for: A Tutorial Review of Functional Connectivity Analysis Methods and Their Interpretational Pitfalls
Source: Front Syst Neurosci. 2016 Jan 8;9:175. doi: 10.3389/fnsys.2015.00175 (PMC4705224; doi:10.3389/fnsys.2015.00175)
Supplement: Supplementary file 1 [file Appendix.pdf]

Appendix: Fundamentals of Granger causality and how frequency-resolved directed measures of interactions are computed.

Time domain formulation

In essence, Granger causality represents the result of a model comparison. It is defined as the natural logarithm of a variance ratio, where the individual variance terms reflect the residuals of a set of autoregressive (AR) models, fitted to the time series data. An autoregressive model describes the dynamics of a signal *x(t)* by a weighted combination of a set of temporally preceding samples *x(t-1)*, *x(t-2)*, up to *x(t-p)*, where *p* is the model order. Mathematically, a *univariate* AR-model is represented as:

$$x(t) = \sum_{\tau=1}^{p} a(\tau)x(t-\tau) + \eta(t)$$

where the index τ refers to the time lag (i.e. it refers to the τ'th preceding sample), *p* refers to the model order (the total number of preceding samples that are taken into account), the time lag dependent values of *a* are the AR-model coefficients, and the time series η is the model's residual. The variance of the residuals quantifies the quality of the model fit. The lower the variance, the better the model is capable of predicting samples of the time series based on past samples. Interestingly, electrophysiological signals can be well-approximated using AR-models, in the sense that the resulting residuals $\eta(t)$ are small relative to the original signal and the noise spectrum lacks a clear structure (i.e there are no spectral peaks, nor a 1/f shape that may be present in the original signal).
In the presence of two signals *x(t)* and *y(t)*, it is possible to fit a *bivariate* autoregressive model:

$$x(t) = \sum_{\tau=1}^{p} b(\tau)x(t-\tau) + \sum_{\tau=1}^{p} c(\tau)y(t-\tau) + \sigma(t)$$

where samples in time series *x* are now modelled as a weighted combination of temporally preceding samples of both time series *x* and *y*. Compared to the univariate autoregressive model above, this bivariate model in general yields a different set of autoregressive coefficients, as well as a different residual time series. Granger causality from signal *y* to signal *x* is now obtained as the logarithm of the variance ratio of η and σ:

$$GC_{x \to y} = \ln\left(\frac{\mathrm{var}(\eta)}{\mathrm{var}(\sigma)}\right)$$

A substantial reduction of the variance of the residuals comparing the univariate model to the bivariate model implies that the inclusion of information about the past values of signal *y* in the prediction of signal *x* (above and beyond inclusion of only past values of signal *x*) leads to a better model for time series *x*. In these cases, the variance ratio is substantially larger than 1, which leads to a Granger causality value that is larger than 0, signal *y* is said to Granger cause signal *x* (Bressler and Seth, 2011; Ding et al., 2006; Granger, 1969). Applying the same logic but now building autoregressive models to predict signal *y* will yield an estimate of Granger causality from signal *x* to *y.* Note that in cases where the bivariate model does not substantially improve the description of signal *x(t)*, $\mathrm{var}(\sigma)$, the residuals of the bivariate model will approach $\mathrm{var}(\eta)$, the residuals of the univariate model, the ratio will approach one, and Granger causality will therefore approach zero. In well-behaved cases of model estimation, the ratio will never be less than one (the bivariate model will never yield predictions that are significantly worse than the univariate model, because adding additional information to the model fit will at worst will lead to the same performance as the univariate case), and therefore Granger causality will not be negative.

There are two other quantities that are relevant to mention in the framework of Granger causality: total interdependence and instantaneous causality. To illustrate this, it is convenient to represent the bivariate autoregressive model in a single equation:

$$\begin{bmatrix} x(t) \\ y(t) \end{bmatrix} = \sum_{\tau=1}^{p} A(\tau) \begin{bmatrix} x(t-\tau) \\ y(t-\tau) \end{bmatrix} + \begin{bmatrix} \sigma_x(t) \\ \sigma_y(t) \end{bmatrix}$$

where the off-diagonal elements of the time-lag dependent matrices *A* reflect how past values of time series x predict time series y, and vice versa. We can then define a matrix Σ as the covariance matrix of the residuals:

$$\Sigma = \begin{bmatrix} \mathrm{var}(\sigma_x) & \mathrm{cov}(\sigma_x,\sigma_y) \\ \mathrm{cov}(\sigma_x,\sigma_y) & \mathrm{var}(\sigma_y) \end{bmatrix}$$

The determinant of this matrix is now defined to be $\mathrm{var}(\sigma_x)\mathrm{var}(\sigma_y) - \mathrm{cov}(\sigma_x, \sigma_y)^2$. The total interdependence is now defined as:

$$GC_{x,y} = \ln\left(\frac{var(\eta_x)var(\eta_y)}{det(\Sigma)}\right)$$

Here, the terms η_x and η_y are the residuals of the univariate autoregressive models. If time series *x* and *y* are independent, the off-diagonal model coefficients of the bivariate autoregressive model (in the matrices *A(t)*) are equal to zero, which results in (1) the variance terms of the residuals in the bivariate model to be equivalent to the variance terms in the univariate models (i.e. $var(\sigma_x) = var(\eta_x)$), and (2) the covariance term of the residuals in the bivariate model to go 0. Consequently, the total interdependence will be 0. If neither (1) nor (2) are satisfied, it will be >0.

Given the mathematical properties of logarithms of ratios and products, the following identity holds:

$$\ln\left(\frac{var(\eta_x)var(\eta_y)}{det(\Sigma)}\right) = \ln\left(\frac{var(\eta_x)}{var(\sigma_x)}\right) + \ln\left(\frac{var(\eta_y)}{var(\sigma_y)}\right) + \ln\left(\frac{var(\sigma_x)var(\sigma_y)}{det(\Sigma)}\right)$$

The item on the left hand side is the previously mentioned total interdependence. The first two elements on the right hand side of this equation represent the directional Granger causality values, and the third element is defined as the instantaneous causality ($GC_{x \cdot y}$). This latter quantity captures the part of the total interdependence that cannot be captured by time-lagged interactions of the modelled time series. In other words, the total interdependence can be thought of as a sum of three quantities, consisting of the two directed interaction terms and the instantaneous causality.

Frequency domain formulation

The mathematical details of the frequency domain formulation are clearly explained elsewhere (Ding et al., 2006), and we limit ourselves here to giving the gist of the underlying mathematics. For the frequency domain formulation of Granger causality (Geweke, 1982) it is convenient to rewrite the bivariate autoregressive model equation:

$$\begin{bmatrix} \varepsilon_x(t) \\ \varepsilon_y(t) \end{bmatrix} = \sum_{\tau=0}^{p} A(\tau) \begin{bmatrix} x(t-\tau) \\ y(t-\tau) \end{bmatrix}$$

Compared to the earlier formulation, the summation term on the right now explicitly contains time lag 0, and the corresponding coefficient matrix A at this time lag is defined to be the identity matrix (the coefficient matrices at the non-zero time lags are the negative version of the coefficient matrices in the other formulation). The summation on the right hand side can be considered to be similar to a convolution operation, where the original time series of the residuals are obtained by a convolution of the original time series with a kernel that consists of the autoregressive model coefficients. Given the fact that a convolution in the time domain is equivalent to a multiplication in the frequency domain, the Fourier transform of the above equation yields on the right hand side as a function of frequency a product of the Fourier transform of the input time series with the Fourier transform of the coefficients. The Fourier transformed version of the autoregressive model equation $\begin{bmatrix} E_x(\omega) \\ E_y(\omega) \end{bmatrix} = A(\omega) \begin{bmatrix} X(\omega) \\ Y(\omega) \end{bmatrix}$ can be further manipulated by multiplying each side of the equation by the mathematical inverse of the *A(ω)* matrix:

$$H(\omega) \begin{bmatrix} E_x(\omega) \\ E_y(\omega) \end{bmatrix} = \begin{bmatrix} X(\omega) \\ Y(\omega) \end{bmatrix}$$

where *H(ω)* (defined as $A^{-1}(\omega)$) denotes the spectral transfer matrix. Multiplying each side of this equation with its conjugate transpose, yields the following fundamental identity: $H(\omega)\Sigma H(\omega)^* = S(\omega)$, with *S(ω)* being the cross-spectral density matrix for signal pair *x,y* at frequency *ω,* and S being the covariance matrix of the residuals of the autoregressive model, where the dependence on frequency is dropped, since the residuals of the model are assumed to be spectrally white, yielding a covariance that is invariant as a function of frequency. In other words, the conjugate-symmetric cross-spectral density can be obtained by sandwiching the covariance matrix of the residuals between the spectral transfer matrix. From the cross-spectrum, the spectral transfer matrix and the residuals' covariance matrix, the frequency-dependent Granger causality can be computed as follows:

$$GC_{x \to y}(\omega) = \ln\left(\frac{S_{yy}(\omega)}{S_{yy}(\omega) - \left(\Sigma_{xx} - \frac{\Sigma_{yx}^2}{\Sigma_{yy}} \right) \left| H_{yx}(\omega) \right|^2} \right)$$

The numerator in this equation reflects the total power (at frequency *ω*) of signal *y*, and the denominator reflects the difference between the total power of signal *y* and the causal power exerted by signal *x* onto signal *y.* Stated differently, the denominator reflects the intrinsic

power of signal *x.* In this formulation, it is clear that if the causal power of *x* onto *y* is zero (*x* has not contributed any of its variance/power to *y*, which results in H_{yx} being zero, then the intrinsic power is equal to the total power, and the Granger causality from *x* to *y* is zero (Geweke, 1982). On the other hand, if there is a causal interaction from *x* to *y,* H_{yx} will be different from zero, which means that the causal power of *x* onto *y* will be larger than zero, and thus the denominator in the equation will be smaller than the numerator, yielding a GC value larger than zero (Geweke, 1982).

Relationship between frequency domain Granger causality and coherence

Just as in the time domain formulation of Granger causality, it is possible to define a measure total interdependence, based on the cross-spectral density estimates:

$$\mathrm{GC}_{\mathrm{x,y}}(\omega) = \ln\left(\frac{\mathrm{S}_{\mathrm{xx}}(\omega)\mathrm{S}_{\mathrm{yy}}(\omega)}{\det\big(\mathrm{S}(\omega)\big)}\right)$$

where the total interdependence is only equal to 0 when the off-diagonal elements (interaction terms) of the cross-spectral density are 0. If we manipulate the right hand side of the above equation by taking the negative of the logarithm while simultaneously swapping the numerator and the denominator, we get the following equation:

$$\mathrm{GC}_{\mathrm{x,y}}(\omega) = -\ln\left(\frac{\det\big(\mathrm{S}(\omega)\big)}{\mathrm{S}_{\mathrm{xx}}(\omega)\mathrm{S}_{\mathrm{yy}}(\omega)}\right)$$

writing det(S(ω)) as $S_{xx}(\omega)S_{yy}(\omega)-|S_{xy}(\omega)|^2$, the above simplifies to:

$$\mathrm{GC}_{\mathrm{x,y}}(\omega) = -\ln\left(1 - \frac{\left|\mathrm{S}_{\mathrm{xy}}(\omega)\right|^2}{\mathrm{S}_{\mathrm{xx}}(\omega)\mathrm{S}_{\mathrm{yy}}(\omega)}\right)$$

The fraction between the brackets is equivalent to the squared coherence coefficient, as shown in an earlier section (see equation 2). In other words, there is a one-to-one relationship between the coherence coefficient and the total interdependence. In analogy to the time domain formulation, the frequency specific total interdependence can be written as a sum of

three quantities: $GC_{x,y}(\omega) = GC_{x\to y}(\omega) + GC_{y\to x}(\omega) + GC_{x\cdot y}(\omega)$, where the instantaneous causality term is defined as:

$$GC_{x\cdot y}(\omega) = \ln\left(\frac{\left(S_{yy}(\omega) - (\Sigma_{xx} - \frac{\Sigma_{yx}^2}{\Sigma_{yy}})\left|H_{yx}(\omega)\right|^2\right)\left(S_{xx}(\omega) - (\Sigma_{yy} - \frac{\Sigma_{xy}^2}{\Sigma_{xx}})\left|H_{xy}(\omega)\right|^2\right)}{\det\left(S(\omega)\right)}\right)$$

This latter term reflects the part of the total interdependence that cannot be accounted for by time-lagged (phase-shifted) interactions between signals *x* and *y*, reflecting instantaneous common input from latent sources.

Non-parametric versus parametric computation of Granger causality

Granger causality in the frequency domain can be calculated with parametric methods (with auto-regressive models, as discussed, left half of Figure 4) or with non-parametric methods (with Fourier or wavelet-based methods). These approaches differ in how the covariance of the residuals and the transfer matrices are computed (see the right half of Figure 4). The non-parametric approach is based on the fact that the cross-spectral density matrix for a given frequency is equal to the model's residuals covariance matrix sandwiched between the transfer matrix for that frequency, as outlined above:

$$S(\omega) = H(\omega)\Sigma H^*(\omega)$$

Starting from the cross-spectral density matrix (and thus going into the opposite direction) it is possible to factorize the cross-spectral density matrix into a 'noise' covariance matrix and spectral transfer matrix by applying spectral matrix factorization (Wilson, 1972) – which provides the necessary ingredients for calculating Granger causality (see Equation 4, Dhamala et al., 2008). It has been shown that parametric and non-parametric estimation of Granger causality yield very comparable results, particularly in well-behaved simulated data (Dhamala et al., 2008).

The main advantage in calculating Granger causality using this non-parametric technique is that it does not require the determination of the model order for the autoregressive model. The particular choice of the appropriate model order can be problematic, because it can vary depending on subject, experimental task, quality and complexity of the data, and model

estimation technique that is used (Barnett and Seth, 2011; Kaminski and Liang, 2005). In contrast, the non-parametric estimation of Granger causality utilizes data points from the entire frequency axis, the number of which is essentially determined by the number of samples in the data window that is used for the analysis.

In comparison to the parametric estimation technique, the non-parametric spectral factorization approach requires more data and a smooth shape of the cross-spectral density (i.e. no sharp peaks as a function of frequency) to converge to a stable result. Because of this, in practice, non-parametric GC is much less robust compared to parametric techniques, although these limitations can in part be alleviated by multitaper spectral estimation (Mitra and Pesaran, 1999) and careful artifact rejection, ensuring a robust estimate and sufficient smoothness of the cross-spectral densities.

Bivariate versus multivariate spectral decomposition

It is relevant to note that, in multichannel recordings, the spectral transfer matrix can be obtained in two ways, irrespective of whether it is computed from a fitted autoregressive model, or through factorization of a non-parametric spectral density estimate. One can either fit a full multivariate model (or equivalently, do a multivariate spectral decomposition), where all channels are taken into account, or one can do the analysis for each channel pair separately. The latter approach typically yields more stable results (e.g. because it involves the fitting of fewer parameters), but the advantage of the former approach is that information from all channels is taken into account when estimating the interaction terms between any pair of sources. In this way, one could try and distinguish direct from indirect interactions, using an extended formulation of Granger causality, called partial Granger causality (Guo et al., 2008), or conditional Granger causality (Ding et al., 2006; Wen et al., 2013). Also, the multivariate approach yields a spectral transfer matrix that can be used to compute a set of connectivity metrics, which are related to Granger causality. These metrics are the directed transfer function (DTF) with its derivates (Kamiński and Blinowska, 1991), and partial directed coherence (PDC) with its derivates (Baccalá and Sameshima, 2001). These quantities are normalized between 0 and 1, where the normalization factor is either defined as the sum along the *rows* of the spectral transfer matrix (for DTF), or as the sum along the *columns* of the inverse of the spectral transfer matrix (for PDC). By consequence of these normalizations, DTF from signal y to x reflects causal inflow from y to x as a ratio of the total inflow into signal x, while in its original formulation PDC from signal y to signal x reflects the causal outflow from y to x as a ratio of the total outflow from signal y. These measures, and their derivatives, along with motivations for preferring one over the other metric, are discussed in more detail in for example Blinowska, 2011.

References

Baccalá, L. A., and Sameshima, K. (2001). Partial directed coherence: a new concept in neural structure determination. *Biol. Cybern.* 84, 463–474.

Barnett, L., and Seth, A. K. (2011). Behaviour of Granger causality under filtering: Theoretical invariance and practical application. *J. Neurosci. Methods* 201, 404–419.

Bressler, S. L., and Seth, A. K. (2011). Wiener-Granger causality: a well established methodology. *NeuroImage* 58, 323–329.

Blinowska, K. J. (2011). Review of the methods of determination of directed connectivity from multichannel data. *Med. Biol. Eng. Comput.* 49, 521–529.

Dhamala, M., Rangarajan, G., and Ding, M. (2008). Estimating Granger Causality from Fourier and Wavelet Transforms of Time Series Data. *Phys. Rev. Lett.* 100.

Ding, M., Chen, Y., and Bressler, S. L. (2006). Granger causality: basic theory and application to neuroscience. *Handb. Time Ser. Anal.*, 437–460.

Geweke, J. (1982). Measurement of Linear Dependence and Feedback between Multiple Time Series. *J. Am. Stat. Assoc.* 77, 304–313.

Granger, C. W. (1969). Investigating causal relations by econometric models and cross-spectral methods. *Econom. J. Econom. Soc.*, 424–438.

Guo, S., Seth, A. K., Kendrick, K. M., Zhou, C., and Feng, J. (2008). Partial Granger causality--eliminating exogenous inputs and latent variables. *J. Neurosci. Methods* 172, 79–93.

Kamiński, M. J., and Blinowska, K. J. (1991). A new method of the description of the information flow in the brain structures. *Biol. Cybern.* 65, 203–210.

Kaminski, M., and Liang, H. (2005). Causal influence: advances in neurosignal analysis. *Crit. Rev. Biomed. Eng.* 33, 347–430.

Mitra, P. P. & Pesaran, B. (1999). Analysis of Dynamic Brain Imaging Data. *Biophysical Journal 76,* 691–708.

Wen, X., Rangarajan, G., and Ding, M. (2013). Multivariate Granger causality: an estimation framework based on factorization of the spectral density matrix. *Philos. Transact. A Math. Phys. Eng. Sci.* 371.

Wilson, G.T. (1972). The factorization of matricial spectral densities. *Siam J. Appl. Math. 23*, 420.
