# Supplementary material for: A Tutorial Review of Functional Connectivity Analysis Methods and Their Interpretational Pitfalls
Source: Front Syst Neurosci. 2016 Jan 8;9:175. doi: 10.3389/fnsys.2015.00175 (PMC4705224; doi:10.3389/fnsys.2015.00175)
Supplement: Supplementary file 2 [file sim_commoninput.pdf]

```
%Simulate data - Node 3 gives common input to the other nodes (nodes 1 and 2) at a time delay of 1 and 2 samples

cfg             = [];
cfg.method      = 'ar';
cfg.ntrials     = 200;
cfg.triallength = 1;
cfg.fsample     = 200;
cfg.nsignal     = 3;
cfg.params(:,:,1)      = [0.55   0       0.25;
                          0      0.55    0.25;
                          0      0       0.55]; %off-diagonal entries simulate 3->1 and 3->2 influence at the first time delay
cfg.params(:,:,2)      = [-0.8  0        -0.1;
                           0   -0.8      -0.1;
                           0    0        -0.8]; %off-diagonal entries simulate 3->1 and 3->2 influence at the second time delay
cfg.noisecov     = [1 0 0;
                    0 1 0;
                    0 0 1];

data = ft_connectivitysimulation(cfg);


%%%%%%%%%%%%%%%%%%%%%%%%%%%%%%%%%%%%%%%%%%%%%%%%%%%%%%%%%%%%%%%%%%%%%%%%%%%
%Calculate power, coherence, and Granger causality based on parametric and
%non-parametric estimates as in Figure 9 b and c

%calculate the fourier coefficients (non-parametric derivation of power)
cfg        = [];
cfg.method = 'mtmfft';
cfg.taper  = 'dpss';
cfg.output = 'fourier';
cfg.tapsmofrq = 3;
cfg.foilim = [0 100];
freq       = ft_freqanalysis(cfg, data);

%freqdescriptives calculates the power spectrum
cfg         = [];
fd          = ft_freqdescriptives(cfg, freq);

%Parametric (auto-regressive model based) derivation of AR coefficients
%multivariate analysis will compute the auto-regressive coefficients and associated noise covariance matrix
```

PRO version Are you a developer? Try out the HTML to PDF API pdfcrowd.com

```
cfg           = [];
cfg.order     = 2;  %model order of 2, this is known a priori (we simulated the data using a model order of 2)
mdata         = ft_mvaranalysis(cfg, data);

%calculate cross-spectral density and transfer functions associated with the auto-regressive model
cfg        = [];
cfg.method = 'mvar';
cfg.foi    = [0:100];
mfreq      = ft_freqanalysis(cfg, mdata);

%Phase-slope index calculation
cfg           = [];
cfg.method    = 'psi';
cfg.bandwidth = 4;
psi1 = ft_connectivityanalysis(cfg, freq);

%Coherence calculation
cfg           = [];
cfg.method    = 'coh';
cfg.complex   = 'abs';
coh1 = ft_connectivityanalysis(cfg, freq);
coh2 = ft_connectivityanalysis(cfg, mfreq);

%Imaginary part of coherency
cfg           = [];
cfg.method    = 'coh';
cfg.complex   = 'imag';
icoh1 = ft_connectivityanalysis(cfg, freq);

%Partial coherence calculation
cfg           = [];
cfg.method    = 'coh';
cfg.partchannel = 'signal003';
cfg.complex   = 'abs';
pcoh1 = ft_connectivityanalysis(cfg, freq);

%Granger causality calculation
cfg           = [];
cfg.method    = 'granger';
cfg.granger.sfmethod = 'multivariate';
g1 = ft_connectivityanalysis(cfg, freq);
```

PRO version Are you a developer? Try out the HTML to PDF API pdfcrowd.com

```
g1 = ft_checkdata(g1, 'cmbrepresentation', 'full');
g2 = ft_connectivityanalysis(cfg, mfreq);


%%%%%%%%%%%%%%%%%%%%%%%%%%%%%%%%%%%%%%%%%%%%%%%%%%%%%%%%%%%%%%%%%%%%%%%%%%%%%%%
%now plot the output for the various connectivity measures as in Figure 9 b and c
%output variables 1 - nonparam 2 - param

figure;plot(fd.freq, fd.powspctrm(1,:)); hold on
plot(fd.freq, fd.powspctrm(2,:),'r');
plot(fd.freq, fd.powspctrm(3,:),'k');
legend('Power ch 1', 'Power ch 2','Power ch 3');
title('Nonparametric Power');

figure;plot(mfreq.freq, squeeze(abs(mfreq.crsspctrm(1,1,:)))); hold on;
plot(mfreq.freq, squeeze(abs(mfreq.crsspctrm(2,2,:))),'r');
plot(mfreq.freq, squeeze(abs(mfreq.crsspctrm(3,3,:))),'k');
legend('Power ch 1', 'Power ch 2','Power ch 3');
title('Parametric Power');

figure;plot(g1.freq,squeeze(coh1.cohspctrm(1,2,:))); hold on;
plot(g1.freq,squeeze(coh1.cohspctrm(1,3,:)),'r');
plot(g1.freq,squeeze(coh1.cohspctrm(2,3,:)),'k');
plot(g1.freq,squeeze(abs(icoh1.cohspctrm(1,2,:))),'g');
plot(g1.freq,squeeze(pcoh1.cohspctrm(1,2,:)),'m');
legend('1-2','1-3','2-3', '1-2 imaginary', '1-2 | 3');
title('Nonparametric Coherence spectrum');

figure;plot(g1.freq,squeeze(coh2.cohspctrm(1,2,:))); hold on
plot(g1.freq,squeeze(coh2.cohspctrm(1,3,:)),'r');
plot(g1.freq,squeeze(coh2.cohspctrm(2,3,:)),'k');
legend('1-2','1-3','2-3');
title('Parametric Coherence spectrum');

figure;plot(g1.freq,squeeze(psi1.psispctrm(1,2,:))); hold on;
plot(g1.freq,squeeze(psi1.psispctrm(1,3,:)),'r');
plot(g1.freq,squeeze(psi1.psispctrm(2,3,:)),'k');
legend('1->2','1->3','3->2');title('PSI nonparametric');

figure;plot(g1.freq,squeeze(g1.grangerspctrm(1,2,:)));hold on
plot(g1.freq,squeeze(g1.grangerspctrm(2,1,:)),'r');
plot(g1.freq,squeeze(g1.grangerspctrm(3,1,:)),'k');
```

PRO version Are you a developer? Try out the HTML to PDF API pdfcrowd.com

```
plot(g1.freq,squeeze(g1.grangerspctrm(3,2,:)),'g');
plot(g1.freq,squeeze(g1.grangerspctrm(1,3,:)),'c');
plot(g1.freq,squeeze(g1.grangerspctrm(2,3,:)),'y');
title('Granger nonparametric estimates');legend('1->2','2->1','3->1','3->2','1->3','2->3')

figure; plot(g1.freq,squeeze(g2.grangerspctrm(1,2,:)));hold on;
plot(g1.freq,squeeze(g2.grangerspctrm(2,1,:)),'r');
plot(g1.freq,squeeze(g2.grangerspctrm(3,1,:)),'k');
plot(g1.freq,squeeze(g2.grangerspctrm(3,2,:)),'g');
plot(g1.freq,squeeze(g2.grangerspctrm(1,3,:)),'c');
plot(g1.freq,squeeze(g2.grangerspctrm(2,3,:)),'y');
legend('1->2','2->1','3->1','3->2','1->3','2->3');title('Granger parametric estimates ');


%%%%%%%%%%%%%%%%%%%%%%%%%%%%%%%%%%%%%%%%%%%%%%%%%%%%%%%%%%%%%%%%%%%%%%%%%%%
%Simulate the case time-lagged common input (Figure 9 D-I)
%Node 3 gives common input to the other nodes (nodes 1 and 2) at a time delay of 1 and 2 samples
cfg             = [];
cfg.method      = 'ar';
cfg.ntrials     = 500;
cfg.triallength = 1;
cfg.fsample     = 200;
cfg.nsignal     = 3;
%Auto-regressive coefficients at time lag 1
cfg.params(:,:,1)      = [0.55   0       0.25;
                          0      0.55    0;
                          0      0       0.55]; %off-diagonal entry simulates 3->1 at the first time delay
%Auto-regressive coefficients at time lag 2
cfg.params(:,:,2)      = [-0.8  0         0;
                           0   -0.8      -0.1;
                           0    0        -0.8];  %off-diagonal simulates 3->2 at the second time delay
cfg.noisecov     = [1 0 0;
                    0 1 0;
                    0 0 1];
cfg.absnoise = 0;

data = ft_connectivitysimulation(cfg);

%Remove the third node from the data to simulate a situation where we do
%not observe the source of common input (Figure 9 D-F)
cfg         = [];
cfg.channel = data.label(1:2);
```
PRO version Are you a developer? Try out the HTML to PDF API pdfcrowd.com

```
data        = ft_selectdata(cfg, data);

%%%%%%%%%%%%%%%%%%%%%%%%%%%%%%%%%%%%%%%%%%%%%%%%%%%%%%%%%%%%%%%%%%%%%%%%%%%
%Calculate power, coherence, and Granger causality based on parametric and
%non-parametric estimates

%calculate the fourier coefficients (non-parametric derivation of power)
cfg        = [];
cfg.method = 'mtmfft';
cfg.taper  = 'dpss';
cfg.output = 'fourier';
cfg.tapsmofrq = 3;
cfg.foilim = [0 100];
freq       = ft_freqanalysis(cfg, data);

%freqdescriptives calculates the power spectrum
fd         = ft_freqdescriptives([], freq);

%Phase-slope index calculation
cfg           = [];
cfg.method    = 'psi';
cfg.bandwidth = 4;
psi1 = ft_connectivityanalysis(cfg, freq);

%Coherence calculation
cfg           = [];
cfg.method    = 'coh';
cfg.complex   = 'abs';
coh1 = ft_connectivityanalysis(cfg, freq);

%Imaginary part of coherency
cfg           = [];
cfg.method    = 'coh';
cfg.complex   =  'imag';
icoh1 = ft_connectivityanalysis(cfg, freq);

%Granger causality calculation (bivariate)
cfg           = [];
cfg.method    = 'granger';
cfg.sfmethod  = 'bivariate';
g1 = ft_connectivityanalysis(cfg, freq);
g1 = ft_checkdata(g1, 'cmbrepresentation', 'full');
```

PRO version Are you a developer? Try out the HTML to PDF API pdfcrowd.com

```
%%%%%%%%%%%%%%%%%%%%%%%%%%%%%%%%%%%%%%%%%%%%%%%%%%%%%%%%%%%%%%%%%%%%%%%%%%%%
%Plot the output for the various connectivity measures:

figure;plot(fd.freq, fd.powspctrm(1,:)); hold on
plot(fd.freq, fd.powspctrm(2,:),'r');
legend('Power ch 1', 'Power ch 2');
title('Nonparametric Power');

figure;plot(g1.freq,squeeze(coh1.cohspctrm(1,2,:))); hold on;
plot(g1.freq,squeeze(abs(icoh1.cohspctrm(1,2,:))), 'g');
legend('1-2', '1-2 imaginary');
title('Nonparametric Coherence spectrum');

figure;plot(g1.freq,squeeze(psi1.psispctrm(1,2,:))); hold on;
title('PSI nonparametric');

figure;plot(g1.freq,squeeze(g1.grangerspctrm(1,2,:)));hold on
plot(g1.freq,squeeze(g1.grangerspctrm(2,1,:)),'r');
title('Granger nonparametric estimates ');legend('1->2','2->1')

%%%%%%%%%%%%%%%%%%%%%%%%%%%%%%%%%%%%%%%%%%%%%%%%%%%%%%%%%%%%%%%%%%%%%%%%%%%%
%Re-run the simulation, this time observing all three nodes as in Figure 9 G-I

cfg             = [];
cfg.method      = 'ar';
cfg.ntrials     = 200;
cfg.triallength = 1;
cfg.fsample     = 200;
cfg.nsignal     = 3;
cfg.params(:,:,1)      = [0.55   0       0.25;
                          0      0.55    0;
                          0      0       0.55]; %this simulates 3->1 at the first time delay
cfg.params(:,:,2)      = [-0.8  0        0;
                           0   -0.8      -0.1;
                           0    0        -0.8];  %this simulates 3->2 at the second time delay
cfg.noisecov     = [1 0 0;
                    0 1 0;
                    0 0 1];

data = ft_connectivitysimulation(cfg);
```

PRO version Are you a developer? Try out the HTML to PDF API pdfcrowd.com

```
%%%%%%%%%%%%%%%%%%%%%%%%%%%%%%%%%%%%%%%%%%%%%%%%%%%%%%%%%%%%%%%%%%%%%%%%%%%%%%%%
%Calculate power, coherence, and Granger causality based on non-parametric estimates

%calculate the fourier coefficients (non-parametric derivation of power)
cfg        = [];
cfg.method = 'mtmfft';
cfg.taper  = 'dpss';
cfg.output = 'fourier';
cfg.tapsmofrq = 3;
cfg.foilim = [0 100];
freq       = ft_freqanalysis(cfg, data);

%freqdescriptives calculates the power spectrum
cfg         = [];
cfg.complex = 'complex';
cfg.jackknife     = 'yes';

fd          = ft_freqdescriptives(cfg, freq);

ntrl          = length(data.trial);
nsmp          = size(data.trial{1},2);
data.cfg.trl  = [1:nsmp:(ntrl-1)*nsmp+1;nsmp:nsmp:ntrl*nsmp]';
data.cfg.trl(:,3) = 0;
cfg           = [];
cfg.t_ftimwin = 1;
cfg.toi       = 0.5;
cfg.order     = 2;                          %model order of 2, this is known a priori (we simulated the data using a model order of 2)
mdata         = ft_mvaranalysis(cfg, data);

%calculate cross-spectral density and transfer functions associated with the auto-regressive model
cfg        = [];
cfg.method = 'mvar';
cfg.foi    = [0:100];
mfreq      = ft_freqanalysis(cfg, mdata);

%Phase-slope index calculation
cfg           = [];
cfg.method    = 'psi';
cfg.bandwidth = 4;
psi1 = ft_connectivityanalysis(cfg, freq);

%Coherence calculation
```

PRO version Are you a developer? Try out the HTML to PDF API pdfcrowd.com

```
cfg           = [];
cfg.method    = 'coh';
cfg.complex   = 'abs';
coh1 = ft_connectivityanalysis(cfg, freq);
coh2 = ft_connectivityanalysis(cfg, mfreq);


%Partial coherence calculation
cfg           = [];
cfg.method    = 'coh';
cfg.partchannel = 'signal003';
cfg.complex   = 'abs';
pcoh1 = ft_connectivityanalysis(cfg, freq);


%Granger causality calculation
cfg           = [];
cfg.method    = 'granger';
cfg.granger.sfmethod = 'multivariate';
g1 = ft_connectivityanalysis(cfg, freq);
g1 = ft_checkdata(g1, 'cmbrepresentation', 'full');
g2 = ft_connectivityanalysis(cfg, mfreq);

%%%%%%%%%%%%%%%%%%%%%%%%%%%%%%%%%%%%%%%%%%%%%%%%%%%%%%%%%%%%%%%%%%%%%%%%%%%%
%now plot the output for the various connectivity measures:
%out put variables 1 - nonparam 2 - param

figure;plot(fd.freq, fd.powspctrm(1,:)); hold on
plot(fd.freq, fd.powspctrm(2,:),'r');
plot(fd.freq, fd.powspctrm(3,:),'k');
legend('Power ch 1', 'Power ch 2','Power ch 3');
title('Nonparametric Power');

figure;plot(mfreq.freq, squeeze(abs(mfreq.crsspctrm(1,1,:)))); hold on;
plot(mfreq.freq, squeeze(abs(mfreq.crsspctrm(2,2,:))),'r');
plot(mfreq.freq, squeeze(abs(mfreq.crsspctrm(3,3,:))),'k');
legend('Power ch 1', 'Power ch 2','Power ch 3');
title('Parametric Power');


figure;plot(g1.freq,squeeze(coh1.cohspctrm(1,2,:))); hold on;
plot(g1.freq,squeeze(coh1.cohspctrm(1,3,:)),'r');
```

PRO version Are you a developer? Try out the HTML to PDF API pdfcrowd.com

```
plot(g1.freq,squeeze(coh1.cohspctrm(2,3,:)),'k');
plot(g1.freq,squeeze(pcoh1.cohspctrm(1,2,:)),'m');
legend('1-2','1-3','2-3', '1-2 | 3');
title('Nonparametric Coherence spectrum');

figure;plot(g1.freq,squeeze(coh2.cohspctrm(1,2,:))); hold on
plot(g1.freq,squeeze(coh2.cohspctrm(1,3,:)),'r');
plot(g1.freq,squeeze(coh2.cohspctrm(2,3,:)),'k');
legend('1-2','1-3','2-3');
title('Parametric Coherence spectrum');


figure;plot(g1.freq,squeeze(psi1.psispctrm(1,2,:))); hold on;
plot(g1.freq,squeeze(psi1.psispctrm(1,3,:)),'r');
plot(g1.freq,squeeze(psi1.psispctrm(2,3,:)),'k');
legend('1->2','1->3','3->2');title('PSI nonparametric');

figure;plot(g1.freq,squeeze(g1.grangerspctrm(1,2,:)));hold on
plot(g1.freq,squeeze(g1.grangerspctrm(2,1,:)),'r');
plot(g1.freq,squeeze(g1.grangerspctrm(3,1,:)),'k');
plot(g1.freq,squeeze(g1.grangerspctrm(3,2,:)),'g');
plot(g1.freq,squeeze(g1.grangerspctrm(1,3,:)),'c');
plot(g1.freq,squeeze(g1.grangerspctrm(2,3,:)),'y');
title('Granger nonparametric estimates');legend('1->2','2->1','3->1','3->2','1->3','2->3')


figure; plot(g1.freq,squeeze(g2.grangerspctrm(1,2,:)));hold on;
plot(g1.freq,squeeze(g2.grangerspctrm(2,1,:)),'r');
plot(g1.freq,squeeze(g2.grangerspctrm(3,1,:)),'k');
plot(g1.freq,squeeze(g2.grangerspctrm(3,2,:)),'g');
plot(g1.freq,squeeze(g2.grangerspctrm(1,3,:)),'c');
plot(g1.freq,squeeze(g2.grangerspctrm(2,3,:)),'y');
legend('1->2','2->1','3->1','3->2','1->3','2->3');title('Granger parametric estimates ');
```

```
the call to "ft_connectivitysimulation" took 0 seconds
the input is raw data with 3 channels and 200 trials
Warning: the data does not contain a trial definition
Warning: reconstructing sampleinfo by assuming that the trials are consecutive
segments of a continuous recording
the call to "ft_selectdata" took 0 seconds
processing trials
```

PRO version Are you a developer? Try out the HTML to PDF API pdfcrowd.com

```
processing trial 200/200 nfft: 200 samples, datalength: 200 samples, 5 tapers

the call to "ft_freqanalysis" took 1 seconds
the input is freq data with 3 channels, 101 frequencybins and no timebins
the call to "ft_selectdata" took 0 seconds
the call to "ft_freqdescriptives" took 0 seconds
Warning: the data does not contain a trial definition
Warning: reconstructing sampleinfo by assuming that the trials are consecutive
segments of a continuous recording
the call to "ft_selectdata" took 0 seconds
preprocessing
preprocessing trial 200 from 200

the call to "ft_preprocessing" took 0 seconds
the call to "ft_redefinetrial" took 0 seconds
computing AR-model [-------------------------------------------------------|]
the call to "ft_mvaranalysis" took 0 seconds
the input is mvar data
Warning: could not determine dimord of "dof" in the following data
         dimord: 'chan_chan_lag'
          label: {3x1 cell}
         coeffs: [3x3x2 double]
       noisecov: [3x3 double]
            dof: 200
    fsampleorig: 200
            cfg: [1x1 struct]

not including "dof" in selection
the call to "ft_selectdata" took 0 seconds
computing MAR-model based TFR
processing timewindow 1 from 1

the call to "ft_freqanalysis_mvar" took 0 seconds
selection fourierspctrm along dimension 2
averaging crsspctrm over rpt
removing dimension rpt from crsspctrm
the call to "ft_connectivityanalysis" took 1 seconds
selection fourierspctrm along dimension 2
averaging crsspctrm over rpt
removing dimension rpt from crsspctrm
the call to "ft_connectivityanalysis" took 1 seconds
selection crsspctrm along dimensions 1 and 2
```

PRO version Are you a developer? Try out the HTML to PDF API pdfcrowd.com

```
selection transfer along dimensions 1 and 2
the call to "ft_connectivityanalysis" took 0 seconds
selection fourierspctrm along dimension 2
averaging crsspctrm over rpt
removing dimension rpt from crsspctrm
the call to "ft_connectivityanalysis" took 1 seconds
selection fourierspctrm along dimension 2
Warning: partialisation on single trial observations is not supported, removing
trial dimension
the call to "ft_connectivityanalysis" took 0 seconds
selection fourierspctrm along dimension 2
computing multivariate non-parametric spectral factorization on 3 channels
computing spectral factorization [-----------------------------------------]
the call to "ft_connectivityanalysis" took 1 seconds
selection crsspctrm along dimensions 1 and 2
selection transfer along dimensions 1 and 2
the call to "ft_connectivityanalysis" took 0 seconds
the call to "ft_connectivitysimulation" took 1 seconds
the call to "ft_selectdata" took 0 seconds
the input is raw data with 2 channels and 500 trials
Warning: the data does not contain a trial definition
Warning: reconstructing sampleinfo by assuming that the trials are consecutive
segments of a continuous recording
the call to "ft_selectdata" took 0 seconds
processing trials
processing trial 500/500 nfft: 200 samples, datalength: 200 samples, 5 tapers

the call to "ft_freqanalysis" took 1 seconds
the input is freq data with 2 channels, 101 frequencybins and no timebins
the call to "ft_selectdata" took 0 seconds
the call to "ft_freqdescriptives" took 0 seconds
selection fourierspctrm along dimension 2
averaging crsspctrm over rpt
removing dimension rpt from crsspctrm
the call to "ft_connectivityanalysis" took 2 seconds
selection fourierspctrm along dimension 2
averaging crsspctrm over rpt
removing dimension rpt from crsspctrm
the call to "ft_connectivityanalysis" took 2 seconds
selection fourierspctrm along dimension 2
averaging crsspctrm over rpt
removing dimension rpt from crsspctrm
```

PRO version Are you a developer? Try out the HTML to PDF API pdfcrowd.com

```
the call to "ft_connectivityanalysis" took 2 seconds
selection fourierspctrm along dimension 2
Warning: The field cfg.sfmethod is deprecated, please specify it as
cfg.granger.sfmethod instead of cfg.
computing pairwise non-parametric spectral factorization on 1 channel pairs
computing spectral factorization [----------------------------------------|]
the call to "ft_connectivityanalysis" took 0 seconds
the call to "ft_connectivitysimulation" took 0 seconds
the input is raw data with 3 channels and 200 trials
Warning: the data does not contain a trial definition
Warning: reconstructing sampleinfo by assuming that the trials are consecutive
segments of a continuous recording
the call to "ft_selectdata" took 0 seconds
processing trials
processing trial 200/200 nfft: 200 samples, datalength: 200 samples, 5 tapers

the call to "ft_freqanalysis" took 0 seconds
Warning: The option cfg.complex is deprecated, support is no longer guaranteed

the input is freq data with 3 channels, 101 frequencybins and no timebins
the call to "ft_selectdata" took 0 seconds
computing jackknife powspctrm
computing power... [-------------------------------------------------------|]
the call to "ft_freqdescriptives" took 0 seconds
the call to "ft_selectdata" took 0 seconds
preprocessing
preprocessing trial 200 from 200

the call to "ft_preprocessing" took 0 seconds
the call to "ft_redefinetrial" took 0 seconds
computing AR-model [-------------------------------------------------------|]
the call to "ft_mvaranalysis" took 0 seconds
the input is mvar data
Warning: could not determine dimord of "dof" in the following data
         dimord: 'chan_chan_lag'
          label: {3x1 cell}
         coeffs: [3x3x2 double]
       noisecov: [3x3 double]
            dof: 200
    fsampleorig: 200
            cfg: [1x1 struct]
```

PRO version Are you a developer? Try out the HTML to PDF API pdfcrowd.com

```
not including "dof" in selection
the call to "ft_selectdata" took 0 seconds
computing MAR-model based TFR
processing timewindow 1 from 1

the call to "ft_freqanalysis_mvar" took 0 seconds
selection fourierspctrm along dimension 2
averaging crsspctrm over rpt
removing dimension rpt from crsspctrm
the call to "ft_connectivityanalysis" took 1 seconds
selection fourierspctrm along dimension 2
averaging crsspctrm over rpt
removing dimension rpt from crsspctrm
the call to "ft_connectivityanalysis" took 1 seconds
selection crsspctrm along dimensions 1 and 2
selection transfer along dimensions 1 and 2
the call to "ft_connectivityanalysis" took 0 seconds
selection fourierspctrm along dimension 2
Warning: partialisation on single trial observations is not supported, removing
trial dimension
the call to "ft_connectivityanalysis" took 0 seconds
selection fourierspctrm along dimension 2
computing multivariate non-parametric spectral factorization on 3 channels
computing spectral factorization [---------------------------------------]
the call to "ft_connectivityanalysis" took 1 seconds
selection crsspctrm along dimensions 1 and 2
selection transfer along dimensions 1 and 2
the call to "ft_connectivityanalysis" took 0 seconds
```

PRO version Are you a developer? Try out the HTML to PDF API pdfcrowd.com

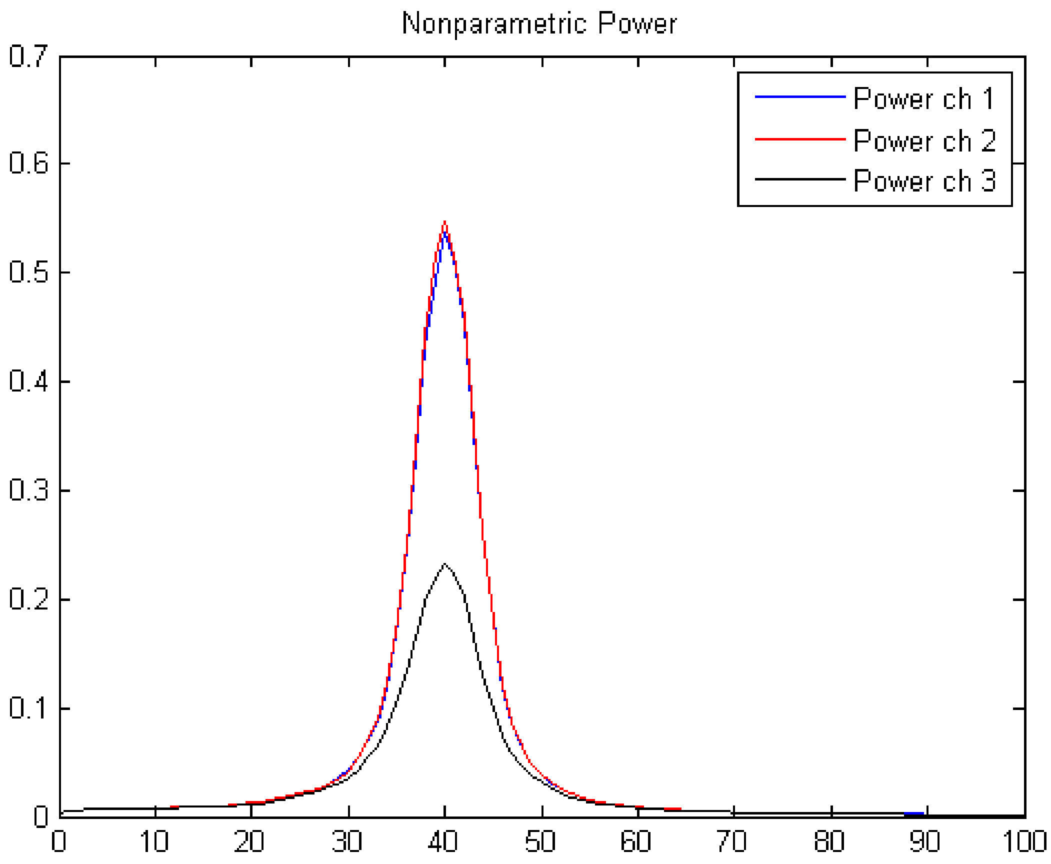

PRO version Are you a developer? Try out the HTML to PDF API pdfcrowd.com

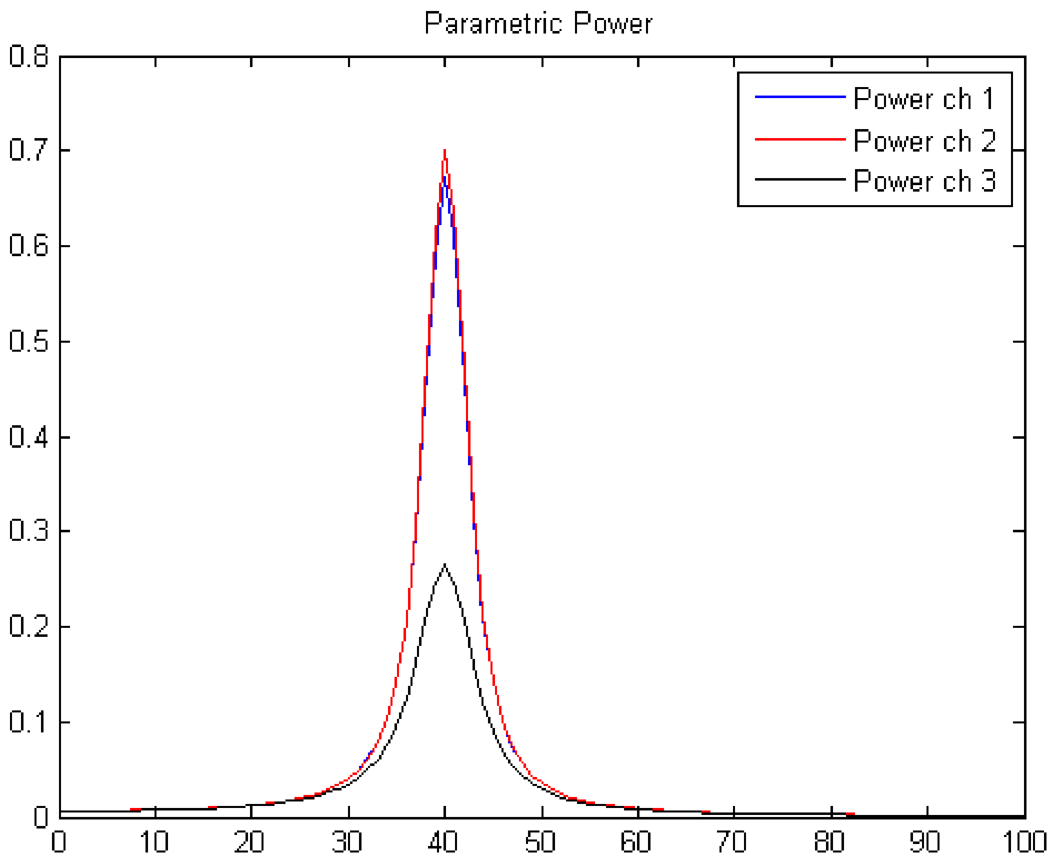

PRO version Are you a developer? Try out the HTML to PDF API pdfcrowd.com

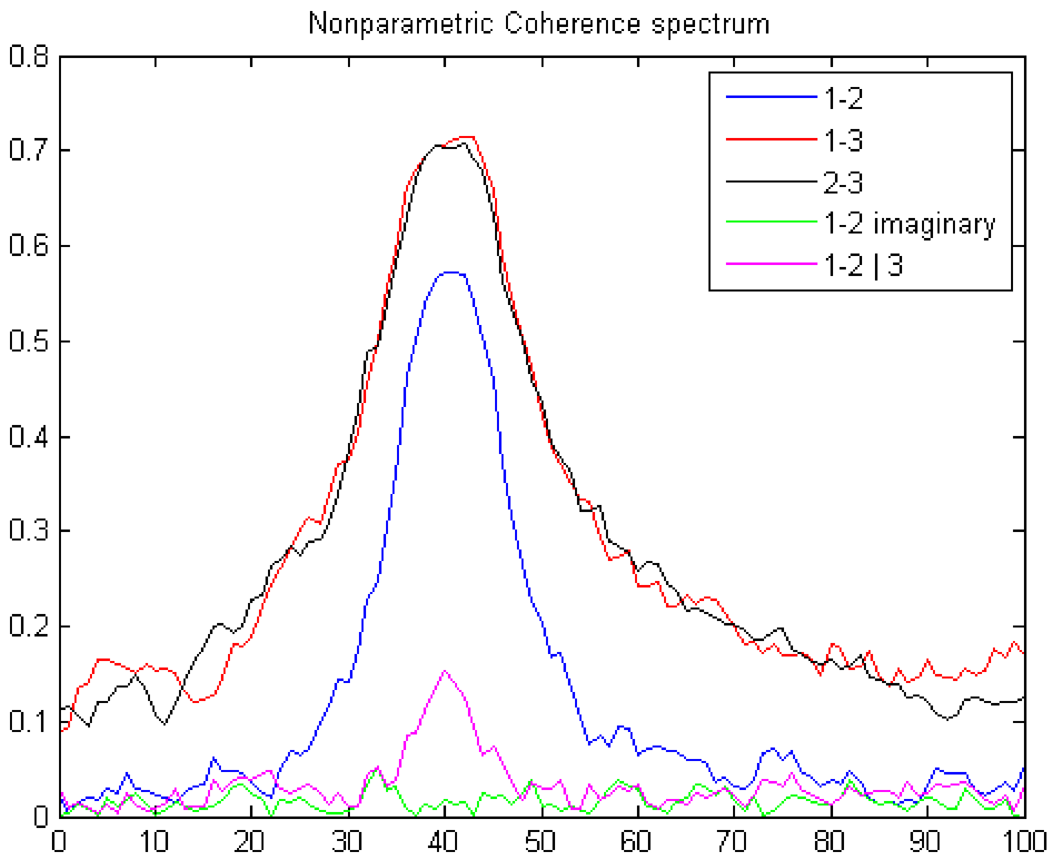

PRO version Are you a developer? Try out the HTML to PDF API pdfcrowd.com

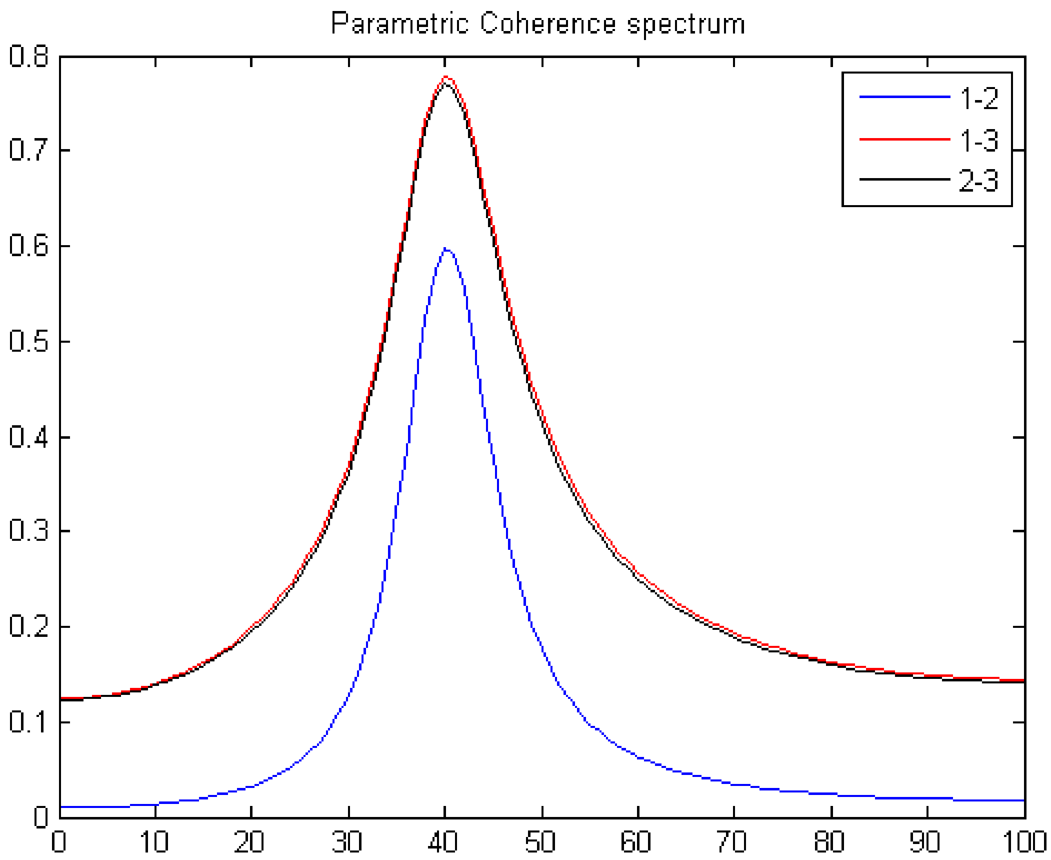

PRO version Are you a developer? Try out the HTML to PDF API pdfcrowd.com

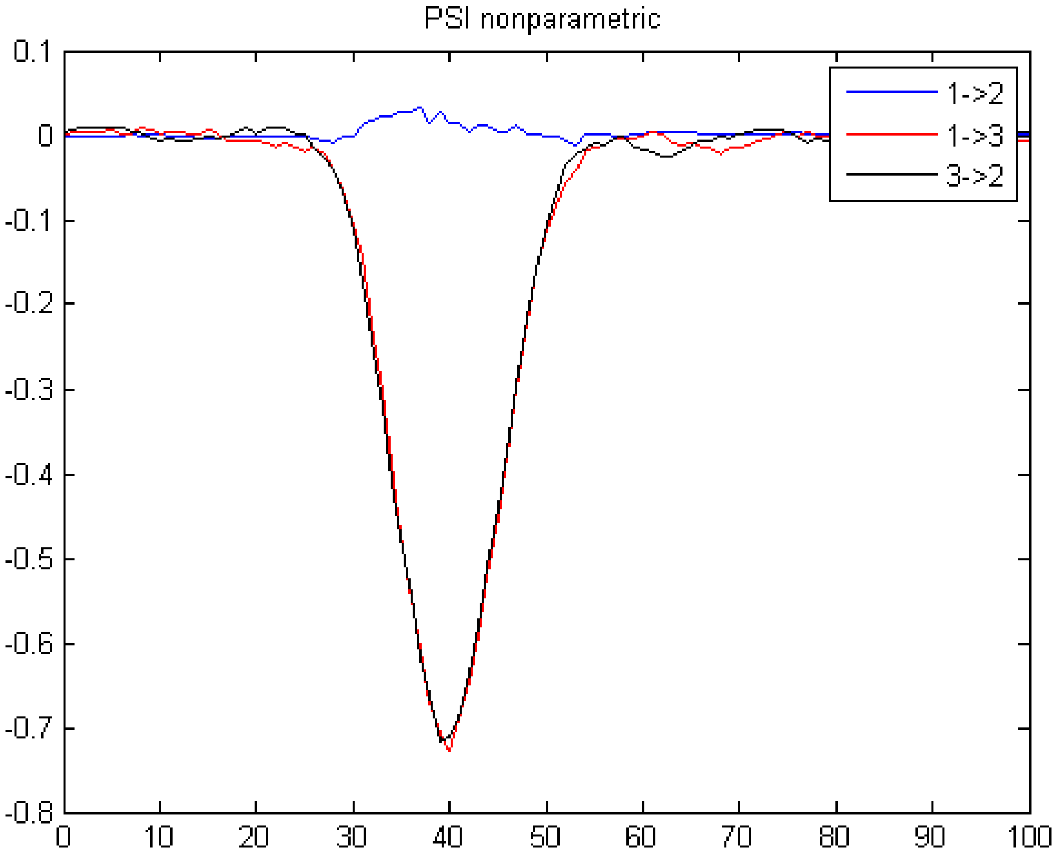

PRO version Are you a developer? Try out the HTML to PDF API pdfcrowd.com

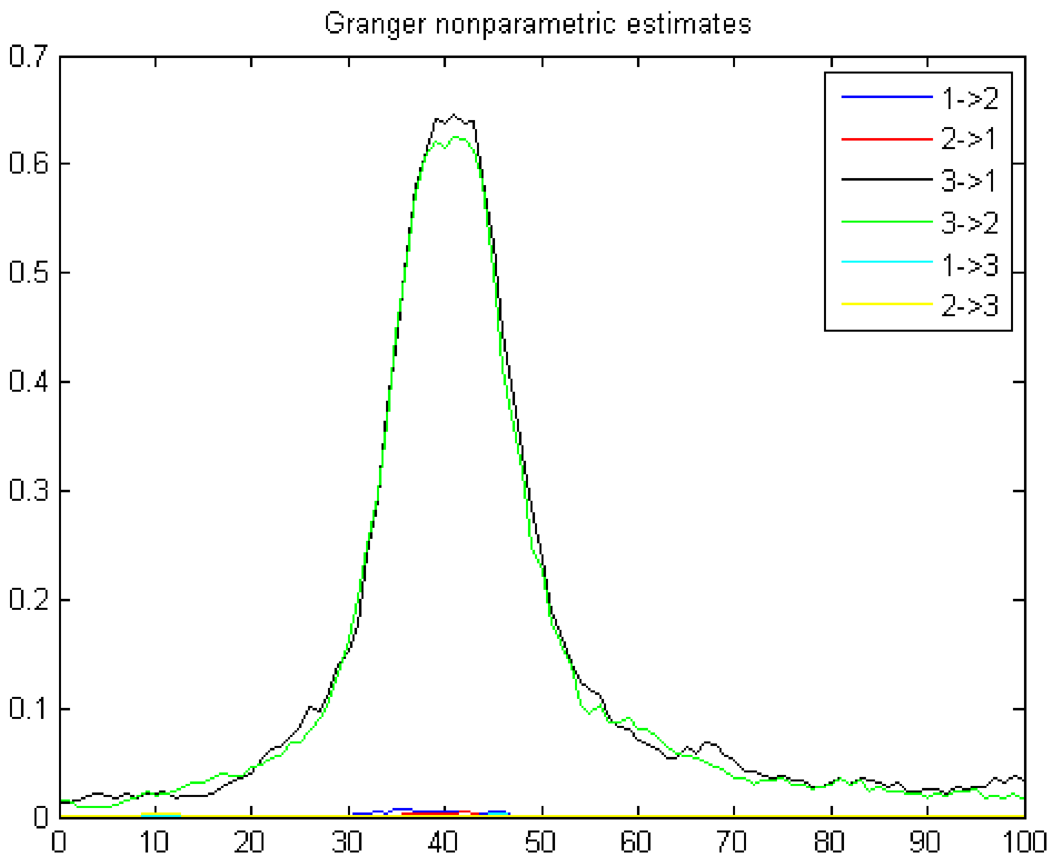

PRO version Are you a developer? Try out the HTML to PDF API pdfcrowd.com

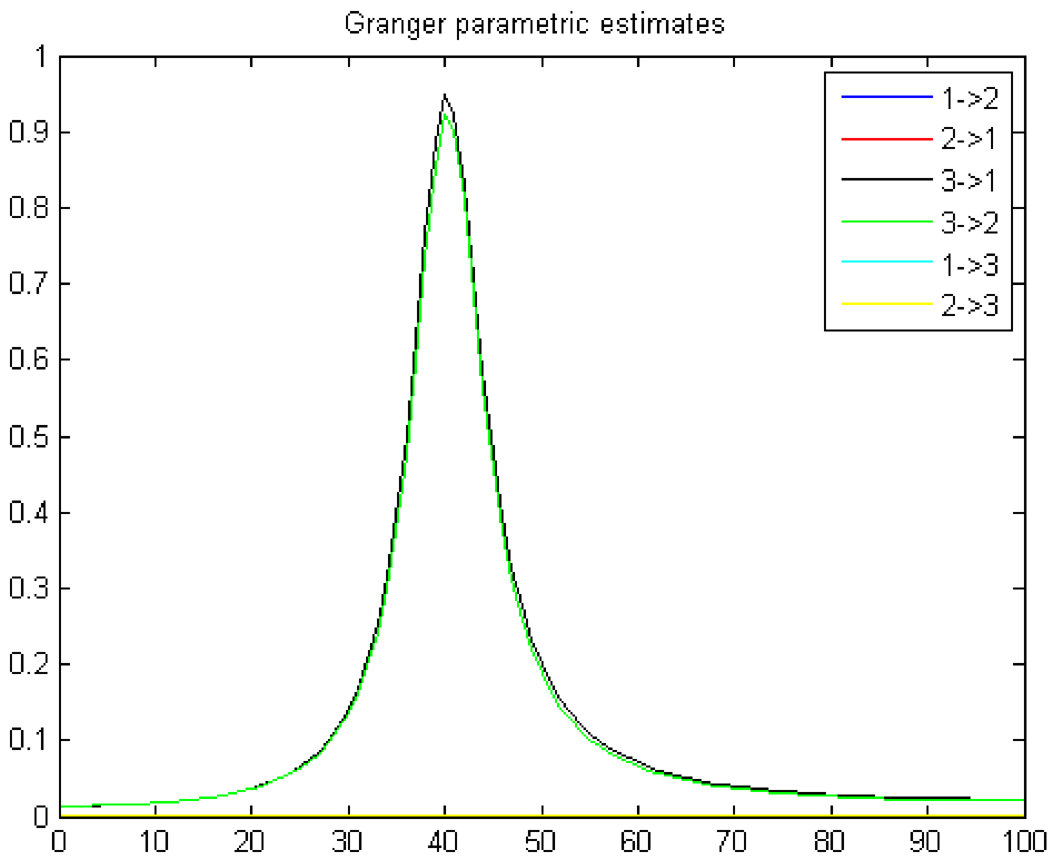

PRO version Are you a developer? Try out the HTML to PDF API pdfcrowd.com

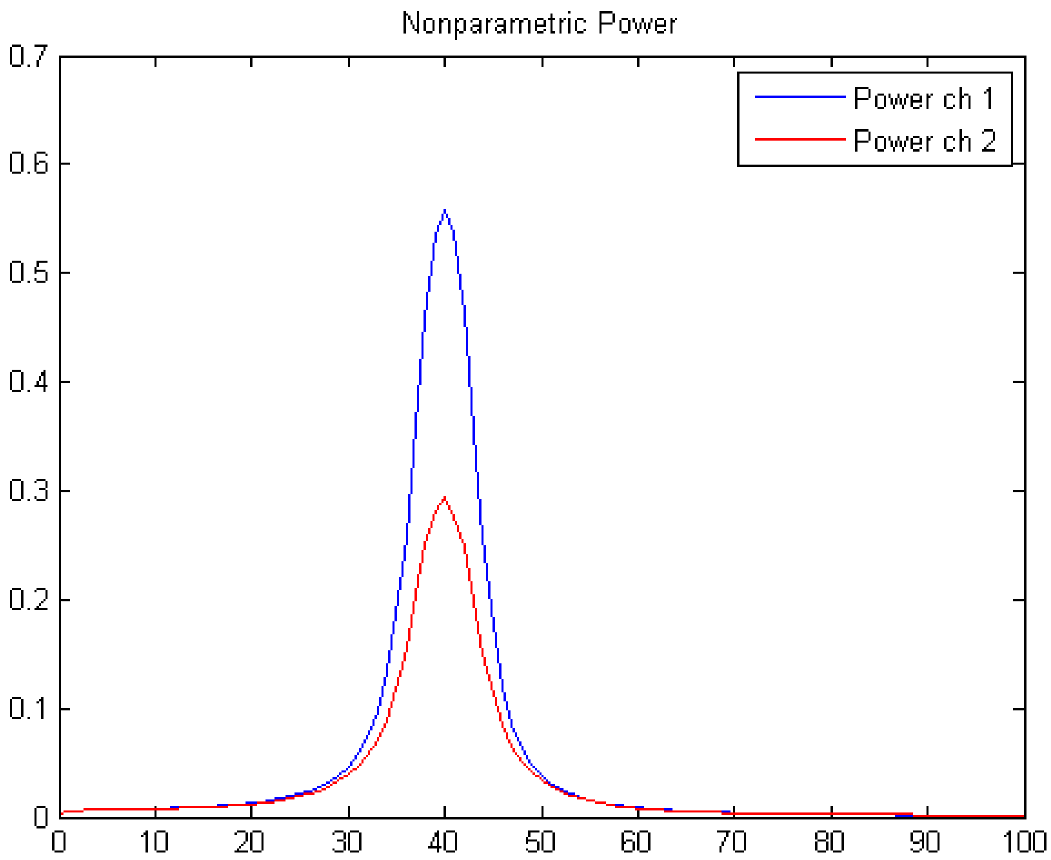

PRO version Are you a developer? Try out the HTML to PDF API pdfcrowd.com

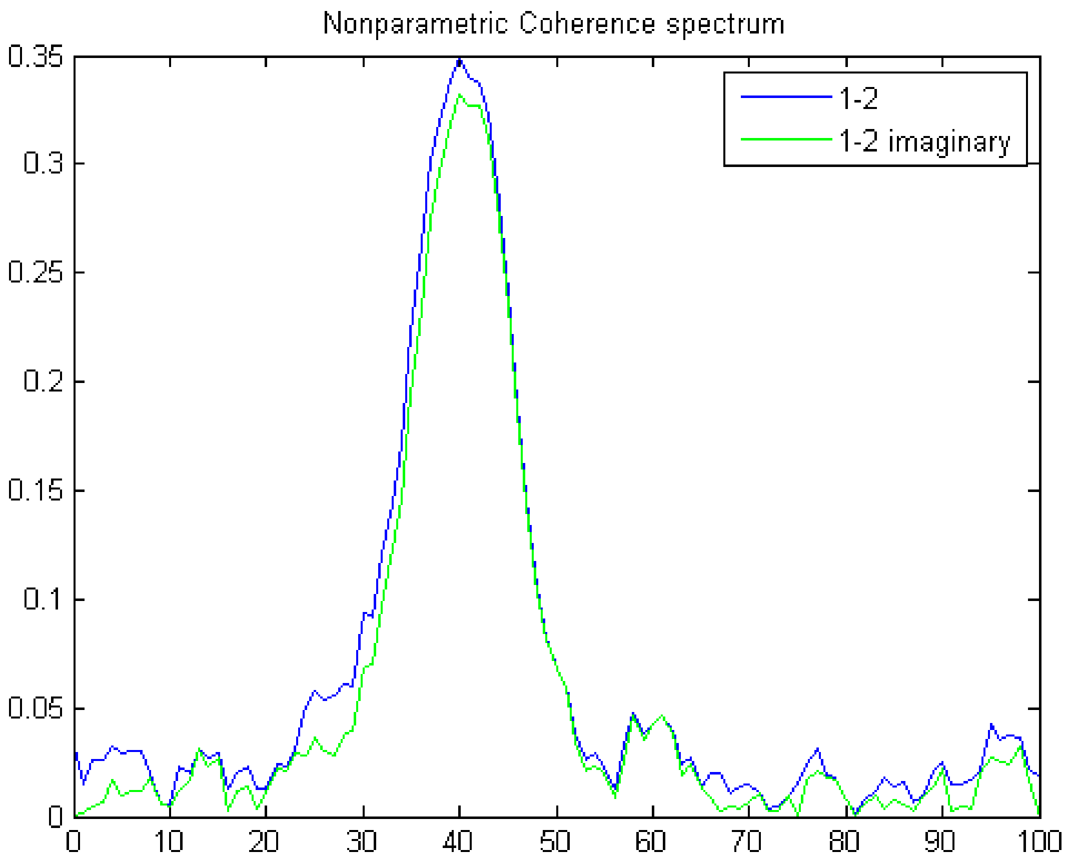

PRO version Are you a developer? Try out the HTML to PDF API pdfcrowd.com

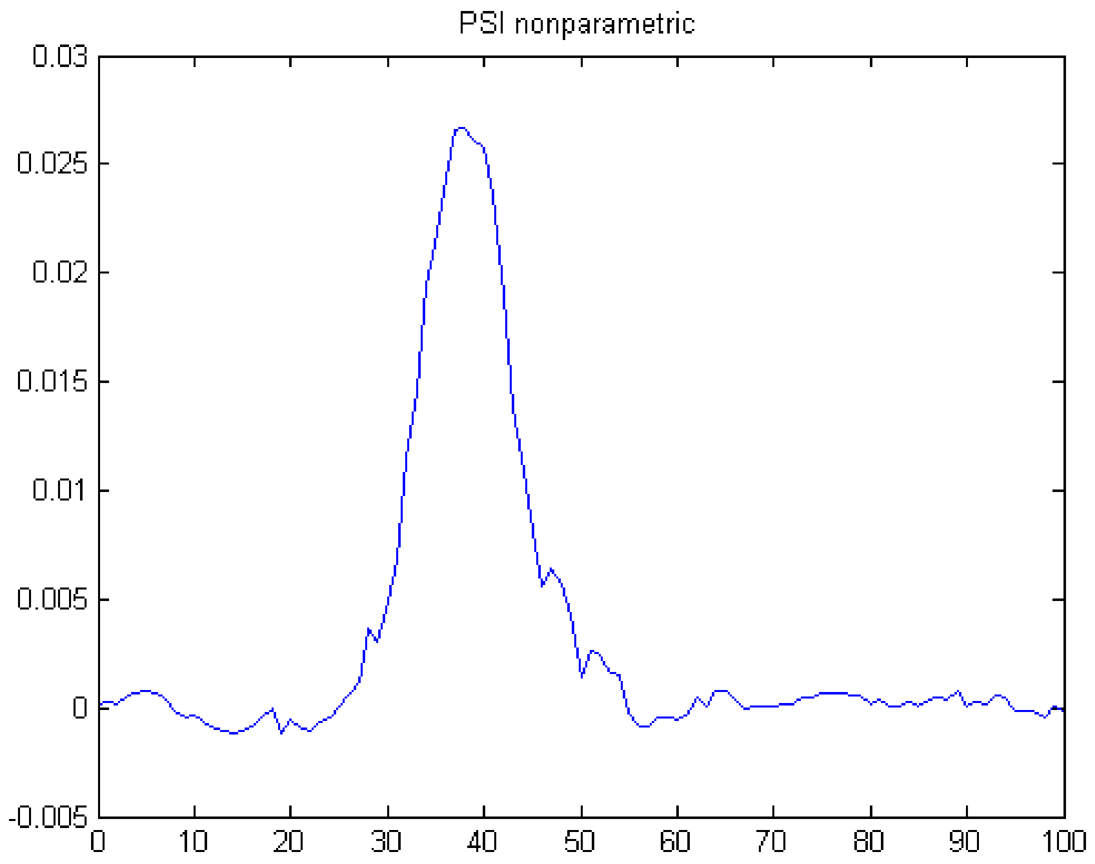

PRO version Are you a developer? Try out the HTML to PDF API pdfcrowd.com

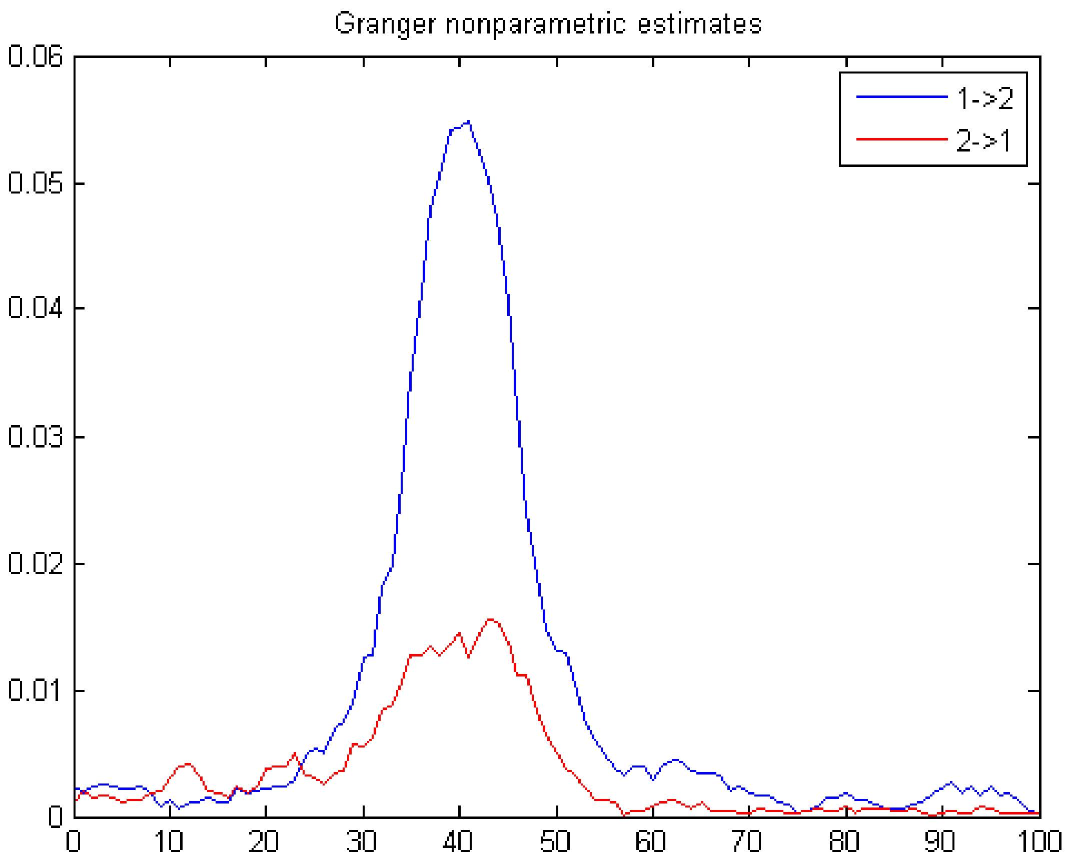

PRO version Are you a developer? Try out the HTML to PDF API pdfcrowd.com

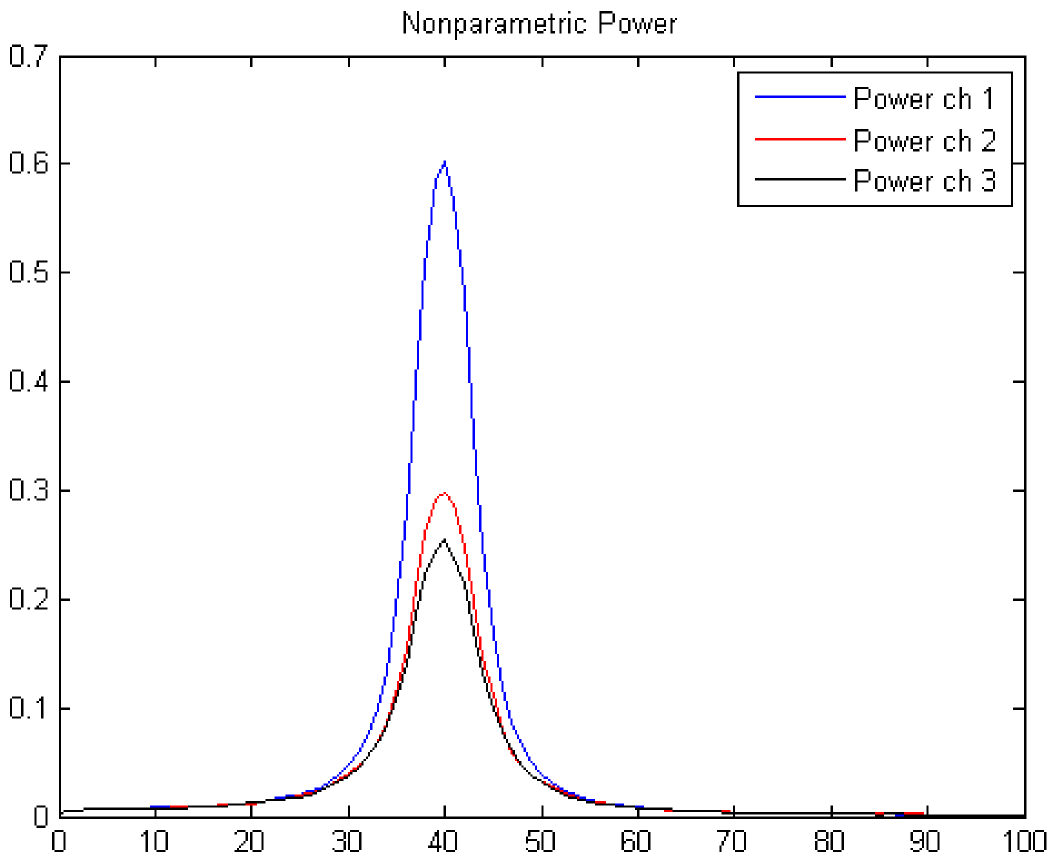

PRO version Are you a developer? Try out the HTML to PDF API pdfcrowd.com

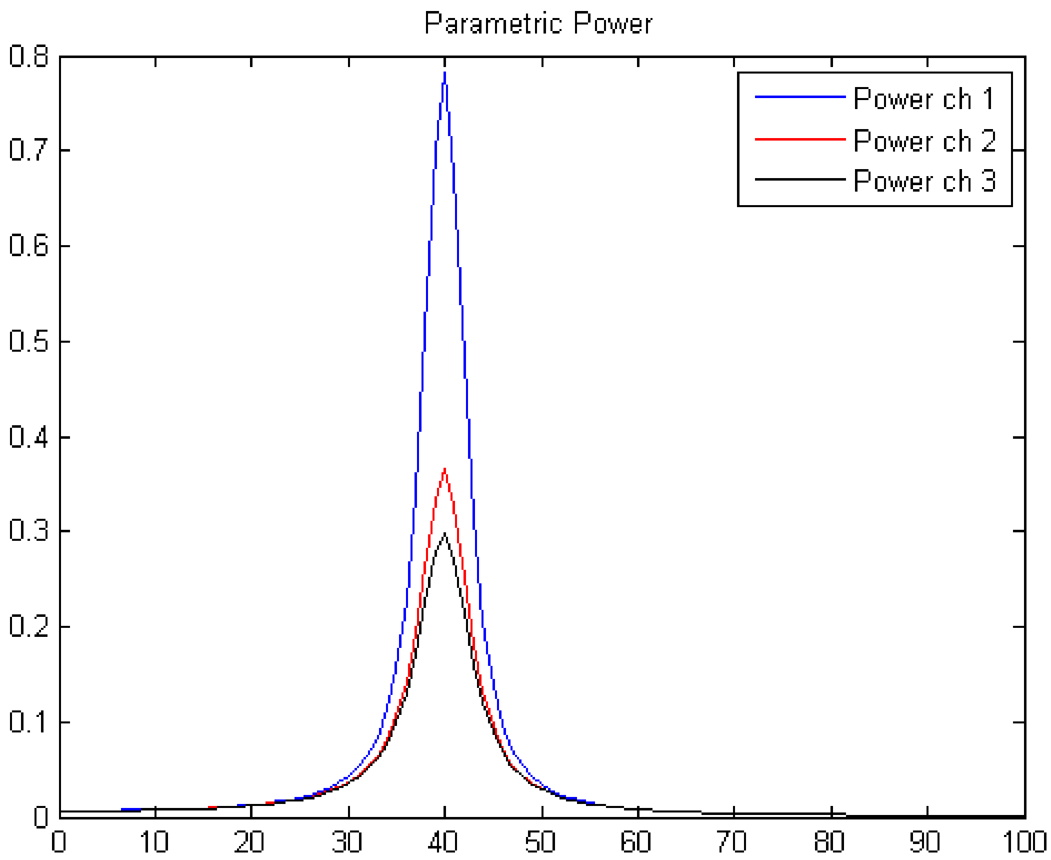

PRO version Are you a developer? Try out the HTML to PDF API pdfcrowd.com

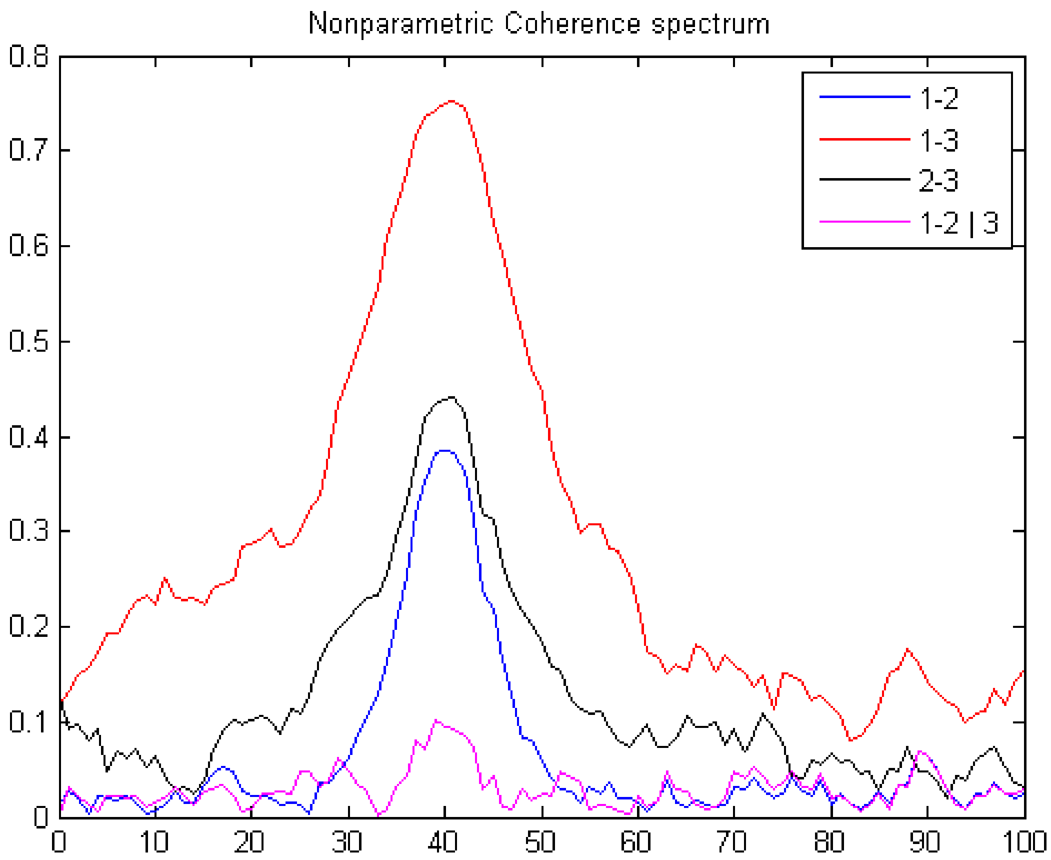

PRO version Are you a developer? Try out the HTML to PDF API pdfcrowd.com

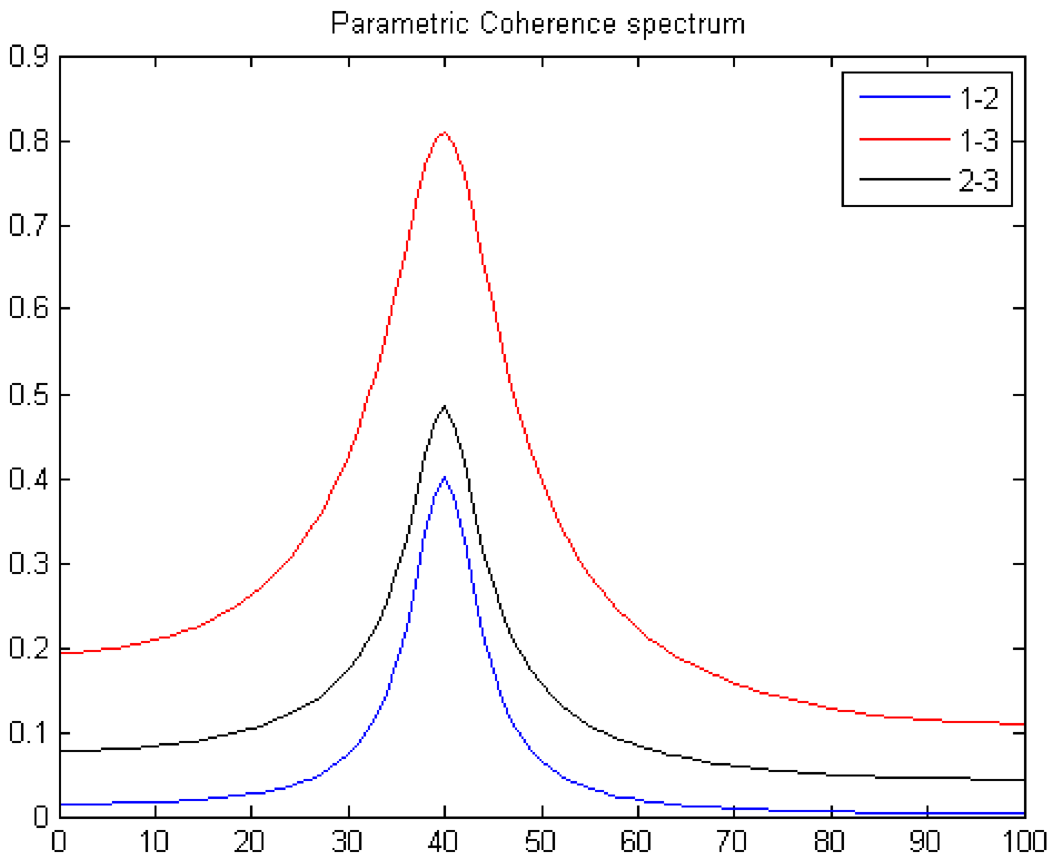

PRO version Are you a developer? Try out the HTML to PDF API pdfcrowd.com

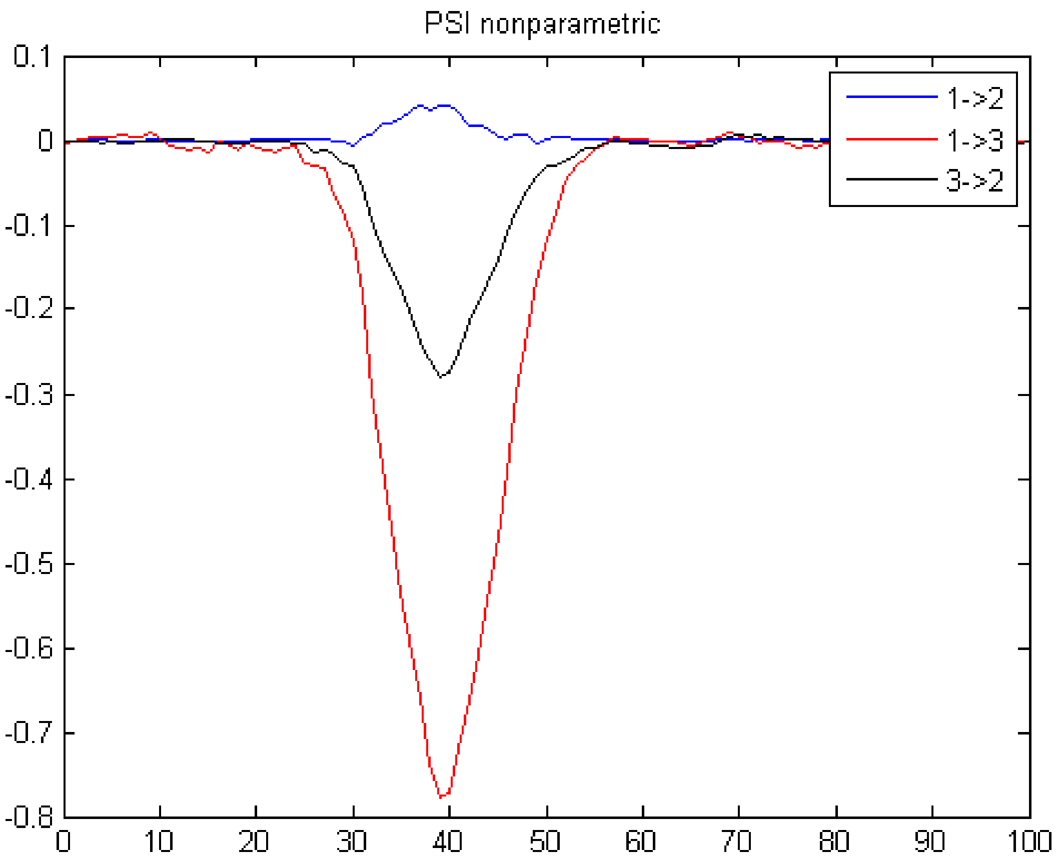

PRO version Are you a developer? Try out the HTML to PDF API pdfcrowd.com

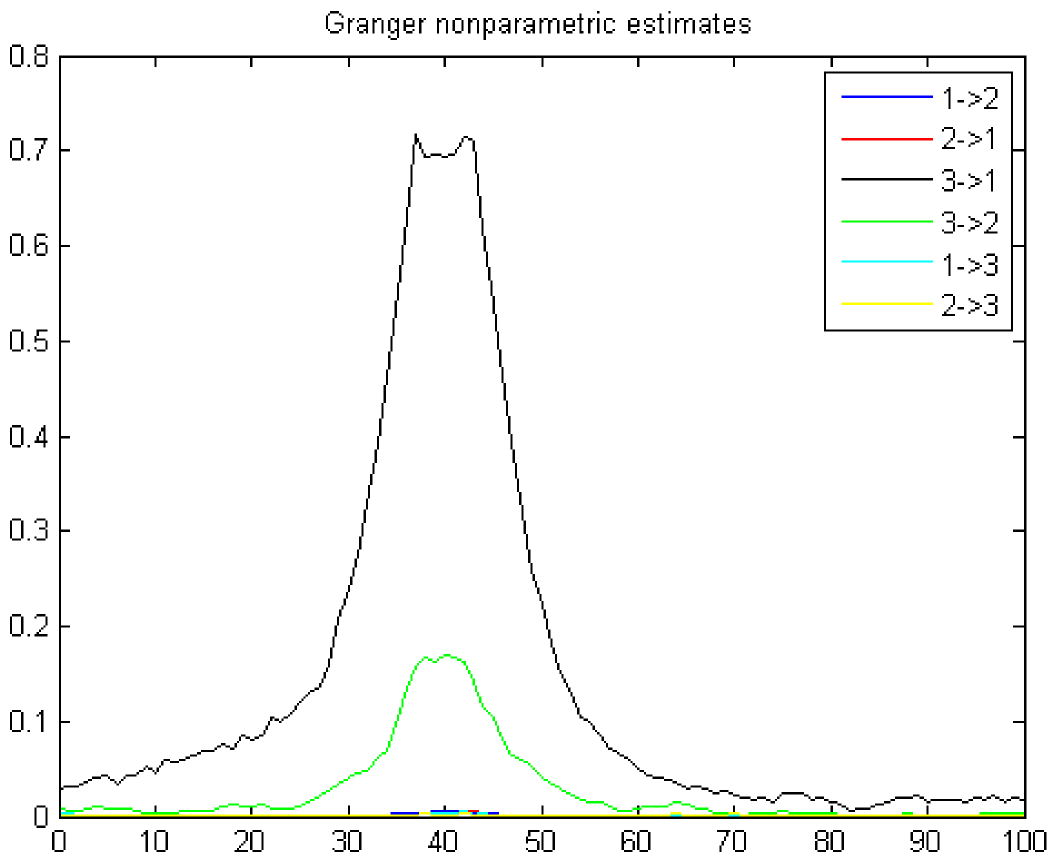

PRO version Are you a developer? Try out the HTML to PDF API pdfcrowd.com

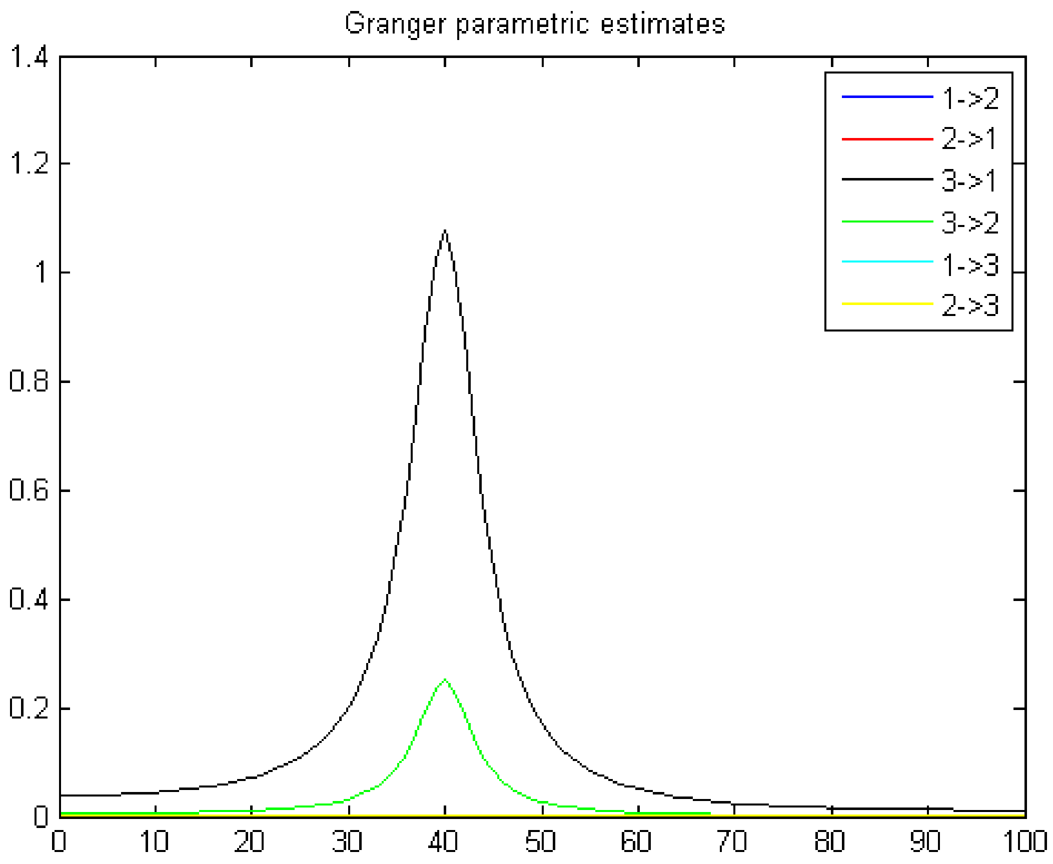

Published with MATLAB® R2013a

PRO version Are you a developer? Try out the HTML to PDF API pdfcrowd.com
