# Supplementary material for: A Tutorial Review of Functional Connectivity Analysis Methods and Their Interpretational Pitfalls
Source: Front Syst Neurosci. 2016 Jan 8;9:175. doi: 10.3389/fnsys.2015.00175 (PMC4705224; doi:10.3389/fnsys.2015.00175)
Supplement: Supplementary file 3 [file sim_commonreference.pdf]

```
%%%%%%%%%%%%%%%%%%%%%%%%%%%%%%%%%%%%%%%%%%%%%%%%%%
%Common reference, no bipolar derivation, no neuronal coupling (Figure 5A, red trace)
cfg = [];
cfg.method      = 'linear_mix';                 %apply a linear mixture of various components into data
cfg.ntrials     = 1000;                         %simulate 1000 trials
cfg.triallength = 1;                            %each trial is 1 second
cfg.fsample     = 1000;                         %sampling rate is 1000 Hz
cfg.nsignal     = 2;                            %simulate 2 unipolar channels
cfg.bpfilter    = 'yes';
cfg.bpfreq      = [35 55 ];                     %apply a band-pass filter between 35 and 55 Hz to the white noise
cfg.blc         = 'yes';                        %remove the mean of each trial

%Mixing matrix corresponds to:
%Source 1, Signal 2, Common reference, Coupling A, Coupling B

%Linearly mix a signal (third column, the common reference) which will be common to both channels
%Rows 1 and 2 correspond to data 1 and data 2 in Figure 5A
%Columns 1 and 2 correspond to sources 1 and 2 that are uniquely recorded by unipolar channels 1 and 2
%Column 3 is the common reference, which is equally present in both observed channels (rows)
%Columns 4 and 5 represents true neuronal coupling between the sources (set to zero in this case)

cfg.mix         = [0   1   1 0     0;
                   1   0   1 0     0];
%We can include delays between the different components if we wish. Here they are left to zero, because we assume an instantaneous mixture model
cfg.delay       = [0 0 0 0 0
                   0 0 0 0 0] ;

cfg.absnoise    = 1;   %the variance that will be added to each channel after linear mixing
data1nc = ft_connectivitysimulation(cfg);

%%%%%%%%%%%%%%%%%%%%%%%%%%%%%%%%%%%%%%%%%%%%%%%%%%
%Common reference, no bipolar derivation, with neuronal coupling (Figure 5A, blue trace)
%same base parameters as above
cfg = [];
cfg.method      = 'linear_mix';
cfg.ntrials     = 1000;
cfg.triallength = 1;
cfg.fsample     = 1000;
cfg.nsignal     = 2;
cfg.bpfilter    = 'yes';
cfg.bpfreq      = [35 55 ];
cfg.blc         = 'yes';
```

PRO version Are you a developer? Try out the HTML to PDF API pdfcrowd.com

```
%Mixing matrix columns corresponds to:
%Source 1, Signal 2, Common reference, Coupling A, Coupling B
%Rows 1 and 2 correspond to data 1 and data 2 in Figure 5A

%Columns 1 and 2 correspond to sources 1 and 2 that are uniquely recorded by unipolar channels 1 and 2
%Column 3 is the common reference, which is equally present in both observed channels (rows)
%Columns 4 and 5 represents true neuronal coupling between the sources (set to large values to simulate strong coupling)

cfg.mix         = [0     1   1 4     1;
                   1     0   1 2     3];

cfg.delay       = [0 0 0 0 0
                   0 0 0 0 0] ;

cfg.absnoise    = 1;
data1c = ft_connectivitysimulation(cfg);


%%%Calculate coherence for unipolar case - no coupling and with coupling

cfg        = [];
cfg.method = 'mtmfft';
cfg.taper  = 'dpss';
cfg.output = 'fourier';
cfg.tapsmofrq = 3;
cfg.foilim = [0 100];
freq1       = ft_freqanalysis(cfg, data1nc);
cfg           = [];
cfg.method    = 'coh';
cfg.complex   = 'abs';
coh1 = ft_connectivityanalysis(cfg, freq1);

cfg        = [];
cfg.method = 'mtmfft';
cfg.taper  = 'dpss'; %'rectwin'; %
cfg.output = 'fourier';
cfg.tapsmofrq = 3;
cfg.foilim = [0 100];
freq2       = ft_freqanalysis(cfg, data1c);
cfg           = [];
cfg.method    = 'coh';
cfg.complex   = 'abs';
coh2 = ft_connectivityanalysis(cfg, freq2);

figure; plot(coh1.freq,squeeze(coh1.cohspctrm(1,2,:)),'r'); hold on; plot(coh1.freq,squeeze(coh2.cohspctrm(1,2,:)));
```

PRO version Are you a developer? Try out the HTML to PDF API pdfcrowd.com

```
legend('No Coupling', 'Coupling');
title('Unipolar with common reference');


%%%%%%%%%%%%%%%%%%%%%%%%%%%%%%%%%%%%%%%%%%%%%%%%%%%%%%%%%%%%%%%%%%%%%%%%%%%%%%%%%%%
%BIPOLAR CASE
%Common reference, with bipolar derivation, no true neuronal coupling (Figure 5B, red trace)
cfg = [];
cfg.method      = 'linear_mix';
cfg.ntrials     = 1000;
cfg.triallength = 1;
cfg.fsample     = 1000;
cfg.nsignal     = 4;                    %simulate 4 unipolar channels
cfg.bpfilter    = 'yes';
cfg.bpfreq      = [35 55];
cfg.blc         = 'yes';

%Mixing matrix columns corresponds to:
%Source 1, Signal 2, Common reference, Source 3, Source 4
%Rows 1, 2, 3 and 4 correspond to unipolar data 1, 2, 3, and 4 in Figure 5b

%Linearly mix a signal which will be common to all 4 unipolar channels
%Columns 1 and 2 correspond to sources 1 and 2 that are uniquely recorded by unipolar channels 1 and 2, respectively
%Column 3 is the common reference, which is equally present in all 4 observed channels (rows)
%Columns 4 and 5 correspond to sources 3 and 4 that are uniquely recorded by unipolar channels 3 and 4, respectively
cfg.mix         = [1   0   1 0     0;
                   0   1   1 0     0;
                   0   0   1 1     0;
                   0   0   1 0     1;

                   ];

cfg.delay       = [0 0 0 0 0 ;
                   0 0 0 0 0 ;
                   0 0 0 0 0 ;
                   0 0 0 0 0 ;
                   ] ;

cfg.absnoise    = 1;

data4 = ft_connectivitysimulation(cfg);

%Calculate bipolar derivatives (unipolar channel 1 - channel 2 and channel 3 - channel 4)
%Rows of the montage.tra matrix correspond to resulting bipolar derivations
montage.tra = [1 -1 0 0;
```

PRO version Are you a developer? Try out the HTML to PDF API pdfcrowd.com

```
            0  0 1 -1];
montage.labelorg = data4.label;
montage.labelnew = {'bipolar1', 'bipolar2'};
databp1 = ft_apply_montage(data4, montage);


%%%%%%%%%%%%%%%%%%%%%%%%%%%%%%%%%%%%%%%%%%%%%%%%%%%
%Common reference, with bipolar derivation, with true neuronal coupling (Figure 5B, blue trace)
cfg = [];
cfg.method      = 'linear_mix';
cfg.ntrials     = 1000;
cfg.triallength = 1;
cfg.fsample     = 1000;
cfg.nsignal     = 4;
cfg.bpfilter    = 'yes';
cfg.bpfreq      = [35 55];
cfg.blc         = 'yes';


%Mixing matrix columns corresponds to:
%Source 1, Signal 2, Common reference, Source 3, Source 4, Coupling between sources
%Rows 1, 2, 3 and 4 correspond to unipolar data 1, 2, 3, and 4 in Figure 5b

%Linearly mix a signal which will be common to all 4 unipolar channels
%Columns 1 and 2 correspond to sources 1 and 2 that are uniquely recorded by unipolar channels 1 and 2, respectively
%Column 3 is the common reference, which is equally present in all 4 observed channels (rows)
%Columns 4 and 5 correspond to sources 3 and 4 that are uniquely recorded by unipolar channels 3 and 4, respectively
%Column 6 represent the true neuronal coupling between the sources (set to large values to simulate strong coupling)

cfg.mix         = [1 0   1 0    0 4
                   0 1   1 0    0 2
                   0 0   1 1    0 3
                   0 0   1 0    1 1
                   ];

cfg.delay       = [0 0 0 0 0 0;
                   0 0 0 0 0 0;
                   0 0 0 0 0 0;
                   0 0 0 0 0 0;
                   ] ;

cfg.absnoise    = 1;

data4 = ft_connectivitysimulation(cfg);
```

PRO version Are you a developer? Try out the HTML to PDF API pdfcrowd.com

```
%Calculate bipolar derivatives (channel 1 - channel 2 and channel 3 - channel 4)
%Rows of the montage.tra matrix correspond to resulting bipolar derivations
montage.tra = [1 -1 0 0;
               0  0 1 -1];
montage.labelorg = data4.label;
montage.labelnew = {'bipolar1', 'bipolar2'};
databp2 = ft_apply_montage(data4, montage);


%%%Calculate coherence for bipolar case - no coupling and with coupling
cfg        = [];
cfg.method = 'mtmfft';
cfg.taper  = 'dpss';
cfg.output = 'fourier';
cfg.tapsmofrq = 3;
cfg.foilim = [0 100];
freq1       = ft_freqanalysis(cfg, databp1);
cfg           = [];
cfg.method    = 'coh';
cfg.complex   = 'abs';
coh1 = ft_connectivityanalysis(cfg, freq1);

cfg        = [];
cfg.method = 'mtmfft';
cfg.taper  = 'dpss';
cfg.output = 'fourier';
cfg.tapsmofrq = 3;
cfg.foilim = [0 100];
freq2       = ft_freqanalysis(cfg, databp2);
cfg           = [];
cfg.method    = 'coh';
cfg.complex   = 'abs';
coh2 = ft_connectivityanalysis(cfg, freq2);

figure; plot(coh1.freq,squeeze(coh1.cohspctrm(1,2,:)),'r'); hold on; plot(coh1.freq,squeeze(coh2.cohspctrm(1,2,:)));
legend('No Coupling', 'Coupling');
title('Bipolar recordings with common reference');



%%%%%%%%%%%%%%%%%%%%%%%%%%%%%%%%%%%%%%%%%%%%%%%%%%%%%%%%%%%
%UNIPOLAR case, with seperate references
%Unique references, no bipolar derivation, no neuronal coupling (Figure 5C, red trace)
cfg = [];
cfg.method      = 'linear_mix';               %apply a linear mixture of various components into data
```

PRO version Are you a developer? Try out the HTML to PDF API pdfcrowd.com

```
cfg.ntrials     = 1000;                       %simulate 1000 trials
cfg.triallength = 1;                          %each trial is 1 second
cfg.fsample     = 1000;                       %sampling rate is 1000 Hz
cfg.nsignal     = 2;                          %simulate 2 unipolar channels
cfg.bpfilter    = 'yes';
cfg.bpfreq      = [35 55 ];                   %apply a band-pass filter between 35 and 55 Hz to the white noise
cfg.blc         = 'yes';                      %remove the mean of each trial

%Mixing matrix corresponds to:
%Signal A, Signal B, Common part of both signals, Coupling A, Coupling B

%Columns 1 and 2 correspond to sources 1 and 2 that are uniquely recorded by unipolar channels 1 and 2, respectively
%Column 3 - no common reference - third column is zero
%Columns 4 and 5 are intentionally left blank, denoting that the shared component (true coupling) is zero
cfg.mix         = [0   1   0 0     0;
                   1   0   0 0     0];
%We can include delays between the different components if we wish. Here they are left to zero, because we assume an instantaneous mixture model
cfg.delay       = [0 0 0 0 0
                   0 0 0 0 0] ;

cfg.absnoise    = 1;   %the variance that will be added to each channel after linear mixing
data2nc = ft_connectivitysimulation(cfg);

%%%%%%%%%%%%%%%%%%%%%%%%%%%%%%%%%%%%%%%%%%%%%%%%%%
%Unique references, no bipolar derivation, with neuronal coupling (Figure 5C, blue trace)
%same base parameters as above
cfg = [];
cfg.method      = 'linear_mix';
cfg.ntrials     = 1000;
cfg.triallength = 1;
cfg.fsample     = 1000;
cfg.nsignal     = 2;
cfg.bpfilter    = 'yes';
cfg.bpfreq      = [35 55 ];
cfg.blc         = 'yes';

%Mixing matrix corresponds to:
%Columns 1 and 2 correspond to sources 1 and 2 that are uniquely recorded by unipolar channels 1 and 2, respectively
%Column 3 - no common reference - third column is zero
%Columns 4 and 5 denote that the shared component (true coupling) between sources

cfg.mix         = [0     1   0 4     1;
                   1     0   0 2     3];

cfg.delay       = [0 0 0 0 0
```

PRO version Are you a developer? Try out the HTML to PDF API pdfcrowd.com

```
                   0 0 0 0 0] ;

cfg.absnoise    = 1;
data2c = ft_connectivitysimulation(cfg);



%%%Calculate coherence for case of unipolar with unique references case - no coupling and with coupling

cfg        = [];
cfg.method = 'mtmfft';
cfg.taper  = 'dpss';
cfg.output = 'fourier';
cfg.tapsmofrq = 3;
cfg.foilim = [0 100];
freq1       = ft_freqanalysis(cfg, data2nc);
cfg           = [];
cfg.method    = 'coh';
cfg.complex   = 'abs';
coh1 = ft_connectivityanalysis(cfg, freq1);

cfg        = [];
cfg.method = 'mtmfft';
cfg.taper  = 'dpss'; %'rectwin'; %
cfg.output = 'fourier';
cfg.tapsmofrq = 3;
cfg.foilim = [0 100];
freq2       = ft_freqanalysis(cfg, data2c);
cfg           = [];
cfg.method    = 'coh';
cfg.complex   = 'abs';
coh2 = ft_connectivityanalysis(cfg, freq2);

figure; plot(coh1.freq,squeeze(coh1.cohspctrm(1,2,:)),'r'); hold on; plot(coh1.freq,squeeze(coh2.cohspctrm(1,2,:)));
legend('No Coupling', 'Coupling');
title('Unipolar recordings with seperate reference');
```

```
the call to "ft_connectivitysimulation" took 2 seconds
the call to "ft_connectivitysimulation" took 2 seconds
the input is raw data with 2 channels and 1000 trials
Warning: the data does not contain a trial definition
Warning: reconstructing sampleinfo by assuming that the trials are consecutive
segments of a continuous recording
the call to "ft_selectdata" took 0 seconds
```

PRO version Are you a developer? Try out the HTML to PDF API pdfcrowd.com

```
processing trials
processing trial 1000/1000 nfft: 1000 samples, datalength: 1000 samples, 5 tapers

the call to "ft_freqanalysis" took 2 seconds
selection fourierspctrm along dimension 2
averaging crsspctrm over rpt
removing dimension rpt from crsspctrm
the call to "ft_connectivityanalysis" took 7 seconds
the input is raw data with 2 channels and 1000 trials
Warning: the data does not contain a trial definition
Warning: reconstructing sampleinfo by assuming that the trials are consecutive
segments of a continuous recording
the call to "ft_selectdata" took 0 seconds
processing trials
processing trial 1000/1000 nfft: 1000 samples, datalength: 1000 samples, 5 tapers

the call to "ft_freqanalysis" took 1 seconds
selection fourierspctrm along dimension 2
averaging crsspctrm over rpt
removing dimension rpt from crsspctrm
the call to "ft_connectivityanalysis" took 7 seconds
the call to "ft_connectivitysimulation" took 3 seconds
processing trials
processing trial 1000 from 1000

the call to "ft_connectivitysimulation" took 3 seconds
processing trials
processing trial 1000 from 1000

the input is raw data with 2 channels and 1000 trials
Warning: the data does not contain a trial definition
Warning: reconstructing sampleinfo by assuming that the trials are consecutive
segments of a continuous recording
the call to "ft_selectdata" took 0 seconds
processing trials
processing trial 1000/1000 nfft: 1000 samples, datalength: 1000 samples, 5 tapers

the call to "ft_freqanalysis" took 2 seconds
selection fourierspctrm along dimension 2
averaging crsspctrm over rpt
removing dimension rpt from crsspctrm
the call to "ft_connectivityanalysis" took 7 seconds
the input is raw data with 2 channels and 1000 trials
Warning: the data does not contain a trial definition
Warning: reconstructing sampleinfo by assuming that the trials are consecutive
```

PRO version Are you a developer? Try out the HTML to PDF API pdfcrowd.com

```
segments of a continuous recording
the call to "ft_selectdata" took 0 seconds
processing trials
processing trial 1000/1000 nfft: 1000 samples, datalength: 1000 samples, 5 tapers

the call to "ft_freqanalysis" took 2 seconds
selection fourierspctrm along dimension 2
averaging crsspctrm over rpt
removing dimension rpt from crsspctrm
the call to "ft_connectivityanalysis" took 7 seconds
the call to "ft_connectivitysimulation" took 2 seconds
the call to "ft_connectivitysimulation" took 2 seconds
the input is raw data with 2 channels and 1000 trials
Warning: the data does not contain a trial definition
Warning: reconstructing sampleinfo by assuming that the trials are consecutive
segments of a continuous recording
the call to "ft_selectdata" took 0 seconds
processing trials
processing trial 1000/1000 nfft: 1000 samples, datalength: 1000 samples, 5 tapers

the call to "ft_freqanalysis" took 2 seconds
selection fourierspctrm along dimension 2
averaging crsspctrm over rpt
removing dimension rpt from crsspctrm
the call to "ft_connectivityanalysis" took 7 seconds
the input is raw data with 2 channels and 1000 trials
Warning: the data does not contain a trial definition
Warning: reconstructing sampleinfo by assuming that the trials are consecutive
segments of a continuous recording
the call to "ft_selectdata" took 0 seconds
processing trials
processing trial 1000/1000 nfft: 1000 samples, datalength: 1000 samples, 5 tapers

the call to "ft_freqanalysis" took 1 seconds
selection fourierspctrm along dimension 2
averaging crsspctrm over rpt
removing dimension rpt from crsspctrm
the call to "ft_connectivityanalysis" took 7 seconds
```

PRO version Are you a developer? Try out the HTML to PDF API pdfcrowd.com

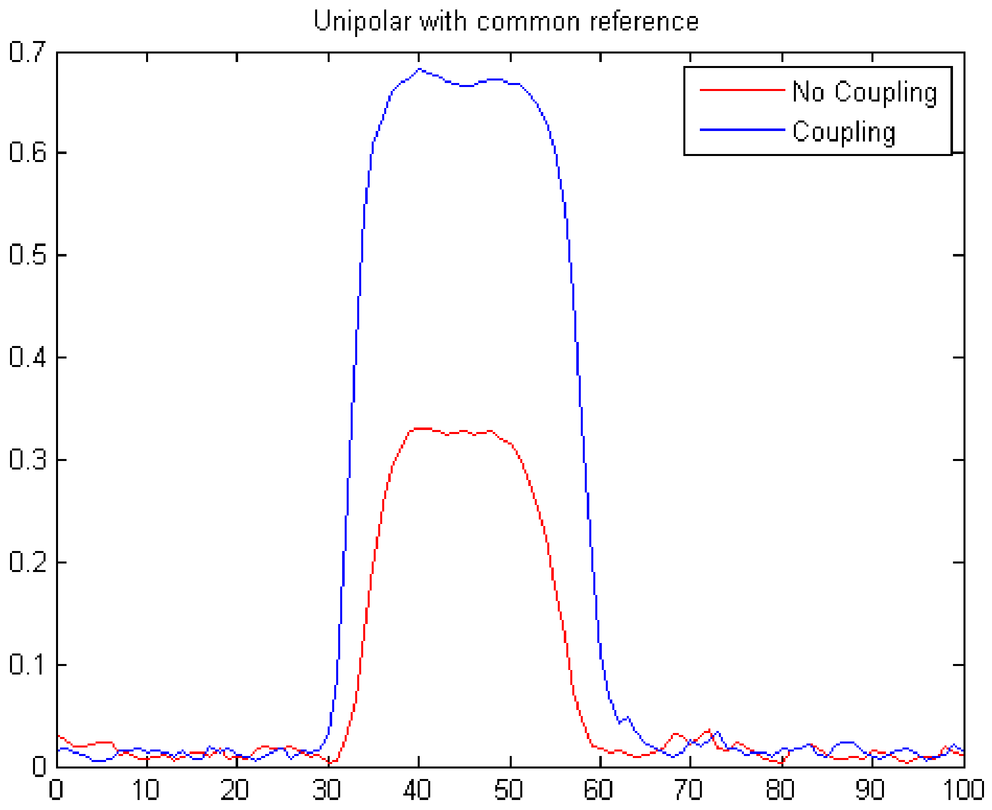

PRO version Are you a developer? Try out the HTML to PDF API pdfcrowd.com

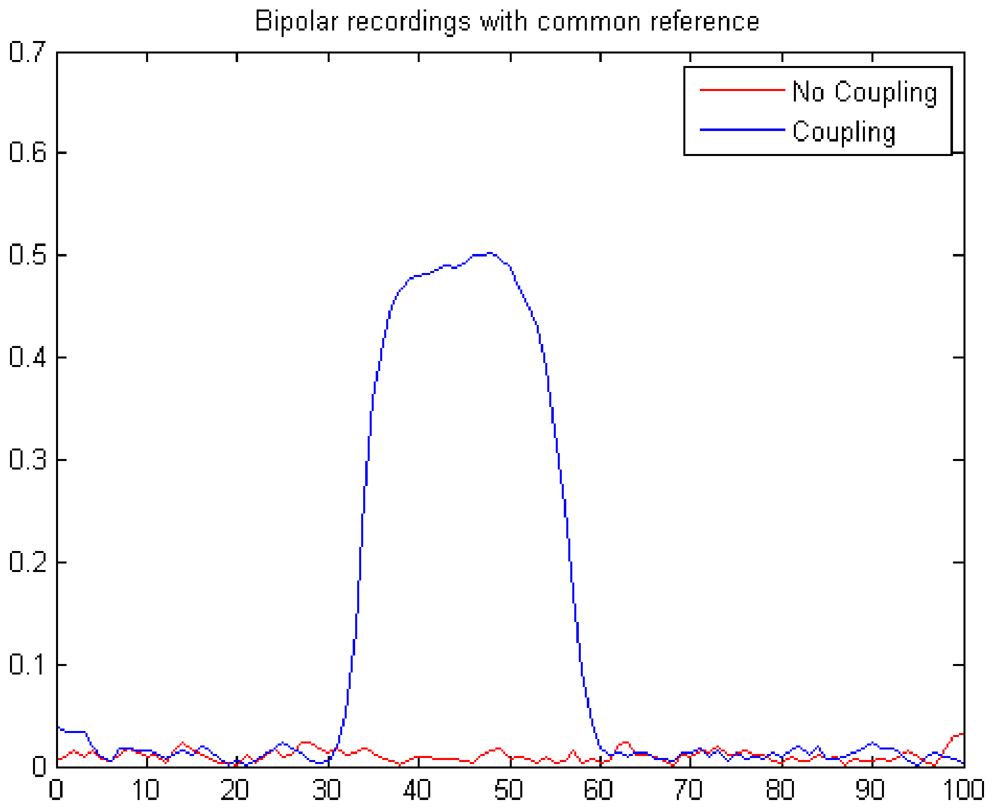

PRO version Are you a developer? Try out the HTML to PDF API pdfcrowd.com

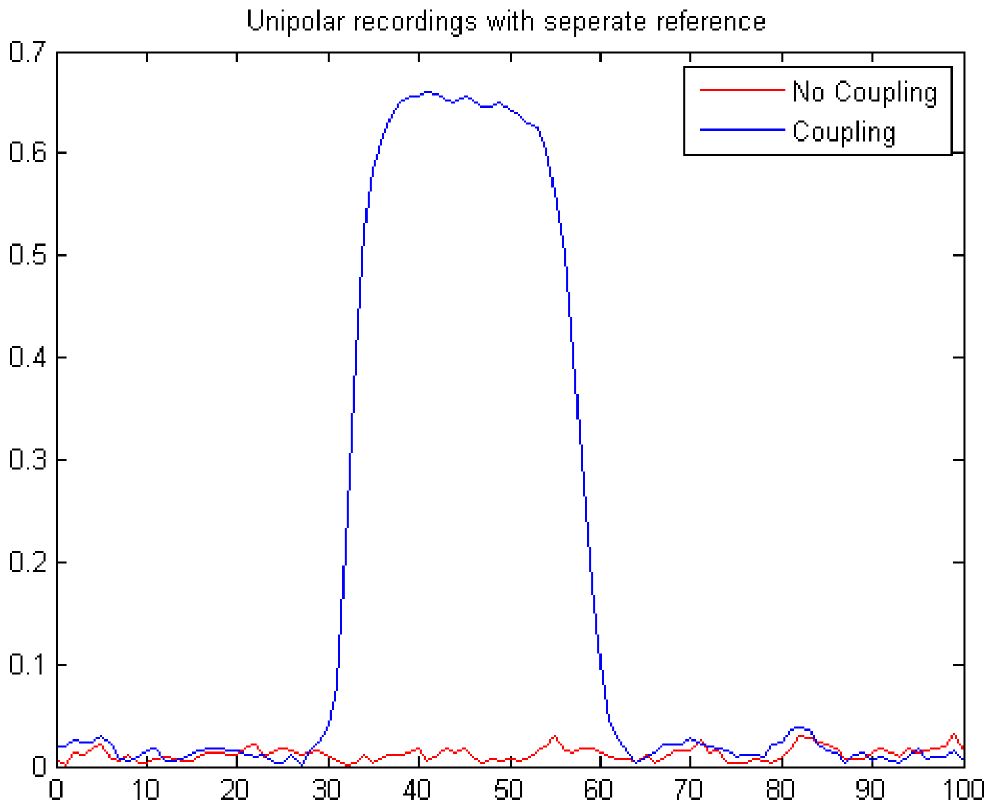

Published with MATLAB® R2013a

PRO version Are you a developer? Try out the HTML to PDF API pdfcrowd.com
