# Supplementary material for: A Tutorial Review of Functional Connectivity Analysis Methods and Their Interpretational Pitfalls
Source: Front Syst Neurosci. 2016 Jan 8;9:175. doi: 10.3389/fnsys.2015.00175 (PMC4705224; doi:10.3389/fnsys.2015.00175)
Supplement: Supplementary file 4 [file sim_samplesizebias.pdf]

```
% nsim = 100;  %number of realizations of the model to run
nsim = 5;

nfreqnp = 51;

%matrices to store the connectivity estimates
%dimensions are simulation number x frequency x type of simulation (the number of trials used to derive estimates - 5, 10, 50, 100, 500)
allcoh = zeros(nsim,nfreqnp, 5);
allppc = zeros(nsim,nfreqnp, 5);
allgra = zeros(nsim, 2, nfreqnp, 5);

for n = 1:nsim

    cfg = [];
    cfg.method      = 'ar';
    cfg.ntrials     = 5;
    cfg.triallength = 0.5;
    cfg.fsample     = 200;
    cfg.nsignal     = 2;
    cfg.bpfilter    = 'no';
    cfg.blc         = 'yes';
    cfg.params(:,:,1)      = [0.55   0.025;
                              0.025  0.55];
    cfg.params(:,:,2)      = [-0.8  -0.1;
                              -0.1  -0.8];
    cfg.noisecov     = [1 0.3;
                        0.3 1];

    data5 = ft_connectivitysimulation(cfg);
    cfg.ntrials     = 10;
    data10 = ft_connectivitysimulation(cfg);
    cfg.ntrials     = 50;
    data50 = ft_connectivitysimulation(cfg);
    cfg.ntrials     = 100;
    data100 = ft_connectivitysimulation(cfg);
    cfg.ntrials     = 500;
    data500 = ft_connectivitysimulation(cfg);

    %5 trial sim
    cfg        = [];
    cfg.method = 'mtmfft';
```

PRO version Are you a developer? Try out the HTML to PDF API pdfcrowd.com

```
    cfg.taper  = 'hanning';
    cfg.output = 'fourier';
    cfg.foilim = [0 100];
    freq       = ft_freqanalysis(cfg, data5);
    csd = ft_checkdata(freq, 'cmbrepresentation', 'fullfast');

    cfg           = [];
    cfg.method    = 'coh';
    cfg.complex   = 'abs';
    coh = ft_connectivityanalysis(cfg, csd);

    cfg           = [];
    cfg.method    = 'ppc';
    ppc = ft_connectivityanalysis(cfg, freq);

    cfg           = [];
    cfg.method    = 'granger';
    cfg.granger.sfmethod = 'bivariate';
    gra = ft_connectivityanalysis(cfg, csd);

    allcoh(n,:,1) = coh.cohspctrm(1,2,:);
    allppc(n,:,1) = ppc.ppcspctrm(1,2,:);
    allgra(n,1,:,1) = gra.grangerspctrm(1,:);
    allgra(n,2,:,1) = gra.grangerspctrm(2,:);

    %10 trial sim
    cfg        = [];
    cfg.method = 'mtmfft';
    cfg.taper  = 'hanning';
    cfg.output = 'fourier';
    cfg.foilim = [0 100];
    freq       = ft_freqanalysis(cfg, data10);
    csd = ft_checkdata(freq, 'cmbrepresentation', 'fullfast');

    cfg           = [];
    cfg.method    = 'coh';
    cfg.complex   = 'abs';
    coh = ft_connectivityanalysis(cfg, csd);

    cfg           = [];
    cfg.method    = 'ppc';
    ppc = ft_connectivityanalysis(cfg, freq);
```

PRO version Are you a developer? Try out the HTML to PDF API pdfcrowd.com

```
    cfg           = [];
    cfg.method    = 'granger';
    cfg.granger.sfmethod = 'bivariate';
    gra = ft_connectivityanalysis(cfg, csd);

    allcoh(n,:,2) = coh.cohspctrm(1,2,:);
    allppc(n,:,2) = ppc.ppcspctrm(1,2,:);
    allgra(n,1,:,2) = gra.grangerspctrm(1,:);
    allgra(n,2,:,2) = gra.grangerspctrm(2,:);

    %50 trial sim
    cfg        = [];
    cfg.method = 'mtmfft';
    cfg.taper  = 'hanning';
    cfg.output = 'fourier';
    cfg.foilim = [0 100];
    freq       = ft_freqanalysis(cfg, data50);

    csd = ft_checkdata(freq, 'cmbrepresentation', 'fullfast');

    cfg           = [];
    cfg.method    = 'coh';
    cfg.complex   = 'abs';
    coh = ft_connectivityanalysis(cfg, csd);

    cfg           = [];
    cfg.method    = 'ppc';
    ppc = ft_connectivityanalysis(cfg, freq);

    cfg           = [];
    cfg.method    = 'granger';
    cfg.granger.sfmethod = 'bivariate';
    gra = ft_connectivityanalysis(cfg, csd);

    allcoh(n,:,3) = coh.cohspctrm(1,2,:);
    allppc(n,:,3) = ppc.ppcspctrm(1,2,:);
    allgra(n,1,:,3) = gra.grangerspctrm(1,:);
    allgra(n,2,:,3) = gra.grangerspctrm(2,:);

    %100 trial sim
    cfg        = [];
    cfg.method = 'mtmfft';
    cfg.taper  = 'hanning';
```

PRO version Are you a developer? Try out the HTML to PDF API pdfcrowd.com

```
    cfg.output = 'fourier';
    cfg.foilim = [0 100];
    freq       = ft_freqanalysis(cfg, data100);
    csd = ft_checkdata(freq, 'cmbrepresentation', 'fullfast');

    cfg           = [];
    cfg.method    = 'coh';
    cfg.complex   = 'abs';
    coh = ft_connectivityanalysis(cfg, csd);

    cfg           = [];
    cfg.method    = 'ppc';
    ppc = ft_connectivityanalysis(cfg, freq);

    cfg           = [];
    cfg.method    = 'granger';
    cfg.granger.sfmethod = 'bivariate';
    gra = ft_connectivityanalysis(cfg, csd);

    allcoh(n,:,4) = coh.cohspctrm(1,2,:);
    allppc(n,:,4) = ppc.ppcspctrm(1,2,:);
    allgra(n,1,:,4) = gra.grangerspctrm(1,:);
    allgra(n,2,:,4) = gra.grangerspctrm(2,:);


    %500 trial sim
    cfg        = [];
    cfg.method = 'mtmfft';
    cfg.taper  = 'hanning';
    cfg.output = 'fourier';
    cfg.foilim = [0 100];
    freq       = ft_freqanalysis(cfg, data500);

    csd = ft_checkdata(freq, 'cmbrepresentation', 'fullfast');

    cfg           = [];
    cfg.method    = 'coh';
    cfg.complex   = 'abs';
    coh = ft_connectivityanalysis(cfg, csd);

    cfg           = [];
    cfg.method    = 'ppc';
```

PRO version Are you a developer? Try out the HTML to PDF API pdfcrowd.com

```
    ppc = ft_connectivityanalysis(cfg, freq);

    cfg           = [];
    cfg.method    = 'granger';
    cfg.granger.sfmethod = 'bivariate';
    gra = ft_connectivityanalysis(cfg, csd);

    allcoh(n,:,5) = coh.cohspctrm(1,2,:);
    allppc(n,:,5) = ppc.ppcspctrm(1,2,:);
    allgra(n,1,:,5) = gra.grangerspctrm(1,:);
    allgra(n,2,:,5) = gra.grangerspctrm(2,:);

    disp(['finished simulation ' int2str(n)])

end


allcoh_avgfreq = squeeze(sum(allcoh(:,:,:), 2));
allppc_avgfreq = squeeze(sum(allppc(:,:,:), 2));
allgra_avgfreq = squeeze(sum(allgra(:,1,:,:), 3) + sum(allgra(:,2,:,:), 3));

figure; errorbar(1:5, mean(allcoh_avgfreq), std(allcoh_avgfreq), 'r');
title('Coherence as a function of trial number')
set(gca, 'XTick', [1:5])
set(gca, 'XTickLabel', {'5 trials', '10 trials','50 trials', '100 trials', '500 trials'} )

figure; errorbar(1:5, mean(allppc_avgfreq), std(allppc_avgfreq), 'r');
title('PPC as a function of trial number')
set(gca, 'XTick', [1:5])
set(gca, 'XTickLabel', {'5 trials', '10 trials','50 trials', '100 trials', '500 trials'} )

figure;  errorbar(1:5, mean(allgra_avgfreq), std(allgra_avgfreq), 'r');
title('Granger causality as a function of trial number')
set(gca, 'XTick', [1:5])
set(gca, 'XTickLabel', {'5 trials', '10 trials','50 trials', '100 trials', '500 trials'} )


figure; plot(gra.freq, squeeze(mean(allgra(:,1,:,1)) + mean(allgra(:,2,:,1))),'r')
hold on; plot(gra.freq, squeeze(mean(allgra(:,1,:,2)) + mean(allgra(:,2,:,2))), 'b')
plot(gra.freq, squeeze(mean(allgra(:,1,:,3)) + mean(allgra(:,2,:,3))), 'k')
plot(gra.freq, squeeze(mean(allgra(:,1,:,4)) + mean(allgra(:,2,:,4))), 'm')
plot(gra.freq, squeeze(mean(allgra(:,1,:,5)) + mean(allgra(:,2,:,5))), 'c')
legend('5 trials', '10 trials', '50 trials', '100 trials', '500 trials')
```
PRO version Are you a developer? Try out the HTML to PDF API pdfcrowd.com

```
title('Non-parametric total GC (1->2 + 2->1) by trial number')

figure; plot(coh.freq, squeeze(mean(allcoh(:,:,1))),'r')
hold on; plot(coh.freq, squeeze(mean(allcoh(:,:,2))), 'b')
plot(coh.freq, squeeze(mean(allcoh(:,:,3))), 'k')
plot(coh.freq, squeeze(mean(allcoh(:,:,4))), 'm')
plot(coh.freq, squeeze(mean(allcoh(:,:,5))), 'c')
legend('5 trials', '10 trials', '50 trials', '100 trials', '500 trials')
title('Non-parametric coherence by trial number')

figure; plot(coh.freq, squeeze(mean(allppc(:,:,1))),'r')
hold on; plot(coh.freq, squeeze(mean(allppc(:,:,2))), 'b')
plot(coh.freq, squeeze(mean(allppc(:,:,3))), 'k')
plot(coh.freq, squeeze(mean(allppc(:,:,4))), 'm')
plot(coh.freq, squeeze(mean(allppc(:,:,5))), 'c')
legend('5 trials', '10 trials', '50 trials', '100 trials', '500 trials')
title('PPC by trial number')
```

```
the call to "ft_connectivitysimulation" took 0 seconds
the call to "ft_connectivitysimulation" took 0 seconds
the call to "ft_connectivitysimulation" took 0 seconds
the call to "ft_connectivitysimulation" took 0 seconds
the call to "ft_connectivitysimulation" took 0 seconds
the input is raw data with 2 channels and 5 trials
Warning: the data does not contain a trial definition
Warning: reconstructing sampleinfo by assuming that the trials are consecutive
segments of a continuous recording
the call to "ft_selectdata" took 0 seconds
processing trials
processing trial 5/5 nfft: 100 samples, datalength: 100 samples, 1 tapers

the call to "ft_freqanalysis" took 1 seconds
selection crsspctrm along dimensions 1 and 2
the call to "ft_connectivityanalysis" took 0 seconds
selection fourierspctrm along dimension 2
the call to "ft_connectivityanalysis" took 0 seconds
selection crsspctrm along dimensions 1 and 2
computing pairwise non-parametric spectral factorization on 1 channel pairs
computing spectral factorization [----------------------------------------|]
the call to "ft_connectivityanalysis" took 0 seconds
the input is raw data with 2 channels and 10 trials
```

PRO version Are you a developer? Try out the HTML to PDF API pdfcrowd.com

```
Warning: the data does not contain a trial definition
Warning: reconstructing sampleinfo by assuming that the trials are consecutive
segments of a continuous recording
the call to "ft_selectdata" took 0 seconds
processing trials
processing trial 10/10 nfft: 100 samples, datalength: 100 samples, 1 tapers

the call to "ft_freqanalysis" took 0 seconds
selection crsspctrm along dimensions 1 and 2
the call to "ft_connectivityanalysis" took 0 seconds
selection fourierspctrm along dimension 2
the call to "ft_connectivityanalysis" took 0 seconds
selection crsspctrm along dimensions 1 and 2
computing pairwise non-parametric spectral factorization on 1 channel pairs
computing spectral factorization [----------------------------------------|]
the call to "ft_connectivityanalysis" took 0 seconds
the input is raw data with 2 channels and 50 trials
Warning: the data does not contain a trial definition
Warning: reconstructing sampleinfo by assuming that the trials are consecutive
segments of a continuous recording
the call to "ft_selectdata" took 0 seconds
processing trials
processing trial 50/50 nfft: 100 samples, datalength: 100 samples, 1 tapers

the call to "ft_freqanalysis" took 0 seconds
selection crsspctrm along dimensions 1 and 2
the call to "ft_connectivityanalysis" took 0 seconds
selection fourierspctrm along dimension 2
the call to "ft_connectivityanalysis" took 0 seconds
selection crsspctrm along dimensions 1 and 2
computing pairwise non-parametric spectral factorization on 1 channel pairs
computing spectral factorization [----------------------------------------|]
the call to "ft_connectivityanalysis" took 0 seconds
the input is raw data with 2 channels and 100 trials
Warning: the data does not contain a trial definition
Warning: reconstructing sampleinfo by assuming that the trials are consecutive
segments of a continuous recording
the call to "ft_selectdata" took 0 seconds
processing trials
processing trial 100/100 nfft: 100 samples, datalength: 100 samples, 1 tapers

the call to "ft_freqanalysis" took 0 seconds
selection crsspctrm along dimensions 1 and 2
```

PRO version Are you a developer? Try out the HTML to PDF API pdfcrowd.com

```
the call to "ft_connectivityanalysis" took 0 seconds
selection fourierspctrm along dimension 2
the call to "ft_connectivityanalysis" took 0 seconds
selection crsspctrm along dimensions 1 and 2
computing pairwise non-parametric spectral factorization on 1 channel pairs
computing spectral factorization [----------------------------------------|]
the call to "ft_connectivityanalysis" took 0 seconds
the input is raw data with 2 channels and 500 trials
Warning: the data does not contain a trial definition
Warning: reconstructing sampleinfo by assuming that the trials are consecutive
segments of a continuous recording
the call to "ft_selectdata" took 0 seconds
processing trials
processing trial 500/500 nfft: 100 samples, datalength: 100 samples, 1 tapers

the call to "ft_freqanalysis" took 0 seconds
selection crsspctrm along dimensions 1 and 2
the call to "ft_connectivityanalysis" took 0 seconds
selection fourierspctrm along dimension 2
the call to "ft_connectivityanalysis" took 1 seconds
selection crsspctrm along dimensions 1 and 2
computing pairwise non-parametric spectral factorization on 1 channel pairs
computing spectral factorization [----------------------------------------|]
the call to "ft_connectivityanalysis" took 0 seconds
finished simulation 1
the call to "ft_connectivitysimulation" took 0 seconds
the call to "ft_connectivitysimulation" took 0 seconds
the call to "ft_connectivitysimulation" took 0 seconds
the call to "ft_connectivitysimulation" took 0 seconds
the call to "ft_connectivitysimulation" took 0 seconds
the input is raw data with 2 channels and 5 trials
Warning: the data does not contain a trial definition
Warning: reconstructing sampleinfo by assuming that the trials are consecutive
segments of a continuous recording
the call to "ft_selectdata" took 0 seconds
processing trials
processing trial 5/5 nfft: 100 samples, datalength: 100 samples, 1 tapers

the call to "ft_freqanalysis" took 0 seconds
selection crsspctrm along dimensions 1 and 2
the call to "ft_connectivityanalysis" took 0 seconds
selection fourierspctrm along dimension 2
the call to "ft_connectivityanalysis" took 0 seconds
```

PRO version Are you a developer? Try out the HTML to PDF API pdfcrowd.com

```
selection crsspctrm along dimensions 1 and 2
computing pairwise non-parametric spectral factorization on 1 channel pairs
computing spectral factorization [----------------------------------------|]
the call to "ft_connectivityanalysis" took 0 seconds
the input is raw data with 2 channels and 10 trials
Warning: the data does not contain a trial definition
Warning: reconstructing sampleinfo by assuming that the trials are consecutive
segments of a continuous recording
the call to "ft_selectdata" took 0 seconds
processing trials
processing trial 10/10 nfft: 100 samples, datalength: 100 samples, 1 tapers

the call to "ft_freqanalysis" took 0 seconds
selection crsspctrm along dimensions 1 and 2
the call to "ft_connectivityanalysis" took 0 seconds
selection fourierspctrm along dimension 2
the call to "ft_connectivityanalysis" took 0 seconds
selection crsspctrm along dimensions 1 and 2
computing pairwise non-parametric spectral factorization on 1 channel pairs
computing spectral factorization [----------------------------------------|]
the call to "ft_connectivityanalysis" took 0 seconds
the input is raw data with 2 channels and 50 trials
Warning: the data does not contain a trial definition
Warning: reconstructing sampleinfo by assuming that the trials are consecutive
segments of a continuous recording
the call to "ft_selectdata" took 0 seconds
processing trials
processing trial 50/50 nfft: 100 samples, datalength: 100 samples, 1 tapers

the call to "ft_freqanalysis" took 0 seconds
selection crsspctrm along dimensions 1 and 2
the call to "ft_connectivityanalysis" took 0 seconds
selection fourierspctrm along dimension 2
the call to "ft_connectivityanalysis" took 0 seconds
selection crsspctrm along dimensions 1 and 2
computing pairwise non-parametric spectral factorization on 1 channel pairs
computing spectral factorization [----------------------------------------|]
the call to "ft_connectivityanalysis" took 0 seconds
the input is raw data with 2 channels and 100 trials
Warning: the data does not contain a trial definition
Warning: reconstructing sampleinfo by assuming that the trials are consecutive
segments of a continuous recording
the call to "ft_selectdata" took 0 seconds
```

PRO version Are you a developer? Try out the HTML to PDF API pdfcrowd.com

```
processing trials
processing trial 100/100 nfft: 100 samples, datalength: 100 samples, 1 tapers

the call to "ft_freqanalysis" took 0 seconds
selection crsspctrm along dimensions 1 and 2
the call to "ft_connectivityanalysis" took 0 seconds
selection fourierspctrm along dimension 2
the call to "ft_connectivityanalysis" took 0 seconds
selection crsspctrm along dimensions 1 and 2
computing pairwise non-parametric spectral factorization on 1 channel pairs
computing spectral factorization [----------------------------------------|]
the call to "ft_connectivityanalysis" took 0 seconds
the input is raw data with 2 channels and 500 trials
Warning: the data does not contain a trial definition
Warning: reconstructing sampleinfo by assuming that the trials are consecutive
segments of a continuous recording
the call to "ft_selectdata" took 0 seconds
processing trials
processing trial 500/500 nfft: 100 samples, datalength: 100 samples, 1 tapers

the call to "ft_freqanalysis" took 0 seconds
selection crsspctrm along dimensions 1 and 2
the call to "ft_connectivityanalysis" took 0 seconds
selection fourierspctrm along dimension 2
the call to "ft_connectivityanalysis" took 1 seconds
selection crsspctrm along dimensions 1 and 2
computing pairwise non-parametric spectral factorization on 1 channel pairs
computing spectral factorization [----------------------------------------|]
the call to "ft_connectivityanalysis" took 0 seconds
finished simulation 2
the call to "ft_connectivitysimulation" took 0 seconds
the call to "ft_connectivitysimulation" took 0 seconds
the call to "ft_connectivitysimulation" took 0 seconds
the call to "ft_connectivitysimulation" took 0 seconds
the call to "ft_connectivitysimulation" took 0 seconds
the input is raw data with 2 channels and 5 trials
Warning: the data does not contain a trial definition
Warning: reconstructing sampleinfo by assuming that the trials are consecutive
segments of a continuous recording
the call to "ft_selectdata" took 0 seconds
processing trials
processing trial 5/5 nfft: 100 samples, datalength: 100 samples, 1 tapers
```

PRO version Are you a developer? Try out the HTML to PDF API pdfcrowd.com

```
the call to "ft_freqanalysis" took 0 seconds
selection crsspctrm along dimensions 1 and 2
the call to "ft_connectivityanalysis" took 0 seconds
selection fourierspctrm along dimension 2
the call to "ft_connectivityanalysis" took 0 seconds
selection crsspctrm along dimensions 1 and 2
computing pairwise non-parametric spectral factorization on 1 channel pairs
computing spectral factorization [----------------------------------------|]
the call to "ft_connectivityanalysis" took 0 seconds
the input is raw data with 2 channels and 10 trials
Warning: the data does not contain a trial definition
Warning: reconstructing sampleinfo by assuming that the trials are consecutive
segments of a continuous recording
the call to "ft_selectdata" took 0 seconds
processing trials
processing trial 10/10 nfft: 100 samples, datalength: 100 samples, 1 tapers

the call to "ft_freqanalysis" took 0 seconds
selection crsspctrm along dimensions 1 and 2
the call to "ft_connectivityanalysis" took 0 seconds
selection fourierspctrm along dimension 2
the call to "ft_connectivityanalysis" took 0 seconds
selection crsspctrm along dimensions 1 and 2
computing pairwise non-parametric spectral factorization on 1 channel pairs
computing spectral factorization [----------------------------------------|]
the call to "ft_connectivityanalysis" took 0 seconds
the input is raw data with 2 channels and 50 trials
Warning: the data does not contain a trial definition
Warning: reconstructing sampleinfo by assuming that the trials are consecutive
segments of a continuous recording
the call to "ft_selectdata" took 0 seconds
processing trials
processing trial 50/50 nfft: 100 samples, datalength: 100 samples, 1 tapers

the call to "ft_freqanalysis" took 0 seconds
selection crsspctrm along dimensions 1 and 2
the call to "ft_connectivityanalysis" took 0 seconds
selection fourierspctrm along dimension 2
the call to "ft_connectivityanalysis" took 0 seconds
selection crsspctrm along dimensions 1 and 2
computing pairwise non-parametric spectral factorization on 1 channel pairs
computing spectral factorization [----------------------------------------|]
```

PRO version Are you a developer? Try out the HTML to PDF API pdfcrowd.com

```
the call to "ft_connectivityanalysis" took 0 seconds
the input is raw data with 2 channels and 100 trials
Warning: the data does not contain a trial definition
Warning: reconstructing sampleinfo by assuming that the trials are consecutive
segments of a continuous recording
the call to "ft_selectdata" took 0 seconds
processing trials
processing trial 100/100 nfft: 100 samples, datalength: 100 samples, 1 tapers

the call to "ft_freqanalysis" took 0 seconds
selection crsspctrm along dimensions 1 and 2
the call to "ft_connectivityanalysis" took 0 seconds
selection fourierspctrm along dimension 2
the call to "ft_connectivityanalysis" took 0 seconds
selection crsspctrm along dimensions 1 and 2
computing pairwise non-parametric spectral factorization on 1 channel pairs
computing spectral factorization [----------------------------------------|]
the call to "ft_connectivityanalysis" took 0 seconds
the input is raw data with 2 channels and 500 trials
Warning: the data does not contain a trial definition
Warning: reconstructing sampleinfo by assuming that the trials are consecutive
segments of a continuous recording
the call to "ft_selectdata" took 0 seconds
processing trials
processing trial 500/500 nfft: 100 samples, datalength: 100 samples, 1 tapers

the call to "ft_freqanalysis" took 0 seconds
selection crsspctrm along dimensions 1 and 2
the call to "ft_connectivityanalysis" took 0 seconds
selection fourierspctrm along dimension 2
the call to "ft_connectivityanalysis" took 1 seconds
selection crsspctrm along dimensions 1 and 2
computing pairwise non-parametric spectral factorization on 1 channel pairs
computing spectral factorization [----------------------------------------|]
the call to "ft_connectivityanalysis" took 0 seconds
finished simulation 3
the call to "ft_connectivitysimulation" took 0 seconds
the call to "ft_connectivitysimulation" took 0 seconds
the call to "ft_connectivitysimulation" took 0 seconds
the call to "ft_connectivitysimulation" took 0 seconds
the call to "ft_connectivitysimulation" took 0 seconds
the input is raw data with 2 channels and 5 trials
Warning: the data does not contain a trial definition
```

PRO version Are you a developer? Try out the HTML to PDF API pdfcrowd.com

```
Warning: reconstructing sampleinfo by assuming that the trials are consecutive
segments of a continuous recording
the call to "ft_selectdata" took 0 seconds
processing trials
processing trial 5/5 nfft: 100 samples, datalength: 100 samples, 1 tapers

the call to "ft_freqanalysis" took 0 seconds
selection crsspctrm along dimensions 1 and 2
the call to "ft_connectivityanalysis" took 0 seconds
selection fourierspctrm along dimension 2
the call to "ft_connectivityanalysis" took 0 seconds
selection crsspctrm along dimensions 1 and 2
computing pairwise non-parametric spectral factorization on 1 channel pairs
computing spectral factorization [---------------------------------------|]
the call to "ft_connectivityanalysis" took 0 seconds
the input is raw data with 2 channels and 10 trials
Warning: the data does not contain a trial definition
Warning: reconstructing sampleinfo by assuming that the trials are consecutive
segments of a continuous recording
the call to "ft_selectdata" took 0 seconds
processing trials
processing trial 10/10 nfft: 100 samples, datalength: 100 samples, 1 tapers

the call to "ft_freqanalysis" took 0 seconds
selection crsspctrm along dimensions 1 and 2
the call to "ft_connectivityanalysis" took 0 seconds
selection fourierspctrm along dimension 2
the call to "ft_connectivityanalysis" took 0 seconds
selection crsspctrm along dimensions 1 and 2
computing pairwise non-parametric spectral factorization on 1 channel pairs
computing spectral factorization [---------------------------------------|]
the call to "ft_connectivityanalysis" took 0 seconds
the input is raw data with 2 channels and 50 trials
Warning: the data does not contain a trial definition
Warning: reconstructing sampleinfo by assuming that the trials are consecutive
segments of a continuous recording
the call to "ft_selectdata" took 0 seconds
processing trials
processing trial 50/50 nfft: 100 samples, datalength: 100 samples, 1 tapers

the call to "ft_freqanalysis" took 0 seconds
selection crsspctrm along dimensions 1 and 2
the call to "ft_connectivityanalysis" took 0 seconds
```

PRO version Are you a developer? Try out the HTML to PDF API pdfcrowd.com

```
selection fourierspctrm along dimension 2
the call to "ft_connectivityanalysis" took 0 seconds
selection crsspctrm along dimensions 1 and 2
computing pairwise non-parametric spectral factorization on 1 channel pairs
computing spectral factorization [----------------------------------------|]
the call to "ft_connectivityanalysis" took 0 seconds
the input is raw data with 2 channels and 100 trials
Warning: the data does not contain a trial definition
Warning: reconstructing sampleinfo by assuming that the trials are consecutive
segments of a continuous recording
the call to "ft_selectdata" took 0 seconds
processing trials
processing trial 100/100 nfft: 100 samples, datalength: 100 samples, 1 tapers

the call to "ft_freqanalysis" took 0 seconds
selection crsspctrm along dimensions 1 and 2
the call to "ft_connectivityanalysis" took 0 seconds
selection fourierspctrm along dimension 2
the call to "ft_connectivityanalysis" took 0 seconds
selection crsspctrm along dimensions 1 and 2
computing pairwise non-parametric spectral factorization on 1 channel pairs
computing spectral factorization [----------------------------------------|]
the call to "ft_connectivityanalysis" took 0 seconds
the input is raw data with 2 channels and 500 trials
Warning: the data does not contain a trial definition
Warning: reconstructing sampleinfo by assuming that the trials are consecutive
segments of a continuous recording
the call to "ft_selectdata" took 0 seconds
processing trials
processing trial 500/500 nfft: 100 samples, datalength: 100 samples, 1 tapers

the call to "ft_freqanalysis" took 0 seconds
selection crsspctrm along dimensions 1 and 2
the call to "ft_connectivityanalysis" took 0 seconds
selection fourierspctrm along dimension 2
the call to "ft_connectivityanalysis" took 1 seconds
selection crsspctrm along dimensions 1 and 2
computing pairwise non-parametric spectral factorization on 1 channel pairs
computing spectral factorization [----------------------------------------|]
the call to "ft_connectivityanalysis" took 0 seconds
finished simulation 4
the call to "ft_connectivitysimulation" took 0 seconds
```

PRO version Are you a developer? Try out the HTML to PDF API pdfcrowd.com

```
the call to "ft_connectivitysimulation" took 0 seconds
the call to "ft_connectivitysimulation" took 0 seconds
the call to "ft_connectivitysimulation" took 0 seconds
the call to "ft_connectivitysimulation" took 0 seconds
the input is raw data with 2 channels and 5 trials
Warning: the data does not contain a trial definition
Warning: reconstructing sampleinfo by assuming that the trials are consecutive
segments of a continuous recording
the call to "ft_selectdata" took 0 seconds
processing trials
processing trial 5/5 nfft: 100 samples, datalength: 100 samples, 1 tapers

the call to "ft_freqanalysis" took 0 seconds
selection crsspctrm along dimensions 1 and 2
the call to "ft_connectivityanalysis" took 0 seconds
selection fourierspctrm along dimension 2
the call to "ft_connectivityanalysis" took 0 seconds
selection crsspctrm along dimensions 1 and 2
computing pairwise non-parametric spectral factorization on 1 channel pairs
computing spectral factorization [----------------------------------------|]
the call to "ft_connectivityanalysis" took 0 seconds
the input is raw data with 2 channels and 10 trials
Warning: the data does not contain a trial definition
Warning: reconstructing sampleinfo by assuming that the trials are consecutive
segments of a continuous recording
the call to "ft_selectdata" took 0 seconds
processing trials
processing trial 10/10 nfft: 100 samples, datalength: 100 samples, 1 tapers

the call to "ft_freqanalysis" took 0 seconds
selection crsspctrm along dimensions 1 and 2
the call to "ft_connectivityanalysis" took 0 seconds
selection fourierspctrm along dimension 2
the call to "ft_connectivityanalysis" took 0 seconds
selection crsspctrm along dimensions 1 and 2
computing pairwise non-parametric spectral factorization on 1 channel pairs
computing spectral factorization [----------------------------------------|]
the call to "ft_connectivityanalysis" took 0 seconds
the input is raw data with 2 channels and 50 trials
Warning: the data does not contain a trial definition
Warning: reconstructing sampleinfo by assuming that the trials are consecutive
segments of a continuous recording
the call to "ft_selectdata" took 0 seconds
```

PRO version Are you a developer? Try out the HTML to PDF API pdfcrowd.com

```
processing trials
processing trial 50/50 nfft: 100 samples, datalength: 100 samples, 1 tapers

the call to "ft_freqanalysis" took 0 seconds
selection crsspctrm along dimensions 1 and 2
the call to "ft_connectivityanalysis" took 0 seconds
selection fourierspctrm along dimension 2
the call to "ft_connectivityanalysis" took 0 seconds
selection crsspctrm along dimensions 1 and 2
computing pairwise non-parametric spectral factorization on 1 channel pairs
computing spectral factorization [----------------------------------------|]
the call to "ft_connectivityanalysis" took 0 seconds
the input is raw data with 2 channels and 100 trials
Warning: the data does not contain a trial definition
Warning: reconstructing sampleinfo by assuming that the trials are consecutive
segments of a continuous recording
the call to "ft_selectdata" took 0 seconds
processing trials
processing trial 100/100 nfft: 100 samples, datalength: 100 samples, 1 tapers

the call to "ft_freqanalysis" took 0 seconds
selection crsspctrm along dimensions 1 and 2
the call to "ft_connectivityanalysis" took 0 seconds
selection fourierspctrm along dimension 2
the call to "ft_connectivityanalysis" took 0 seconds
selection crsspctrm along dimensions 1 and 2
computing pairwise non-parametric spectral factorization on 1 channel pairs
computing spectral factorization [----------------------------------------|]
the call to "ft_connectivityanalysis" took 0 seconds
the input is raw data with 2 channels and 500 trials
Warning: the data does not contain a trial definition
Warning: reconstructing sampleinfo by assuming that the trials are consecutive
segments of a continuous recording
the call to "ft_selectdata" took 0 seconds
processing trials
processing trial 500/500 nfft: 100 samples, datalength: 100 samples, 1 tapers

the call to "ft_freqanalysis" took 0 seconds
selection crsspctrm along dimensions 1 and 2
the call to "ft_connectivityanalysis" took 0 seconds
selection fourierspctrm along dimension 2
the call to "ft_connectivityanalysis" took 1 seconds
selection crsspctrm along dimensions 1 and 2
```

PRO version Are you a developer? Try out the HTML to PDF API pdfcrowd.com

```
computing pairwise non-parametric spectral factorization on 1 channel pairs
computing spectral factorization [--------------------------------------|]
the call to "ft_connectivityanalysis" took 0 seconds
finished simulation 5
```

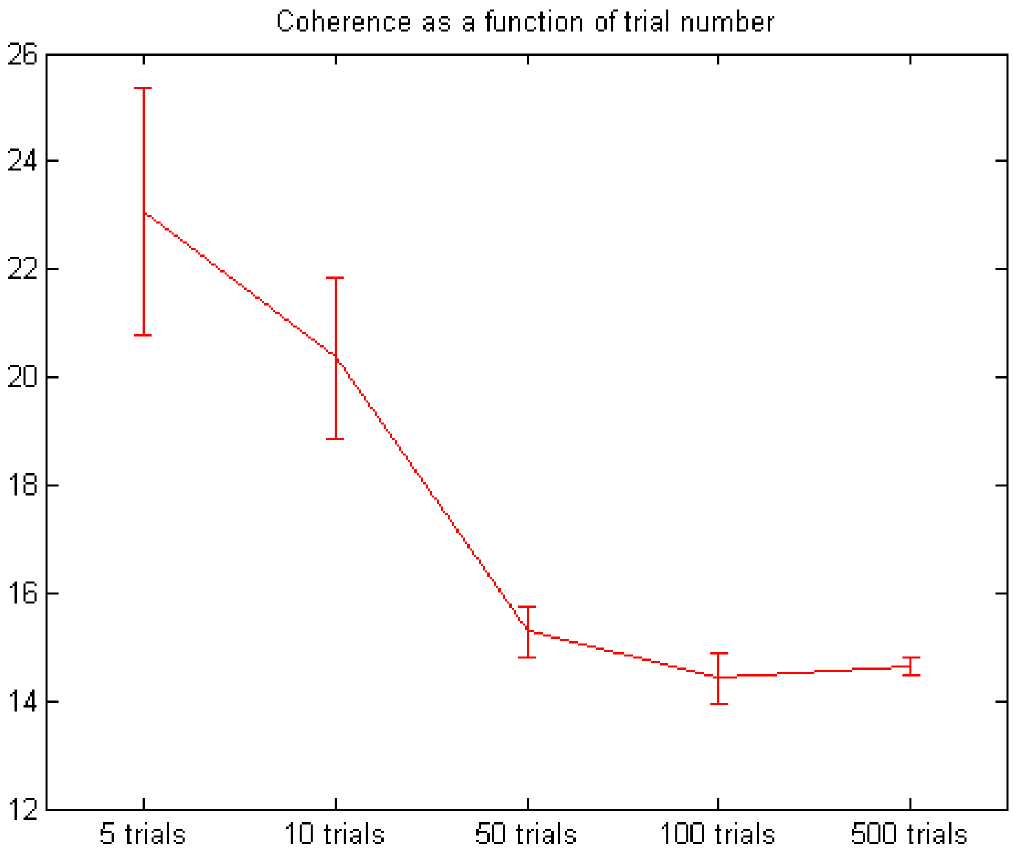

PRO version Are you a developer? Try out the HTML to PDF API pdfcrowd.com

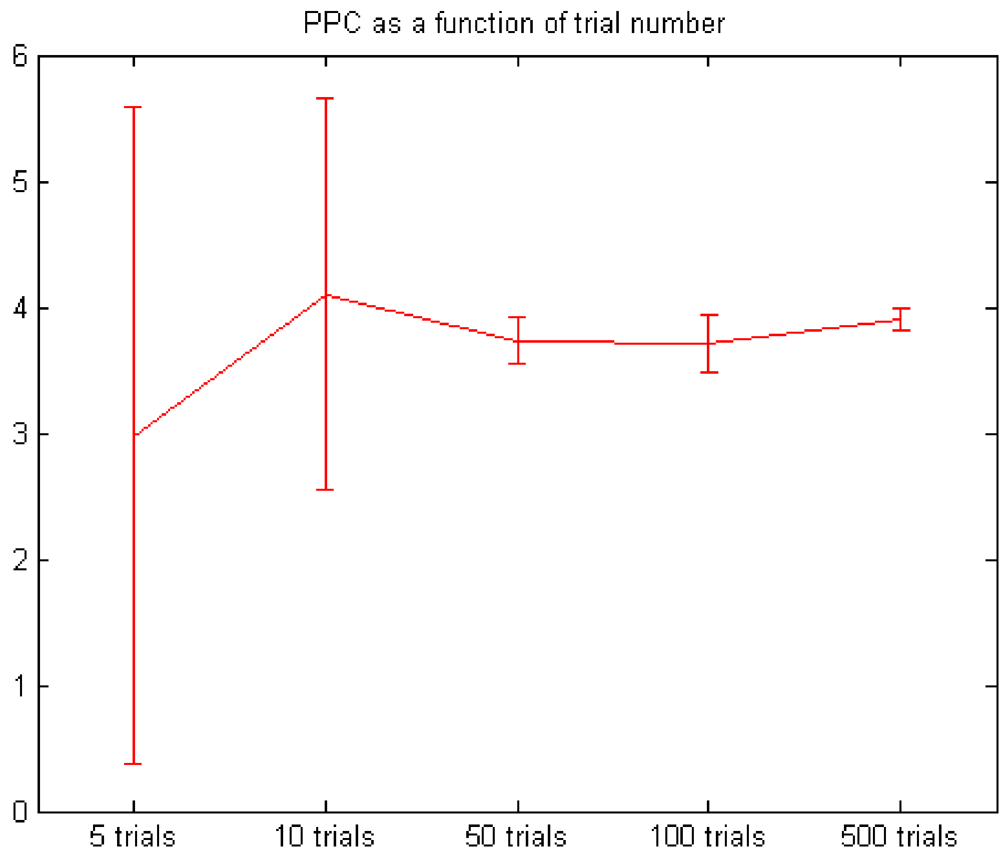

PRO version Are you a developer? Try out the HTML to PDF API pdfcrowd.com

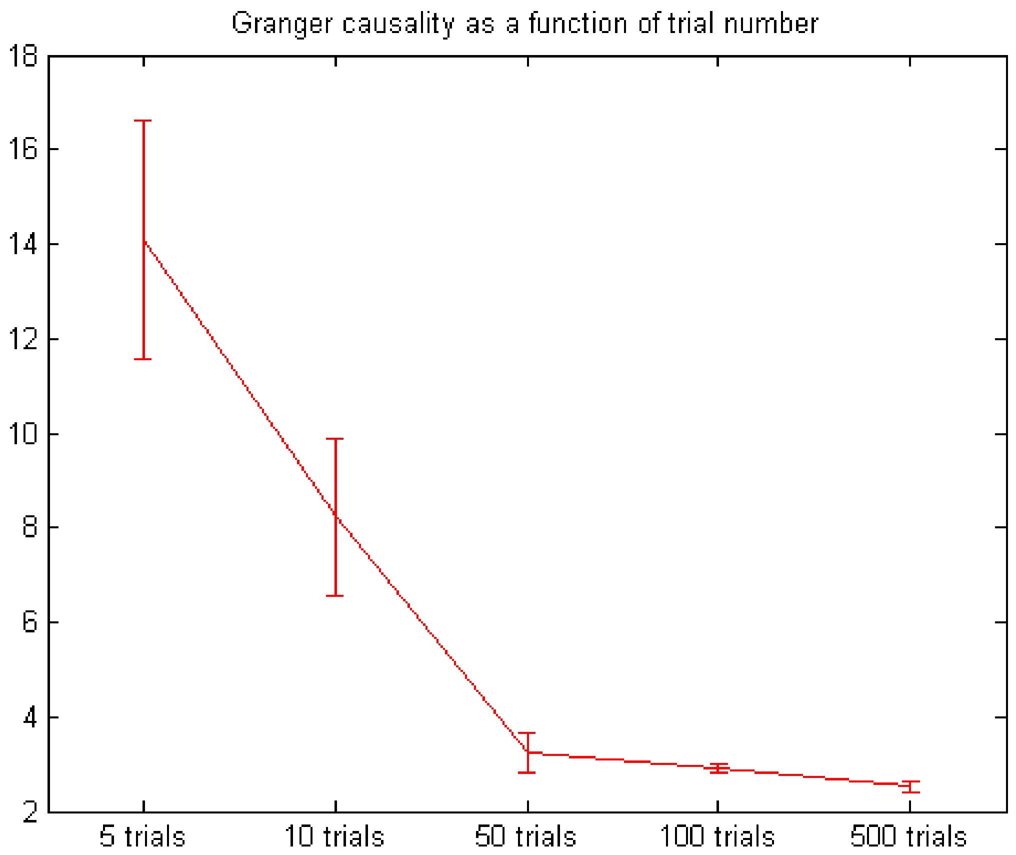

Published with MATLAB® R2013a

PRO version Are you a developer? Try out the HTML to PDF API pdfcrowd.com
