# Supplementary material for: A Tutorial Review of Functional Connectivity Analysis Methods and Their Interpretational Pitfalls
Source: Front Syst Neurosci. 2016 Jan 8;9:175. doi: 10.3389/fnsys.2015.00175 (PMC4705224; doi:10.3389/fnsys.2015.00175)
Supplement: Supplementary file 5 [file sim_signaltonoise.pdf]

```
%simulate two signals that both influence each other with equal weight at lag = 1 and 2 (Figure 7,
Case 1: no noise)
cfg = [];
cfg.method      = 'ar';
cfg.ntrials     = 1000;
cfg.triallength = 0.5;
cfg.fsample     = 200;
cfg.nsignal     = 2;
cfg.bpfilter    = 'no';
cfg.blc         = 'yes';
%auto-regressive parameters for lag 1
cfg.params(:,:,1)      = [0.55   0.025;
                          0.025  0.55];
%auto-regressive parameters for lag 2
cfg.params(:,:,2)      = [-0.8  -0.1;
                          -0.1  -0.8];
cfg.noisecov     = [1 0.3;
                    0.3 1];
%in this case, no added noise
cfg.absnoise     =         [0 0];
data = ft_connectivitysimulation(cfg);

%(Figure 7, Case 2: extra noise)
cfg.absnoise = [1 0]; %increase the amount of independent noise added on top of signal 1

datan = ft_connectivitysimulation(cfg);


%%%%%%%%%%%%%%%%%%%%%%%%%%%%%%%%%%%%%%%%%%%%%%%%%%%%%%%%%%%%%%%%%%%%%%%%%%%%%
%Calculate power, coherence, and Granger causality based on parametric and
%non-parametric estimates for "clean" data without extra noise (Case 1, Figure 7B-D)

%Parametric (auto-regressive model based) derivation of AR coefficients
%multivariate analysis will compute the auto-regressive coefficients and associated noise covariance
matrix

cfg           = [];
cfg.order     = 2;
mdata         = ft_mvaranalysis(cfg, data);

cfg        = [];
cfg.method = 'mvar';
cfg.foi    = [0:120];
mfreq      = ft_freqanalysis(cfg, mdata);

%calculate the fourier coefficients (non-parametric derivation of power)
cfg        = [];
cfg.method = 'mtmfft';
cfg.taper  = 'dpss';
cfg.output = 'fourier';
cfg.tapsmofrq = 4;
cfg.foilim = [0 100];
freq       = ft_freqanalysis(cfg, data);

cfg           = [];
cfg.method     = 'coh';
cfg.complex    = 'abs';
coh1 = ft_connectivityanalysis(cfg, freq);
coh1_nonoise = coh1;
```

```
csd = ft_checkdata(freq, 'cmbrepresentation', 'fullfast');

cfg           = [];
cfg.method = 'coh';
cohp = ft_connectivityanalysis(cfg, mfreq);

cfg           = [];
cfg.method    = 'granger';
cfg.granger.sfmethod = 'bivariate';
g1 = ft_connectivityanalysis(cfg, csd);

g1_nonoise = g1;

cfg           = [];
cfg.method    = 'granger';
gp = ft_connectivityanalysis(cfg, mfreq);


figure;
plot(freq.freq,squeeze(csd.crsspctrm(1,1,:))); hold on;
plot(freq.freq,squeeze(csd.crsspctrm(2,2,:)),'r');
title('Non-parametric power estimates, , case 1 (no extra noise)'); legend('chan 1','chan 2');

figure; plot(coh1.freq,squeeze(coh1.cohspctrm(1,2,:))); title('Nonparametric Coherence spectrum,
case 1 (no extra noise)');
figure; plot(cohp.freq,squeeze(cohp.cohspctrm(1,2,:))); title('Parametric Coherence spectrum, case 1
(no extra noise)');

figure;plot(g1.freq,squeeze(g1.grangerspctrm(1,:)));hold on
plot(g1.freq,squeeze(g1.grangerspctrm(2,:)),'r');
title('Granger nonparametric estimates, case 1 (no extra noise)');legend('1->2','2->1');

figure;plot(gp.freq,squeeze(gp.grangerspctrm(1,2,:)));hold on
plot(gp.freq,squeeze(gp.grangerspctrm(2,1,:)),'r');
title('Granger parametric estimates, case 1 (no extra noise)');legend('1->2','2->1');


%%%%%%%%%%%%%%%%%%%%%%%%%%%%%%%%%%%%%%%%%%%%%%%%%%%%%%%%%%%%%%%%%%%%%%%%%%%%%
%Calculate power, coherence, and Granger causality based on parametric and
%non-parametric estimates for data with extra noise (Case 2, Figure 7C - blue trace, 7D and E)

%Parametric (auto-regressive model based) derivation of AR coefficients
%multivariate analysis will compute the auto-regressive coefficients and associated noise covariance
matrix

cfg           = [];
cfg.order     = 2;
mdata         = ft_mvaranalysis(cfg, datan);

mdata.offset = 0;
cfg        = [];
cfg.method = 'mvar';
cfg.foi    = [0:120];
mfreq      = ft_freqanalysis(cfg, mdata);

%calculate the fourier coefficients (non-parametric derivation of power)
cfg        = [];
cfg.method = 'mtmfft';
cfg.taper  = 'dpss';
```

```
cfg.output = 'fourier';
cfg.tapsmofrq = 4;
cfg.foilim = [0 100];
freq       = ft_freqanalysis(cfg, datan);

cfg           = [];
cfg.method    = 'coh';
cfg.complex   = 'abs';
coh1 = ft_connectivityanalysis(cfg, freq);

csd = ft_checkdata(freq, 'cmbrepresentation', 'fullfast');

cfg           = [];
cfg.method = 'coh';
cohp = ft_connectivityanalysis(cfg, mfreq);

cfg           = [];
cfg.method    = 'granger';
cfg.granger.sfmethod = 'bivariate';
g1 = ft_connectivityanalysis(cfg, csd);

cfg           = [];
cfg.method    = 'granger';
gp = ft_connectivityanalysis(cfg, mfreq);


figure;
plot(freq.freq,squeeze(csd.crsspctrm(1,1,:))); hold on;
plot(freq.freq,squeeze(csd.crsspctrm(2,2,:)),'r');
title('Non-parametric power estimates, case 2 (extra noise on channel 1)'); legend('chan 1','chan
2');

figure; plot(coh1.freq,squeeze(coh1.cohspctrm(1,2,:))); title('Nonparametric Coherence spectrum,
case 2 (extra noise on channel 1)');
figure; plot(cohp.freq,squeeze(cohp.cohspctrm(1,2,:))); title('Parametric Coherence spectrum, case 2
(extra noise on channel 1)');

figure;plot(g1.freq,squeeze(g1.grangerspctrm(1,:)));hold on
plot(g1.freq,squeeze(g1.grangerspctrm(2,:)),'r');
title('Granger nonparametric estimates, case 2 (extra noise on channel 1)');legend('1->2','2->1');

figure;plot(gp.freq,squeeze(gp.grangerspctrm(1,2,:)));hold on
plot(gp.freq,squeeze(gp.grangerspctrm(2,1,:)),'r');
title('Granger parametric estimates, case 2 (extra noise on channel 1)');legend('1->2','2->1');


%%%%%%%%%%%%%%%%%%%%%%%%%%%%%%%%%%%%%%%%%%%%%%%%%%%%%%%%%%%%%%%%%%%%%%%%%%%%%%
%time-reversed testing for this WEAK asymmetry (asymmetry in GC caused only by SNR) - Figure 7G

csd_tr = csd;
%time-reverse the cross-spectral density matrix, this is accomplished by taking the complex
%conjugate of the CSD. Note that this manipulation does not affect the real components of the CSD
%(e.g., the diagonal elements which are by definition only real-valued)
csd_tr.crsspctrm = conj(csd.crsspctrm);

cfg           = [];
cfg.method = 'coh';
coh_reversed = ft_connectivityanalysis(cfg, csd_tr);

cfg           = [];
cfg.method    = 'granger';
```

```
cfg.granger.sfmethod = 'bivariate';
g_reversed = ft_connectivityanalysis(cfg, csd_tr);

figure;
plot(csd_tr.freq,squeeze(csd_tr.crsspctrm(1,1,:))); hold on;
plot(csd_tr.freq,squeeze(csd_tr.crsspctrm(2,2,:)),'r');
title('Non-parametric power estimates, , case 1 (no extra noise), time-reversed'); legend('chan
1','chan 2');

figure; plot(coh_reversed.freq,squeeze(coh_reversed.cohspctrm(1,2,:))); title('Nonparametric
Coherence spectrum, case 2 (extra noise on channel 1), time-reversed');

figure;plot(g_reversed.freq,squeeze(g_reversed.grangerspctrm(1,:)));hold on
plot(g_reversed.freq,squeeze(g_reversed.grangerspctrm(2,:)),'r');
title('Granger nonparametric estimates, case 2 (extra noise on channel 1), time-
reversed');legend('1->2','2->1');


%%%%%%%%%%%%%%%%%%%%%%%%%%%%%%%%%%%%%%%%%%%%%%%%%%%%%%%%%%%%%%%%%%%%%%%%%%%%%%%%%
%Simulate STRONG asymmetry by including only a unidirectional flow from 2 to 1 as in Figure 8

cfg = [];
cfg.method      = 'ar';
cfg.ntrials     = 1000;
cfg.triallength = 0.5;
cfg.fsample     = 200;
cfg.nsignal     = 2;
cfg.bpfilter    = 'no';
cfg.blc         = 'yes';
cfg.params(:,:,1)      = [0.55   0.025;
                          0  0.55];
cfg.params(:,:,2)      = [-0.8  -0.1;
                          0  -0.8];
cfg.noisecov     = [1 0.3;
                    0.3 1];
cfg.absnoise     =         [0 0];
data = ft_connectivitysimulation(cfg);


cfg        = [];
cfg.method = 'mtmfft';
cfg.taper  = 'dpss';
cfg.output = 'fourier';
cfg.tapsmofrq = 4;
cfg.foilim = [0 100];
freq       = ft_freqanalysis(cfg, data);


cfg           = [];
cfg.method    = 'coh';
cfg.complex   = 'abs';
coh1 = ft_connectivityanalysis(cfg, freq);

csd = ft_checkdata(freq, 'cmbrepresentation', 'fullfast');

%Estimate Granger causality in the forward time direction - Figure 8D
cfg           = [];
cfg.method    = 'granger';
cfg.granger.sfmethod = 'bivariate';
g1 = ft_connectivityanalysis(cfg, csd);
```

```
figure;
plot(freq.freq,squeeze(csd.crsspctrm(1,1,:))); hold on;
plot(freq.freq,squeeze(csd.crsspctrm(2,2,:)),'r');
title('Non-parametric power estimates'); legend('chan 1','chan 2');

figure; plot(coh1.freq,squeeze(coh1.cohspctrm(1,2,:))); title('Nonparametric Coherence spectrum,
case 1 (no extra noise)');

figure;plot(g1.freq,squeeze(g1.grangerspctrm(1,:)));hold on
plot(g1.freq,squeeze(g1.grangerspctrm(2,:)),'r');
title('Granger nonparametric estimates');legend('1->2','2->1');



%%%%%%%%%%%%%%%%%%%%%%%%%%%%%%%%%%%%%%%%%%%%%%%%%%%%%%%%%%%%%%%%%%%%%%%%%%%%%%
%Time-reverse the CSD by taking the complex conjugate
csd_tr = csd;
csd_tr.crsspctrm = conj(csd.crsspctrm);

%Estimate Granger causality in the reversed time direction - Figure 8E
cfg           = [];
cfg.method    = 'granger';
cfg.granger.sfmethod = 'bivariate';
g2 = ft_connectivityanalysis(cfg, csd_tr);

figure;plot(g2.freq,squeeze(g2.grangerspctrm(1,:)));hold on
plot(g2.freq,squeeze(g2.grangerspctrm(2,:)),'r');
title('Granger nonparametric estimates, time-reversed');legend('1->2','2->1');
```

```
the call to "ft_connectivitysimulation" took 2 seconds
the call to "ft_connectivitysimulation" took 1 seconds
Warning: the data does not contain a trial definition
Warning: reconstructing sampleinfo by assuming that the trials are consecutive
segments of a continuous recording
the call to "ft_selectdata" took 0 seconds
preprocessing
preprocessing trial 1000 from 1000

the call to "ft_preprocessing" took 1 seconds
the call to "ft_redefinetrial" took 0 seconds
computing AR-model [------------------------------------------------------------|]
the call to "ft_mvaranalysis" took 3 seconds
the input is mvar data
Warning: could not determine dimord of "dof" in the following data
         dimord: 'chan_chan_lag'
          label: {2x1 cell}
         coeffs: [2x2x2 double]
       noisecov: [2x2 double]
            dof: 1000
    fsampleorig: 200
            cfg: [1x1 struct]

not including "dof" in selection
the call to "ft_selectdata" took 0 seconds
computing MAR-model based TFR
processing timewindow 1 from 1
```

```
the call to "ft_freqanalysis_mvar" took 0 seconds
the input is raw data with 2 channels and 1000 trials
Warning: the data does not contain a trial definition
Warning: reconstructing sampleinfo by assuming that the trials are consecutive
segments of a continuous recording
the call to "ft_selectdata" took 0 seconds
processing trials
processing trial 1000/1000 nfft: 100 samples, datalength: 100 samples, 3 tapers

the call to "ft_freqanalysis" took 2 seconds
selection fourierspctrm along dimension 2
averaging crsspctrm over rpt
removing dimension rpt from crsspctrm
the call to "ft_connectivityanalysis" took 4 seconds
selection crsspctrm along dimensions 1 and 2
selection transfer along dimensions 1 and 2
the call to "ft_connectivityanalysis" took 0 seconds
selection crsspctrm along dimensions 1 and 2
computing pairwise non-parametric spectral factorization on 1 channel pairs
computing spectral factorization [---------------------------------------/]
the call to "ft_connectivityanalysis" took 1 seconds
selection crsspctrm along dimensions 1 and 2
selection transfer along dimensions 1 and 2
the call to "ft_connectivityanalysis" took 0 seconds
Warning: the data does not contain a trial definition
Warning: reconstructing sampleinfo by assuming that the trials are consecutive
segments of a continuous recording
the call to "ft_selectdata" took 0 seconds
preprocessing
preprocessing trial 1000 from 1000

the call to "ft_preprocessing" took 1 seconds
the call to "ft_redefinetrial" took 0 seconds
computing AR-model [--------------------------------------------------------|]
the call to "ft_mvaranalysis" took 1 seconds
the input is mvar data
Warning: could not determine dimord of "dof" in the following data
         dimord: 'chan_chan_lag'
          label: {2x1 cell}
         coeffs: [2x2x2 double]
       noisecov: [2x2 double]
            dof: 1000
    fsampleorig: 200
            cfg: [1x1 struct]
         offset: 0

Warning: could not determine dimord of "offset" in the following data
         dimord: 'chan_chan_lag'
          label: {2x1 cell}
         coeffs: [2x2x2 double]
       noisecov: [2x2 double]
            dof: 1000
    fsampleorig: 200
            cfg: [1x1 struct]
         offset: 0

not including "dof" in selection
not including "offset" in selection
the call to "ft_selectdata" took 0 seconds
computing MAR-model based TFR
processing timewindow 1 from 1
```

```
the call to "ft_freqanalysis_mvar" took 0 seconds
the input is raw data with 2 channels and 1000 trials
Warning: the data does not contain a trial definition
Warning: reconstructing sampleinfo by assuming that the trials are consecutive
segments of a continuous recording
the call to "ft_selectdata" took 0 seconds
processing trials
processing trial 1000/1000 nfft: 100 samples, datalength: 100 samples, 3 tapers

the call to "ft_freqanalysis" took 1 seconds
selection fourierspctrm along dimension 2
averaging crsspctrm over rpt
removing dimension rpt from crsspctrm
the call to "ft_connectivityanalysis" took 3 seconds
selection crsspctrm along dimensions 1 and 2
selection transfer along dimensions 1 and 2
the call to "ft_connectivityanalysis" took 0 seconds
selection crsspctrm along dimensions 1 and 2
computing pairwise non-parametric spectral factorization on 1 channel pairs
computing spectral factorization [---------------------------------------|]
the call to "ft_connectivityanalysis" took 0 seconds
selection crsspctrm along dimensions 1 and 2
selection transfer along dimensions 1 and 2
the call to "ft_connectivityanalysis" took 0 seconds
selection crsspctrm along dimensions 1 and 2
the call to "ft_connectivityanalysis" took 0 seconds
selection crsspctrm along dimensions 1 and 2
computing pairwise non-parametric spectral factorization on 1 channel pairs
computing spectral factorization [---------------------------------------|]
the call to "ft_connectivityanalysis" took 0 seconds
the call to "ft_connectivitysimulation" took 1 seconds
the input is raw data with 2 channels and 1000 trials
Warning: the data does not contain a trial definition
Warning: reconstructing sampleinfo by assuming that the trials are consecutive
segments of a continuous recording
the call to "ft_selectdata" took 0 seconds
processing trials
processing trial 1000/1000 nfft: 100 samples, datalength: 100 samples, 3 tapers

the call to "ft_freqanalysis" took 1 seconds
selection fourierspctrm along dimension 2
averaging crsspctrm over rpt
removing dimension rpt from crsspctrm
the call to "ft_connectivityanalysis" took 3 seconds
selection crsspctrm along dimensions 1 and 2
computing pairwise non-parametric spectral factorization on 1 channel pairs
computing spectral factorization [---------------------------------------|]
the call to "ft_connectivityanalysis" took 0 seconds
selection crsspctrm along dimensions 1 and 2
computing pairwise non-parametric spectral factorization on 1 channel pairs
computing spectral factorization [---------------------------------------|]
the call to "ft_connectivityanalysis" took 0 seconds
```

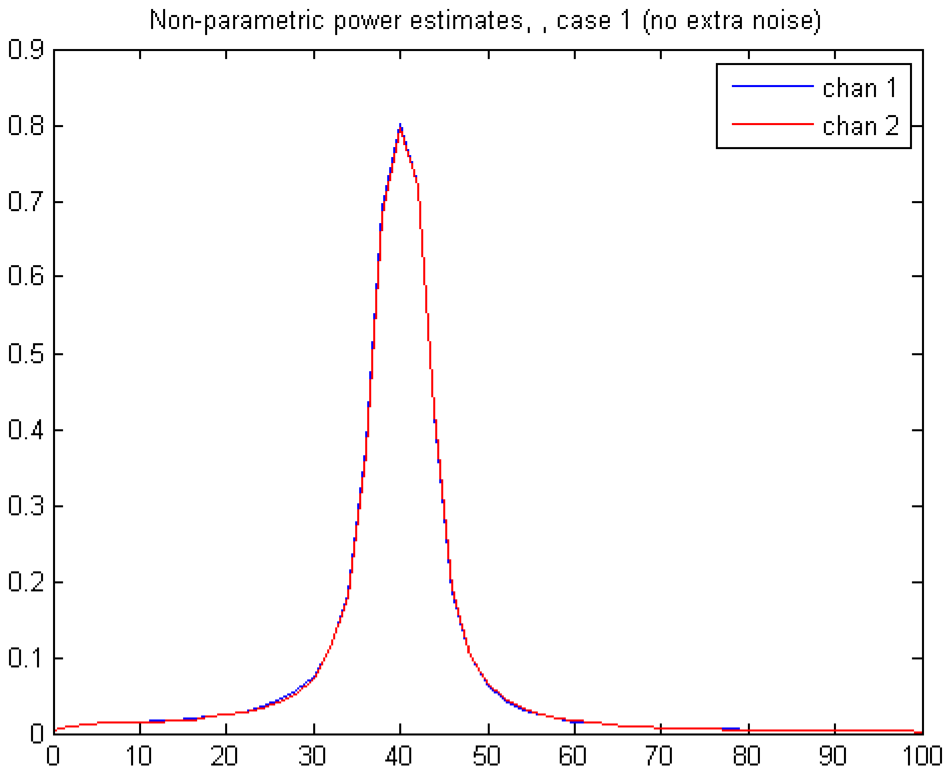
Non-parametric power estimates, , case 1 (no extra noise)
chan 1
chan 2
0.9
0.8
0.7
0.6
0.5
0.4
0.3
0.2
0.1
0
0
10
20
30
40
50
60
70
80
90
100

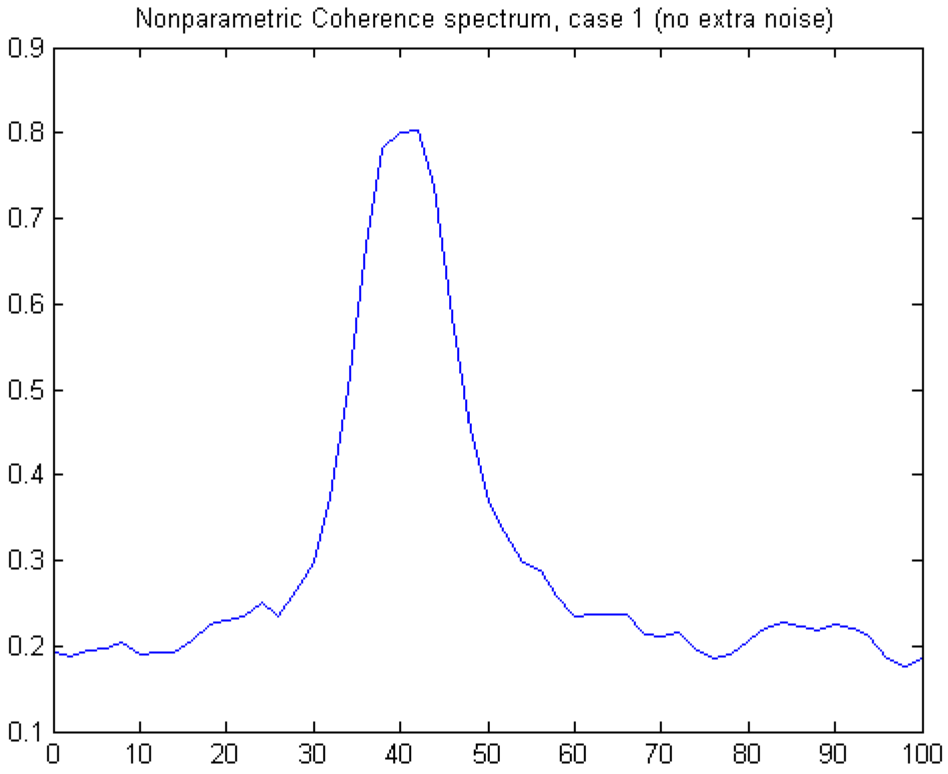
Nonparametric Coherence spectrum, case 1 (no extra noise)
0.9
0.8
0.7
0.6
0.5
0.4
0.3
0.2
0.1
0
10
20
30
40
50
60
70
80
90
100

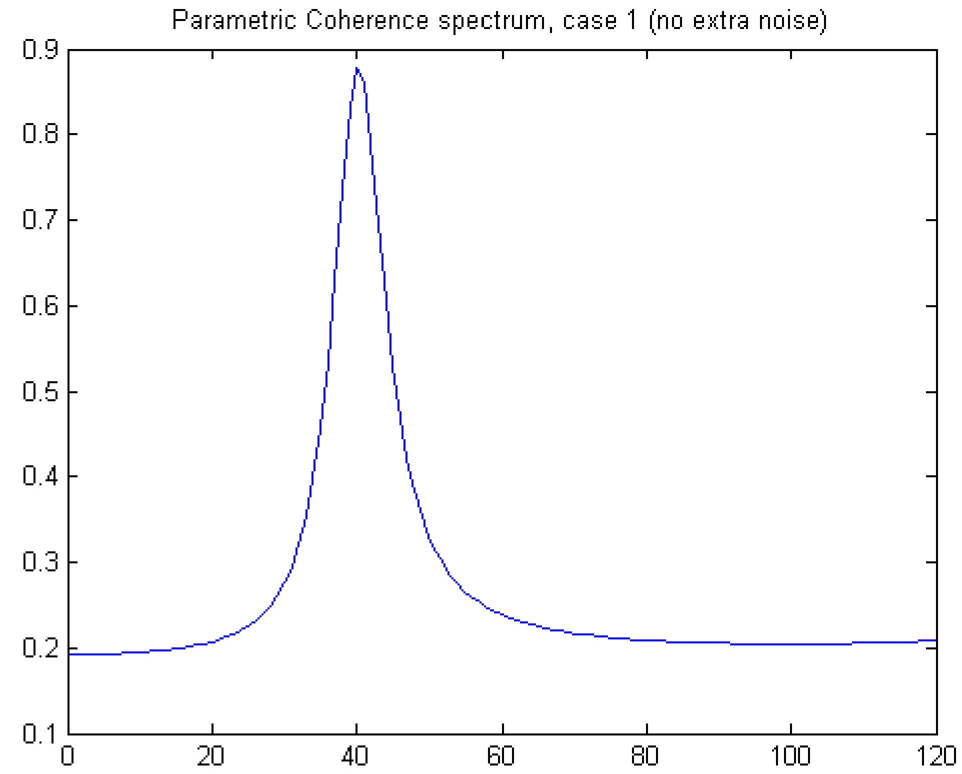
Parametric Coherence spectrum, case 1 (no extra noise)
0.9
0.8
0.7
0.6
0.5
0.4
0.3
0.2
0.1
0
20
40
60
80
100
120

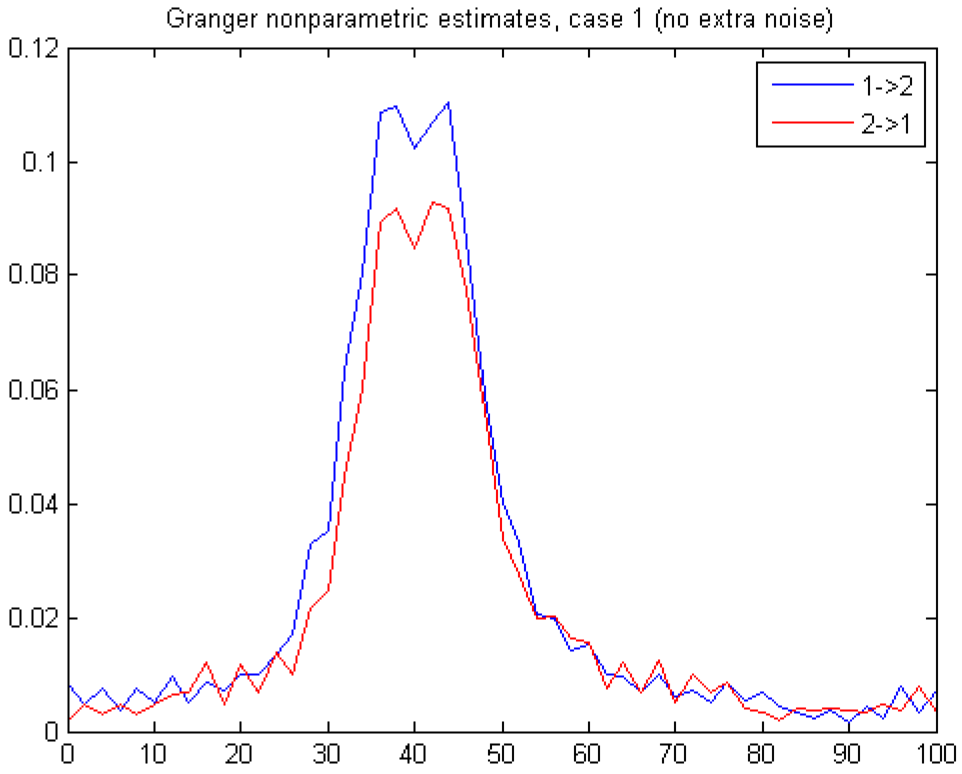
Granger nonparametric estimates, case 1 (no extra noise)
1->2
2->1
0.12
0.1
0.08
0.06
0.04
0.02
0
0
10
20
30
40
50
60
70
80
90
100

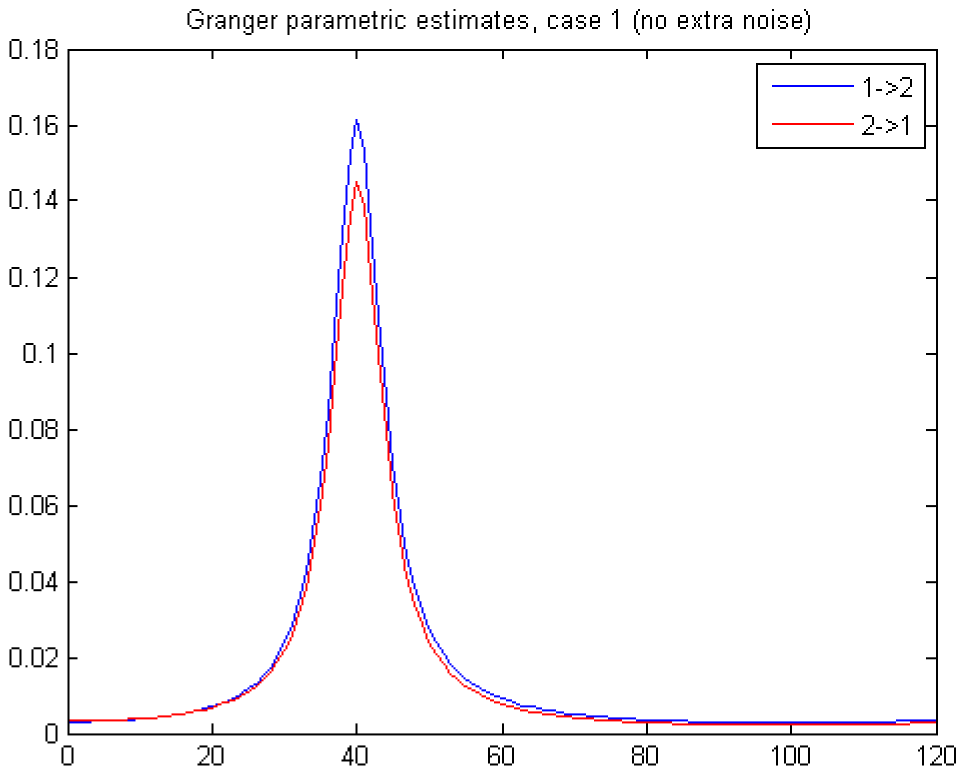
Granger parametric estimates, case 1 (no extra noise)
1->2
2->1
0.18
0.16
0.14
0.12
0.1
0.08
0.06
0.04
0.02
0
0
20
40
60
80
100
120

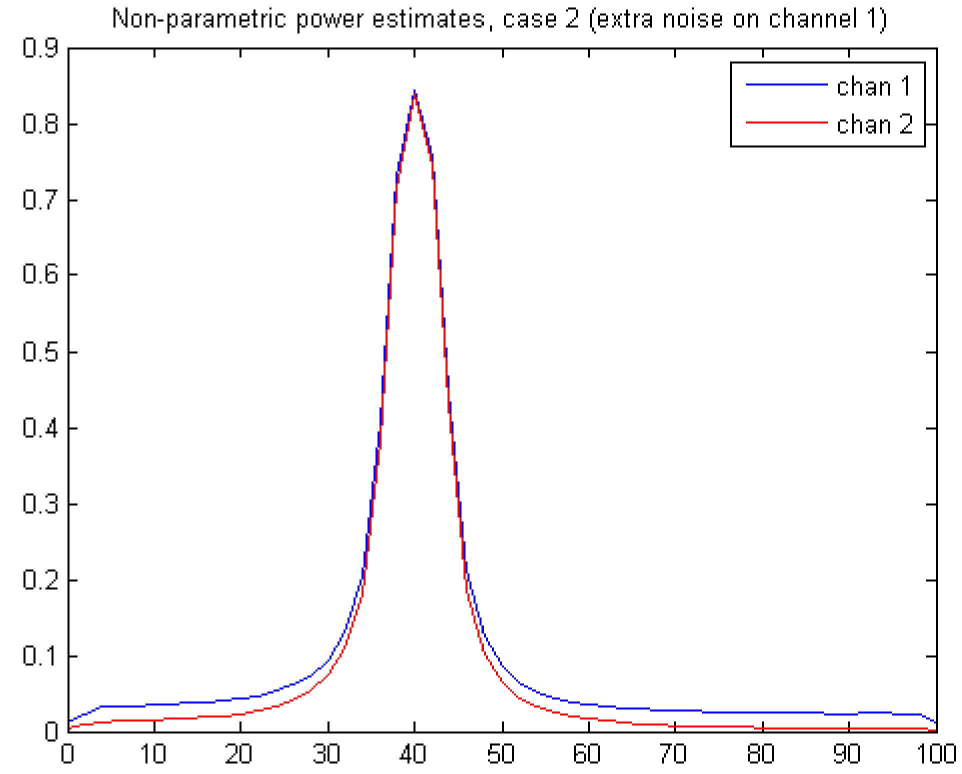
Non-parametric power estimates, case 2 (extra noise on channel 1)
chan 1
chan 2
0.9
0.8
0.7
0.6
0.5
0.4
0.3
0.2
0.1
0
0
10
20
30
40
50
60
70
80
90
100

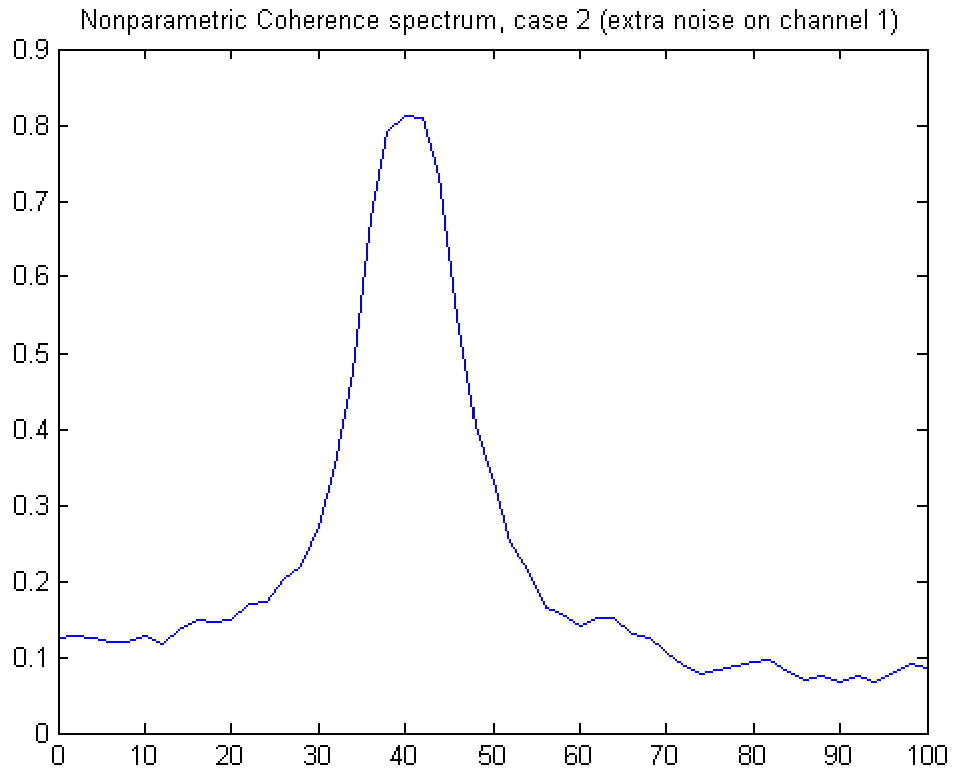
Nonparametric Coherence spectrum, case 2 (extra noise on channel 1)
0.9
0.8
0.7
0.6
0.5
0.4
0.3
0.2
0.1
0
0
10
20
30
40
50
60
70
80
90
100

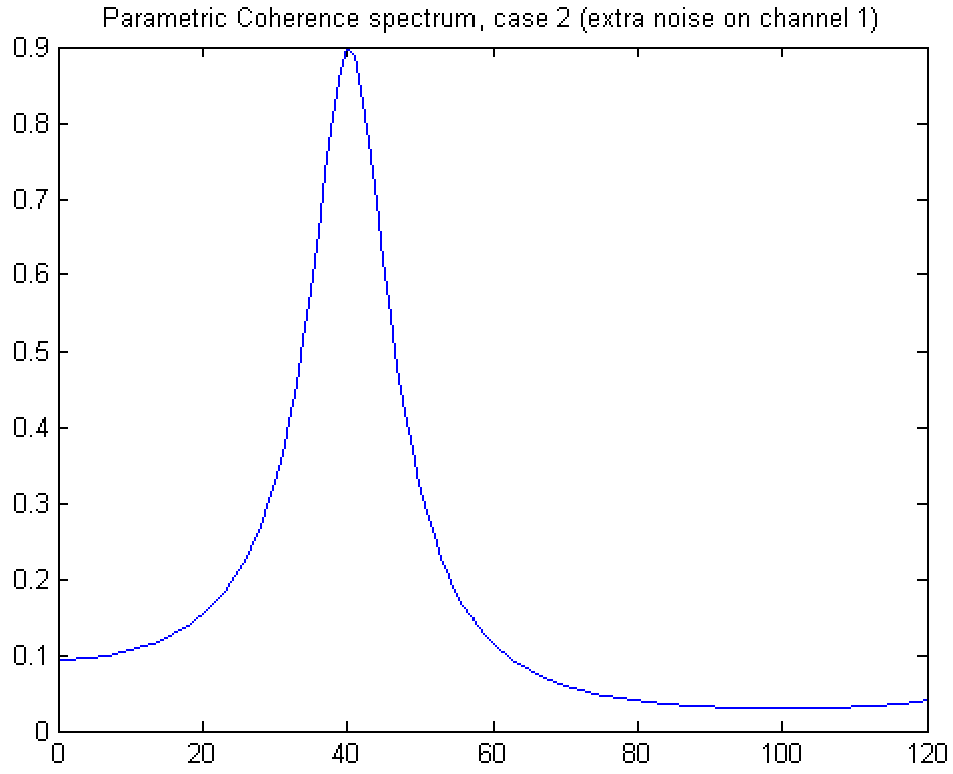
Parametric Coherence spectrum, case 2 (extra noise on channel 1)
0.9
0.8
0.7
0.6
0.5
0.4
0.3
0.2
0.1
0
0
20
40
60
80
100
120

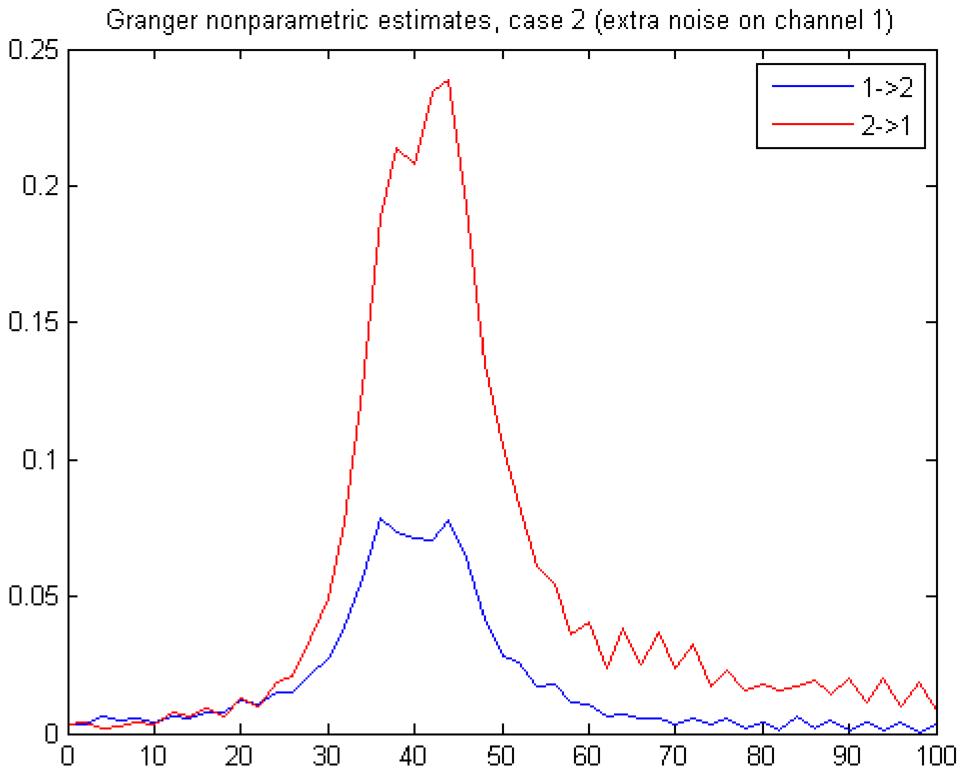

Granger nonparametric estimates, case 2 (extra noise on channel 1)
1->2
2->1
0.25
0.2
0.15
0.1
0.05
0
0
10
20
30
40
50
60
70
80
90
100

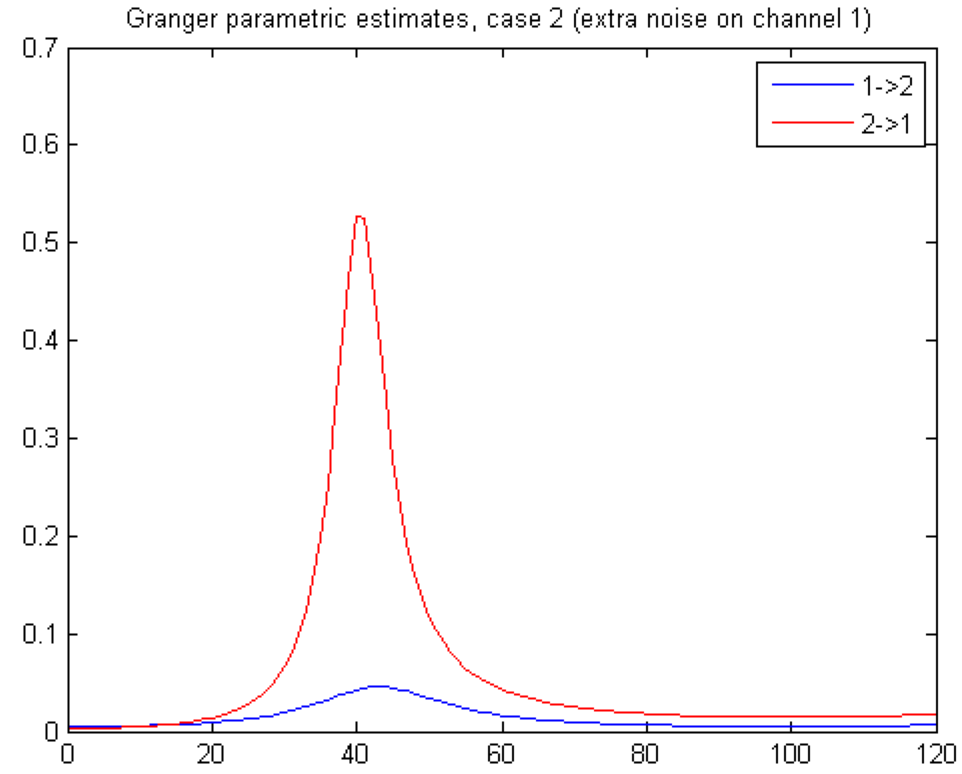

Granger parametric estimates, case 2 (extra noise on channel 1)
1->2
2->1
0.7
0.6
0.5
0.4
0.3
0.2
0.1
0
0
20
40
60
80
100
120

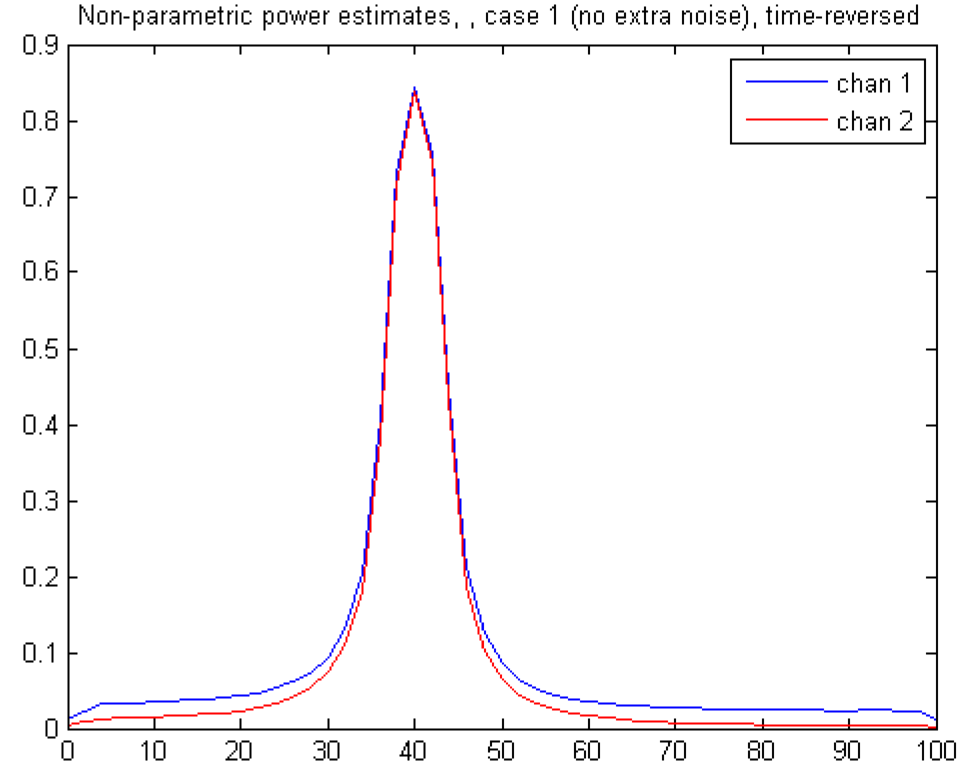
Non-parametric power estimates, , case 1 (no extra noise), time-reversed
chan 1
chan 2
0
0.1
0.2
0.3
0.4
0.5
0.6
0.7
0.8
0.9
0
10
20
30
40
50
60
70
80
90
100

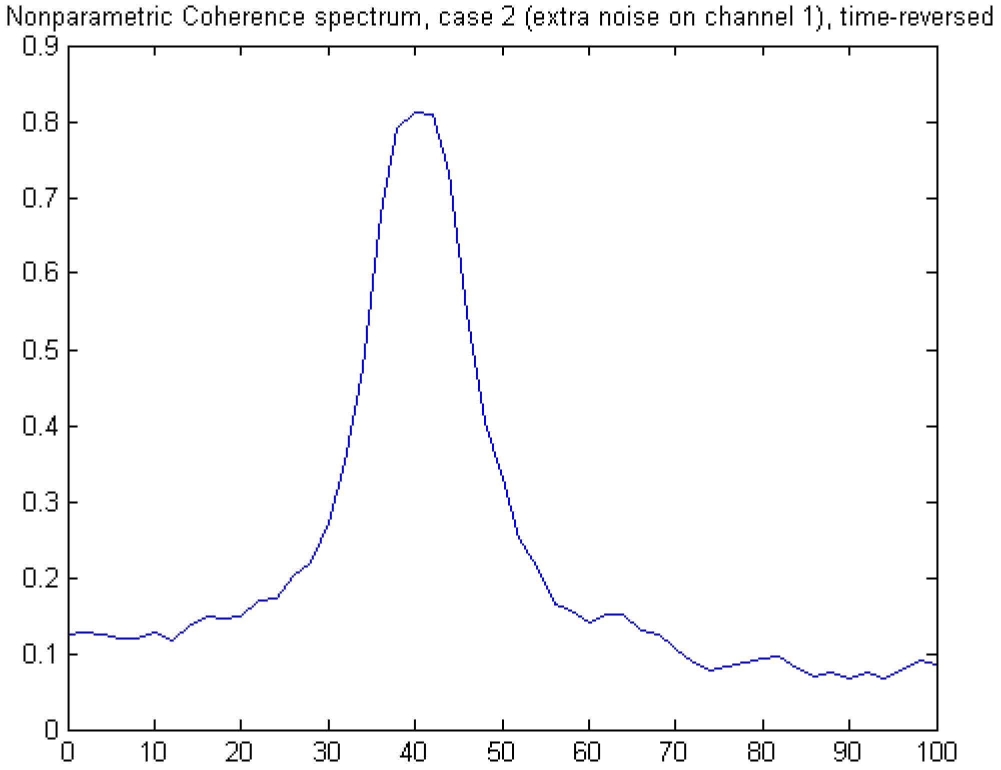
Nonparametric Coherence spectrum, case 2 (extra noise on channel 1), time-reversed
0
0.1
0.2
0.3
0.4
0.5
0.6
0.7
0.8
0.9
0
10
20
30
40
50
60
70
80
90
100

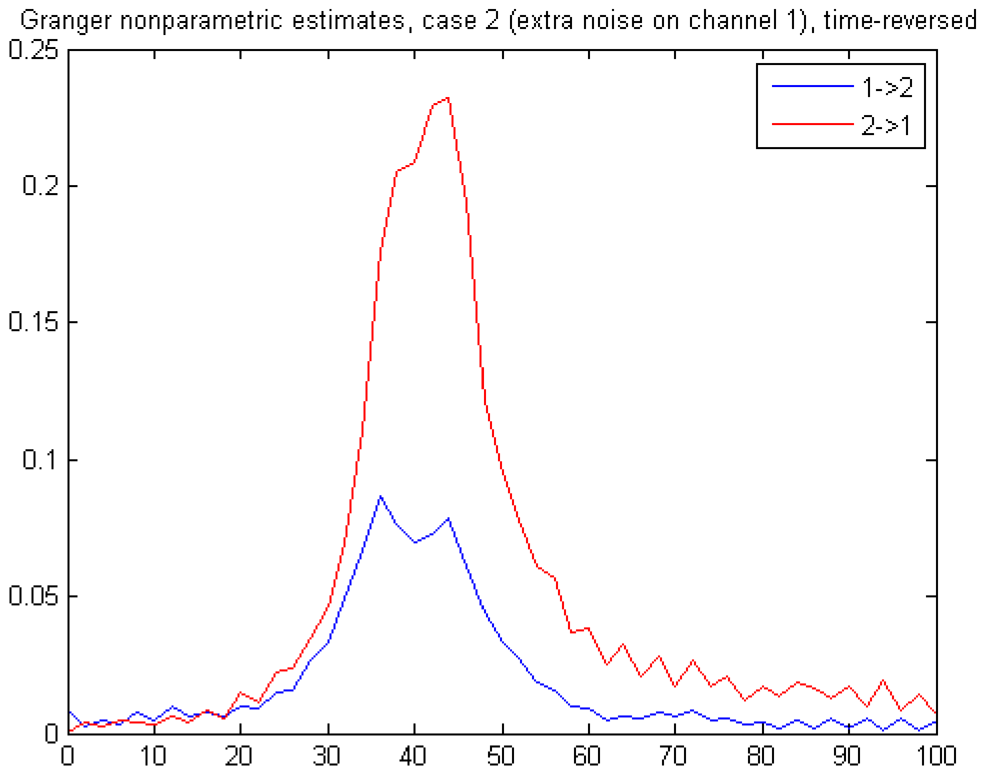
Granger nonparametric estimates, case 2 (extra noise on channel 1), time-reversed
1->2
2->1
0.25
0.2
0.15
0.1
0.05
0
0
10
20
30
40
50
60
70
80
90
100

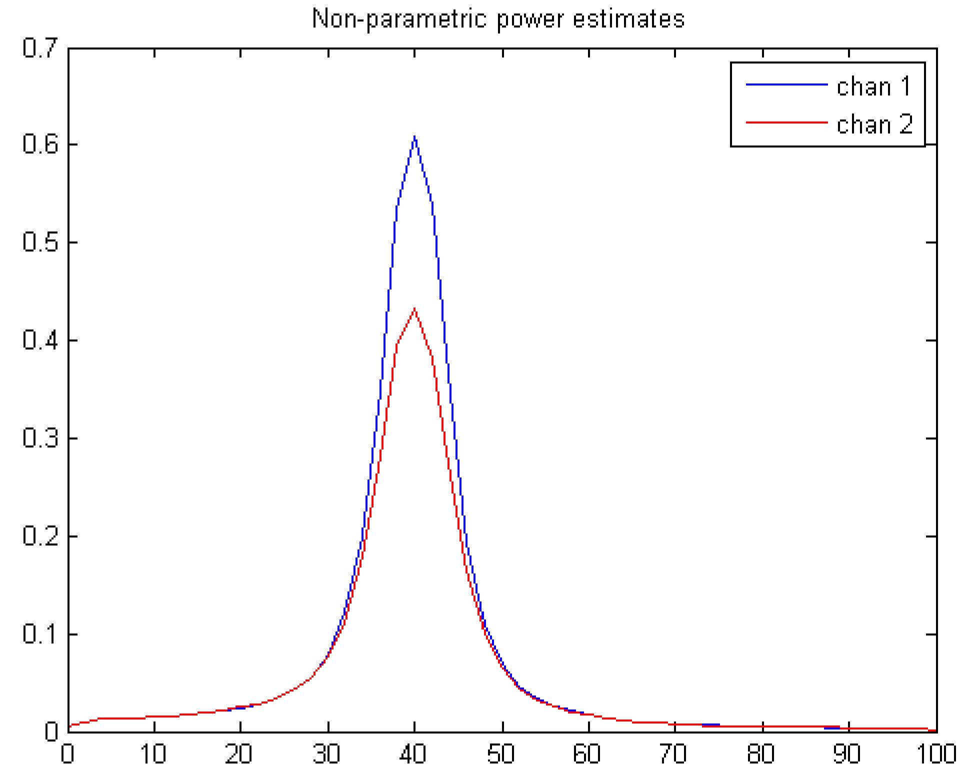
Non-parametric power estimates
chan 1
chan 2
0.7
0.6
0.5
0.4
0.3
0.2
0.1
0
0
10
20
30
40
50
60
70
80
90
100

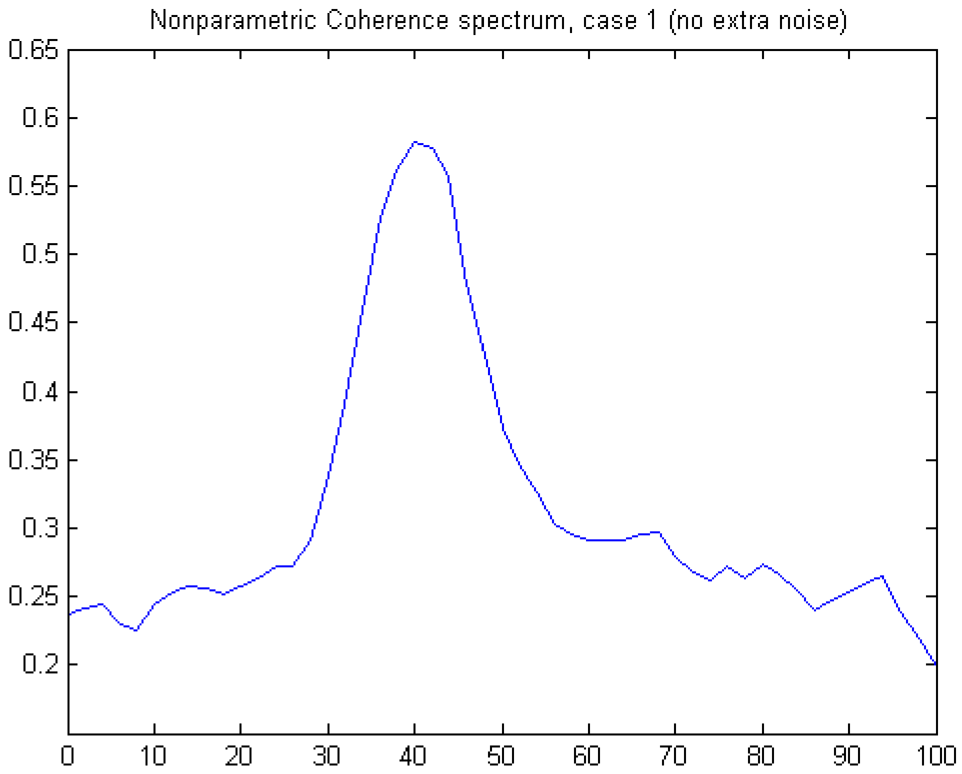
Nonparametric Coherence spectrum, case 1 (no extra noise)
0.65
0.6
0.55
0.5
0.45
0.4
0.35
0.3
0.25
0.2
0
10
20
30
40
50
60
70
80
90
100

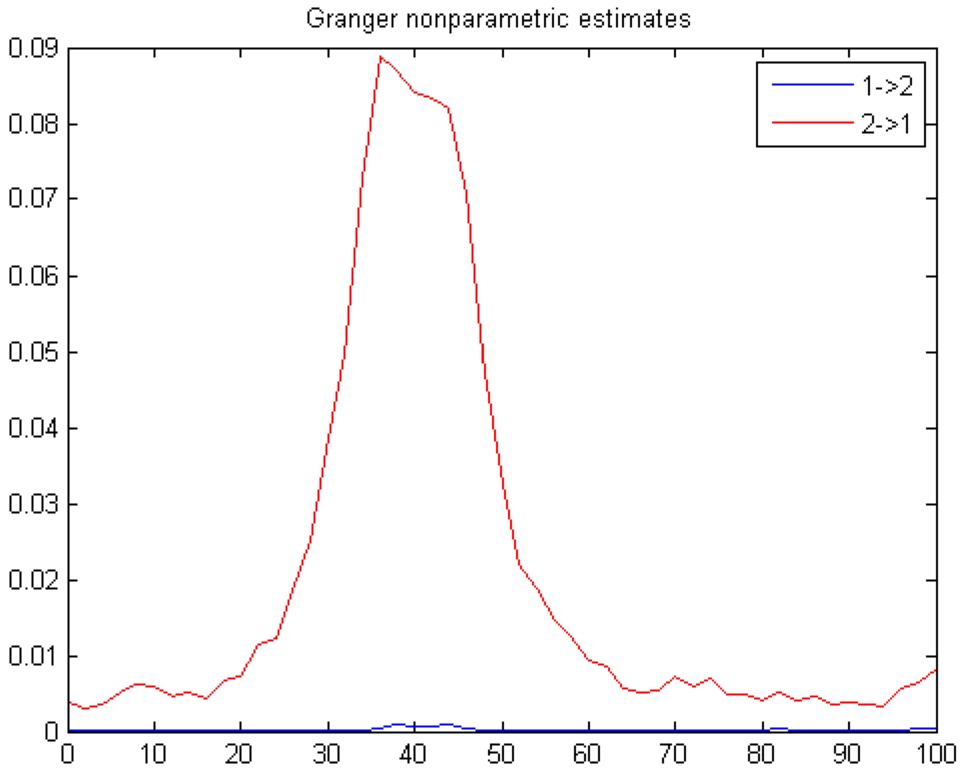
Granger nonparametric estimates
1->2
2->1
0.09
0.08
0.07
0.06
0.05
0.04
0.03
0.02
0.01
0
0
10
20
30
40
50
60
70
80
90
100

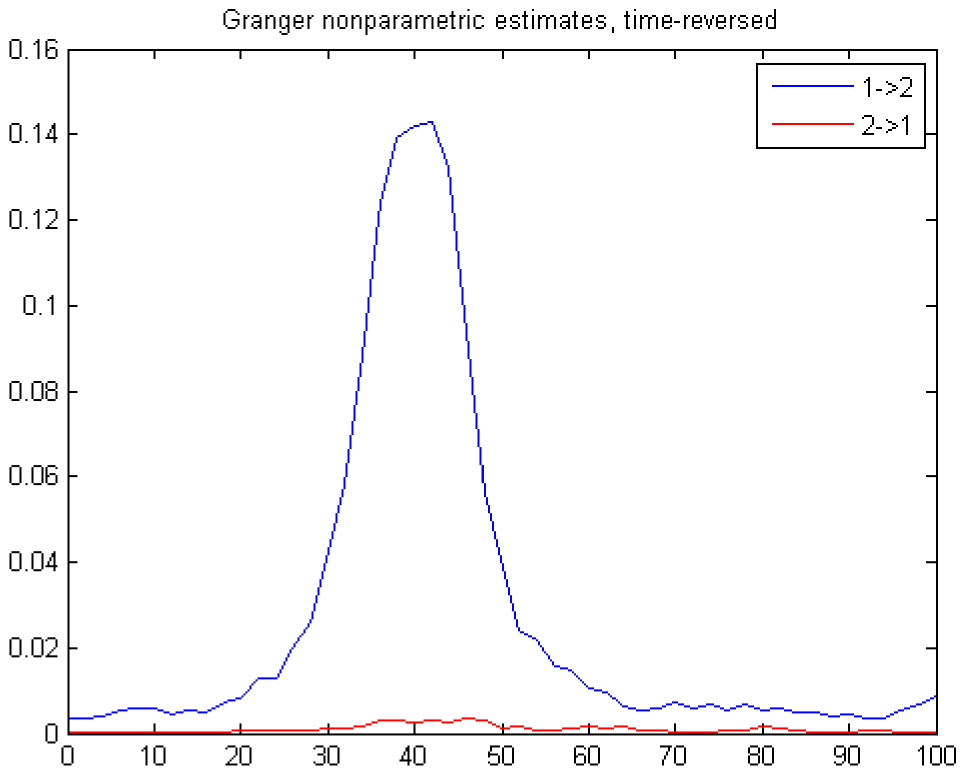

Published with MATLAB® R2013a
