# Supplementary material for: A Tutorial Review of Functional Connectivity Analysis Methods and Their Interpretational Pitfalls
Source: Front Syst Neurosci. 2016 Jan 8;9:175. doi: 10.3389/fnsys.2015.00175 (PMC4705224; doi:10.3389/fnsys.2015.00175)
Supplement: Supplementary file 6 [file sim_volumeconduction.pdf]

```
% Simulate some data, 50 channels, no connectivity, 2 channels with high power
cfg_sim              = [];
cfg_sim.method       = 'ar';
cfg_sim.ntrials      = 500;
cfg_sim.triallength = 1;
cfg_sim.fsample      = 200;
cfg_sim.nsignal      = 50;
cfg_sim.params(:,:,1) = diag(0.55*ones(cfg_sim.nsignal,1));
cfg_sim.params(:,:,2) = diag(-0.8*ones(cfg_sim.nsignal,1));
cfg_sim.noisecov       = diag(0.05*ones(cfg_sim.nsignal,1));
cfg_sim.noisecov(16,16) = 1;
cfg_sim.noisecov(35,35) = 1;

data = ft_connectivitysimulation(cfg_sim);

% create the 'mixing' montage
montage           = [];
montage.labelorg = data.label;
montage.labelnew = data.label;
montage.tra       = convn(eye(50),hanning(31),'same');
data              = ft_apply_montage(data, montage);

% calculate the fourier coefficients (non-parametric derivation of power)
cfg_freq         = [];
cfg_freq.method = 'mtmfft';
cfg_freq.taper  = 'dpss';
cfg_freq.output = 'fourier';
cfg_freq.tapsmofrq = 5;
cfg_freq.foilim = [0 100];
freq       = ft_freqanalysis(cfg_freq, data);

% shortcut to obtain the cross-spectral density between all channel pairs
freq       = ft_checkdata(freq, 'cmbrepresentation', 'fullfast');
```

PRO version Are you a developer? Try out the HTML to PDF API pdfcrowd.com

```
% coherence calculation
cfg_conn         = [];
cfg_conn.method  = 'coh';
cfg_conn.complex = 'abs';
coh1             = ft_connectivityanalysis(cfg_conn, freq);

% imaginary part of coherency calculation
cfg_conn.complex = 'absimag';
icoh             = ft_connectivityanalysis(cfg_conn, freq);

% these images now show the connectivity between all channel pairs, at 40
% Hz, and demonstrate the leakage (for the coherence metric) due to volume
% conduction.
figure; hold on;
imagesc(coh1.cohspctrm(:,:,41));
plot([0.5 50.5],[0.5 50.5],'w','linewidth',2);
plot([16 35],[16 35],'wo','markerfacecolor','w');
caxis([0 1]);
axis square; axis tight
set(gca,'fontsize', 25, 'Xtick', 10:10:40, 'Ytick', 10:10:40, 'tickdir', 'out');
title('coherence','fontsize',25);
colorbar('fontsize',25);

figure; hold on;
imagesc(icoh.cohspctrm(:,:,41));
plot([0.5 50.5],[0.5 50.5],'w','linewidth',2);
plot([16 35],[16 35],'wo','markerfacecolor','w');
caxis([0 0.05]);
axis square; axis tight
set(gca,'fontsize', 25, 'Xtick', 10:10:40, 'Ytick', 10:10:40, 'tickdir', 'out');
title('imag(coh)','fontsize',25);
colorbar('fontsize',25);

%%%%%%%%%%%%%%%%%%%%%%%%%%%%%%%%%%%%%%%%%%%%%%%%%%%%%%%%%%%%%%%%%%%%%%%%%%%%%%%%%%%%
```

PRO version Are you a developer? Try out the HTML to PDF API pdfcrowd.com

```
% Now create a connected 'data' using a generative model with cross terms:
cfg_sim.noisecov = diag(0.05*ones(cfg_sim.nsignal,1));
cfg_sim.noisecov(16,16) = 1;
cfg_sim.noisecov(35,35) = 1;
cfg_sim.params(16,35,1) = 0.25;
cfg_sim.params(16,35,2) = -0.1; %connect these sources, with a time lag
data  = ft_apply_montage(ft_connectivitysimulation(cfg_sim), montage);

% Cross-spectral density computation
freq  = ft_checkdata(ft_freqanalysis(cfg_freq, data),  'cmbrepresentation', 'fullfast');

% Coherence calculation
cfg_conn.complex = 'abs';
coh              = ft_connectivityanalysis(cfg_conn, freq);
cfg_conn.complex = 'absimag';
icoh             = ft_connectivityanalysis(cfg_conn, freq);

figure;hold on;
imagesc(coh.cohspctrm(:,:,41));
plot([0.5 50.5],[0.5 50.5],'w','linewidth',2);
plot([16 35],[16 35],'wo','markerfacecolor','w');
plot([0.5 50.5],[16 16],'w--','linewidth',2);
plot([16 35],[16 16],'ws','markerfacecolor','w');
caxis([0 1]);
axis square; axis tight
set(gca,'fontsize', 25, 'Xtick', 10:10:40, 'Ytick', 10:10:40, 'tickdir', 'out');
title('coherence','fontsize',25);
colorbar('fontsize', 25);

figure;
plot(coh.cohspctrm(16,:,41),'k','linewidth',1); axis([1 50 0 1]);
ylabel('coherence','fontsize', 25);
xlabel('sensor','fontsize', 25);
set(gca,'Xtick',10:10:40,'Ytick',0.2:0.2:0.8,'tickdir','out','fontsize',25);
```

PRO version Are you a developer? Try out the HTML to PDF API pdfcrowd.com

```
figure;hold on;
imagesc(icoh.cohspctrm(:,:,41));
plot([0.5 50.5],[0.5 50.5],'w','linewidth',2);
plot([16 35],[16 35],'wo','markerfacecolor','w');
plot([0.5 50.5],[16 16],'w--','linewidth',2);
plot([16 35],[16 16],'ws','markerfacecolor','w');
caxis([0 0.5]);
axis square; axis tight
set(gca,'fontsize', 25, 'Xtick', 10:10:40, 'Ytick', 10:10:40, 'tickdir', 'out');
title('imag(coh)','fontsize',25);
colorbar('fontsize', 25);

figure;
plot(icoh.cohspctrm(16,:,41),'k','linewidth',1); axis([1 50 0 0.5]);
ylabel('imag(coh)','fontsize', 25);
xlabel('sensor','fontsize', 25);
set(gca,'fontsize', 25);
set(gca,'Xtick',10:10:40,'Ytick',0.1:0.1:0.4,'tickdir','out','fontsize',25);

figure;hold on;
imagesc(coh.cohspctrm(:,:,41)-coh1.cohspctrm(:,:,41));
plot([0.5 50.5],[0.5 50.5],'w','linewidth',2);
plot([16 35],[16 35],'wo','markerfacecolor','w');
plot([0.5 50.5],[16 16],'w--','linewidth',2);
plot([16 35],[16 16],'ws','markerfacecolor','w');
caxis([0 0.4]);
axis square; axis tight
set(gca,'fontsize', 25, 'Xtick', 10:10:40, 'Ytick', 10:10:40, 'tickdir', 'out');
title('coherence diff','fontsize',25);
colorbar('fontsize', 25);

figure; hold on;
plot(coh.cohspctrm(16,:,41),'k','linewidth',1); axis([1 50 0 1]);
plot(coh1.cohspctrm(16,:,41),'r','linewidth',1); axis([1 50 0 1]);
plot(coh.cohspctrm(16,:,41)-coh1.cohspctrm(16,:,41),'b','linewidth',1); axis([1 50 0 1]);
```

PRO version Are you a developer? Try out the HTML to PDF API pdfcrowd.com

```
ylabel('coherence','fontsize', 25);
xlabel('sensor','fontsize', 25);
set(gca,'fontsize', 25);
set(gca,'Xtick',10:10:40,'Ytick',0.2:0.2:0.8,'tickdir','out','fontsize',25);


%%%%%%%%%%%%%%%%%%%%%%%%%%%%%%%%%%%%%%%%%%%%%%%%%%%%%%%%%%%%%%%%%%%%%%%%%%%%%%
% Now create a connected 'data2' using a generative model with cross terms
% between sources 16 and 35, but with an additional source at location 27

cfg_sim.noisecov = diag(0.05*ones(cfg_sim.nsignal,1));
cfg_sim.noisecov(16,16) = 1;
cfg_sim.noisecov(35,35) = 1;
cfg_sim.noisecov(27,27) = 1;
cfg_sim.params(16,35,1) = 0.25;
cfg_sim.params(16,35,2) = -0.1; %connect these sources, with a time lag
data2  = ft_apply_montage(ft_connectivitysimulation(cfg_sim), montage);

% Cross-spectral density computation
freq2  = ft_checkdata(ft_freqanalysis(cfg_freq, data2),  'cmbrepresentation', 'fullfast');

% Coherence calculation
cfg_conn.complex = 'abs';
coh2             = ft_connectivityanalysis(cfg_conn, freq2);
cfg_conn.complex = 'absimag';
icoh2            = ft_connectivityanalysis(cfg_conn, freq2);

figure;hold on;
imagesc(coh.cohspctrm(:,:,41)-coh2.cohspctrm(:,:,41));
plot([0.5 50.5],[0.5 50.5],'w','linewidth',2);
plot([16 35],[16 35],'wo','markerfacecolor','w');
plot([0.5 50.5],[11 11],'w--','linewidth',2);
plot([11 25],[11 11],'ws','markerfacecolor','w');
caxis([-0.15 0.15]);
axis square; axis tight
```

PRO version Are you a developer? Try out the HTML to PDF API pdfcrowd.com

```
set(gca,'fontsize', 25, 'Xtick', 10:10:40, 'Ytick', 10:10:40, 'tickdir', 'out');
title('coherence diff','fontsize',25);
colorbar('fontsize', 25);

figure; hold on;
plot(coh.cohspctrm(11,:,41),'k','linewidth',1); axis([1 50 -0.1 1]);
plot(coh2.cohspctrm(11,:,41),'r','linewidth',1); axis([1 50 -0.1 1]);
plot(coh.cohspctrm(11,:,41)-coh2.cohspctrm(11,:,41),'b','linewidth',1); axis([1 50 -0.1 1]);
ylabel('coherence','fontsize', 25);
xlabel('sensor','fontsize', 25);
set(gca,'fontsize', 25);
set(gca,'Xtick',10:10:40,'Ytick',0.2:0.2:0.8,'tickdir','out','fontsize',25);

figure;hold on;
imagesc(icoh.cohspctrm(:,:,41)-icoh2.cohspctrm(:,:,41));
plot([0.5 50.5],[0.5 50.5],'w','linewidth',2);
plot([16 35],[16 35],'wo','markerfacecolor','w');
plot([0.5 50.5],[22 22],'w--','linewidth',2);
plot([22 30],[22 22],'ws','markerfacecolor','w');
caxis([-0.1 0.1]);
axis square; axis tight
set(gca,'fontsize', 25, 'Xtick', 10:10:40, 'Ytick', 10:10:40, 'tickdir', 'out');
title('imag(coh) diff','fontsize',25);
colorbar('fontsize', 25);

figure; hold on;
plot(icoh.cohspctrm(22,:,41),'k','linewidth',1); axis([1 50 -0.05 0.5]);
plot(icoh2.cohspctrm(22,:,41),'r','linewidth',1); axis([1 50 -0.05 0.5]);
plot(icoh.cohspctrm(22,:,41)-icoh2.cohspctrm(22,:,41),'b','linewidth',1); axis([1 50 -0.05 0.5]);
ylabel('imag(coh)','fontsize', 25);
xlabel('sensor','fontsize', 25);
set(gca,'fontsize', 25);
set(gca,'Xtick',10:10:40,'Ytick',0.1:0.1:0.4,'tickdir','out','fontsize',25);

%%%%%%%%%%%%%%%%%%%%%%%%%%%%%%%%%%%%%%%%%%%%%%%%%%%%%%%%%%%%%%%%%%%%%%%%%%%%%%%
```

PRO version Are you a developer? Try out the HTML to PDF API pdfcrowd.com

```
% Now create a 'data' and 'data2' using a similar generative model, but
% with one of the 'active sources' having a larger power in one of the
% 'conditions'
cfg_sim.noisecov(16,16) = 1;
cfg_sim.noisecov(35,35) = 1;
cfg_sim.noisecov(27,27) = 0.05;
data  = ft_apply_montage(ft_connectivitysimulation(cfg_sim), montage);
cfg_sim.noisecov(16,16) = 1;
cfg_sim.noisecov(35,35) = 2;
data2 = ft_apply_montage(ft_connectivitysimulation(cfg_sim), montage);

% Cross-spectral density computation
freq  = ft_checkdata(ft_freqanalysis(cfg_freq, data),  'cmbrepresentation', 'fullfast');
freq2 = ft_checkdata(ft_freqanalysis(cfg_freq, data2), 'cmbrepresentation', 'fullfast');

% Coherence calculation
cfg_conn.complex = 'abs';
coh              = ft_connectivityanalysis(cfg_conn, freq);
coh2             = ft_connectivityanalysis(cfg_conn, freq2);
cfg_conn.complex = 'absimag';
icoh             = ft_connectivityanalysis(cfg_conn, freq);
icoh2            = ft_connectivityanalysis(cfg_conn, freq2);

figure;hold on;
imagesc(coh2.cohspctrm(:,:,41)-coh.cohspctrm(:,:,41));
plot([0.5 50.5],[0.5 50.5],'w','linewidth',2);
plot([16 35],[16 35],'wo','markerfacecolor','w');
plot([0.5 50.5],[16 16],'w--','linewidth',2);
plot([16 35],[16 16],'ws','markerfacecolor','w');
caxis([-0.2 0.2]);
axis square; axis tight
set(gca,'fontsize', 25, 'Xtick', 10:10:40, 'Ytick', 10:10:40, 'tickdir', 'out');
title('coherence diff','fontsize',25);
colorbar('fontsize', 25);
```

PRO version Are you a developer? Try out the HTML to PDF API pdfcrowd.com

```
figure; hold on;
plot(coh2.cohspctrm(16,:,41),'k','linewidth',1); axis([1 50 -0.1 1]);
plot(coh.cohspctrm(16,:,41),'r','linewidth',1); axis([1 50 -0.1 1]);
plot(coh2.cohspctrm(16,:,41)-coh.cohspctrm(16,:,41),'b','linewidth',1); axis([1 50 -0.1 1]);
plot([1 50],[0 0],'k--');
ylabel('coherence','fontsize', 25);
xlabel('sensor','fontsize', 25);
set(gca,'fontsize', 25);
set(gca,'Xtick',10:10:40,'Ytick',0.2:0.2:0.8,'tickdir','out','fontsize',25);

figure;hold on;
imagesc(icoh2.cohspctrm(:,:,41)-icoh.cohspctrm(:,:,41));
plot([0.5 50.5],[0.5 50.5],'w','linewidth',2);
plot([16 35],[16 35],'wo','markerfacecolor','w');
plot([0.5 50.5],[16 16],'w--','linewidth',2);
plot([16 35],[16 16],'ws','markerfacecolor','w');
caxis([-0.2 0.2]);
axis square; axis tight
set(gca,'fontsize', 25, 'Xtick', 10:10:40, 'Ytick', 10:10:40, 'tickdir', 'out');
title('imag(coh) diff','fontsize',25);
colorbar('fontsize', 25);

figure; hold on;
plot(icoh.cohspctrm(16,:,41),'r','linewidth',1); axis([1 50 -0.05 0.6]);
plot(icoh2.cohspctrm(16,:,41),'k','linewidth',1); axis([1 50 -0.05 0.6]);
plot(icoh2.cohspctrm(16,:,41)-icoh.cohspctrm(16,:,41),'b','linewidth',1); axis([1 50 -0.05 0.6]);
ylabel('imag(coh)','fontsize', 25);
xlabel('sensor','fontsize', 25);
set(gca,'fontsize', 25);
set(gca,'Xtick',10:10:40,'Ytick',0.1:0.1:0.4,'tickdir','out','fontsize',25);
```

```
the call to "ft_connectivitysimulation" took 1 seconds
processing trials
processing trial 500 from 500
```

PRO version Are you a developer? Try out the HTML to PDF API pdfcrowd.com

```
the input is raw data with 50 channels and 500 trials
Warning: the data does not contain a trial definition
Warning: reconstructing sampleinfo by assuming that the trials are consecutive
segments of a continuous recording
the call to "ft_selectdata" took 0 seconds
processing trials
processing trial 500/500 nfft: 200 samples, datalength: 200 samples, 9 tapers

the call to "ft_freqanalysis" took 3 seconds
selection crsspctrm along dimensions 1 and 2
the call to "ft_connectivityanalysis" took 0 seconds
selection crsspctrm along dimensions 1 and 2
the call to "ft_connectivityanalysis" took 0 seconds
the call to "ft_connectivitysimulation" took 1 seconds
processing trials
processing trial 500 from 500

the input is raw data with 50 channels and 500 trials
Warning: the data does not contain a trial definition
Warning: reconstructing sampleinfo by assuming that the trials are consecutive
segments of a continuous recording
the call to "ft_selectdata" took 0 seconds
processing trials
processing trial 500/500 nfft: 200 samples, datalength: 200 samples, 9 tapers

the call to "ft_freqanalysis" took 3 seconds
selection crsspctrm along dimensions 1 and 2
the call to "ft_connectivityanalysis" took 0 seconds
selection crsspctrm along dimensions 1 and 2
the call to "ft_connectivityanalysis" took 0 seconds
the call to "ft_connectivitysimulation" took 1 seconds
processing trials
processing trial 500 from 500
```

PRO version Are you a developer? Try out the HTML to PDF API pdfcrowd.com

```
the input is raw data with 50 channels and 500 trials
Warning: the data does not contain a trial definition
Warning: reconstructing sampleinfo by assuming that the trials are consecutive
segments of a continuous recording
the call to "ft_selectdata" took 0 seconds
processing trials
processing trial 500/500 nfft: 200 samples, datalength: 200 samples, 9 tapers

the call to "ft_freqanalysis" took 3 seconds
selection crsspctrm along dimensions 1 and 2
the call to "ft_connectivityanalysis" took 0 seconds
selection crsspctrm along dimensions 1 and 2
the call to "ft_connectivityanalysis" took 0 seconds
the call to "ft_connectivitysimulation" took 1 seconds
processing trials
processing trial 500 from 500

the call to "ft_connectivitysimulation" took 1 seconds
processing trials
processing trial 500 from 500

the input is raw data with 50 channels and 500 trials
Warning: the data does not contain a trial definition
Warning: reconstructing sampleinfo by assuming that the trials are consecutive
segments of a continuous recording
the call to "ft_selectdata" took 0 seconds
processing trials
processing trial 500/500 nfft: 200 samples, datalength: 200 samples, 9 tapers

the call to "ft_freqanalysis" took 3 seconds
the input is raw data with 50 channels and 500 trials
Warning: the data does not contain a trial definition
Warning: reconstructing sampleinfo by assuming that the trials are consecutive
segments of a continuous recording
the call to "ft_selectdata" took 0 seconds
```

PRO version Are you a developer? Try out the HTML to PDF API pdfcrowd.com

```
processing trials
processing trial 500/500 nfft: 200 samples, datalength: 200 samples, 9 tapers

the call to "ft_freqanalysis" took 3 seconds
selection crsspctrm along dimensions 1 and 2
the call to "ft_connectivityanalysis" took 0 seconds
selection crsspctrm along dimensions 1 and 2
the call to "ft_connectivityanalysis" took 0 seconds
selection crsspctrm along dimensions 1 and 2
the call to "ft_connectivityanalysis" took 0 seconds
selection crsspctrm along dimensions 1 and 2
the call to "ft_connectivityanalysis" took 0 seconds
```

PRO version Are you a developer? Try out the HTML to PDF API pdfcrowd.com

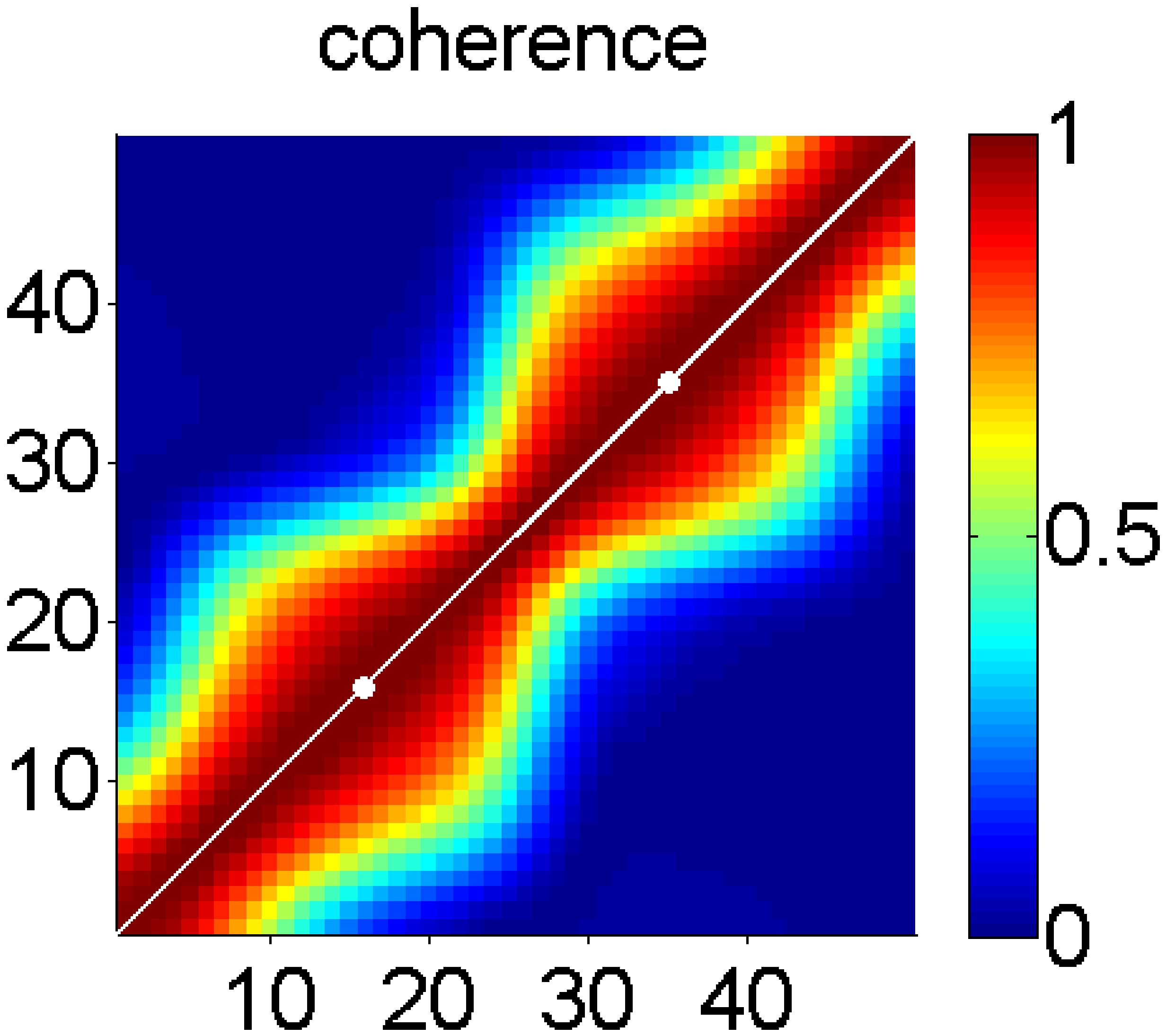

PRO version Are you a developer? Try out the HTML to PDF API pdfcrowd.com

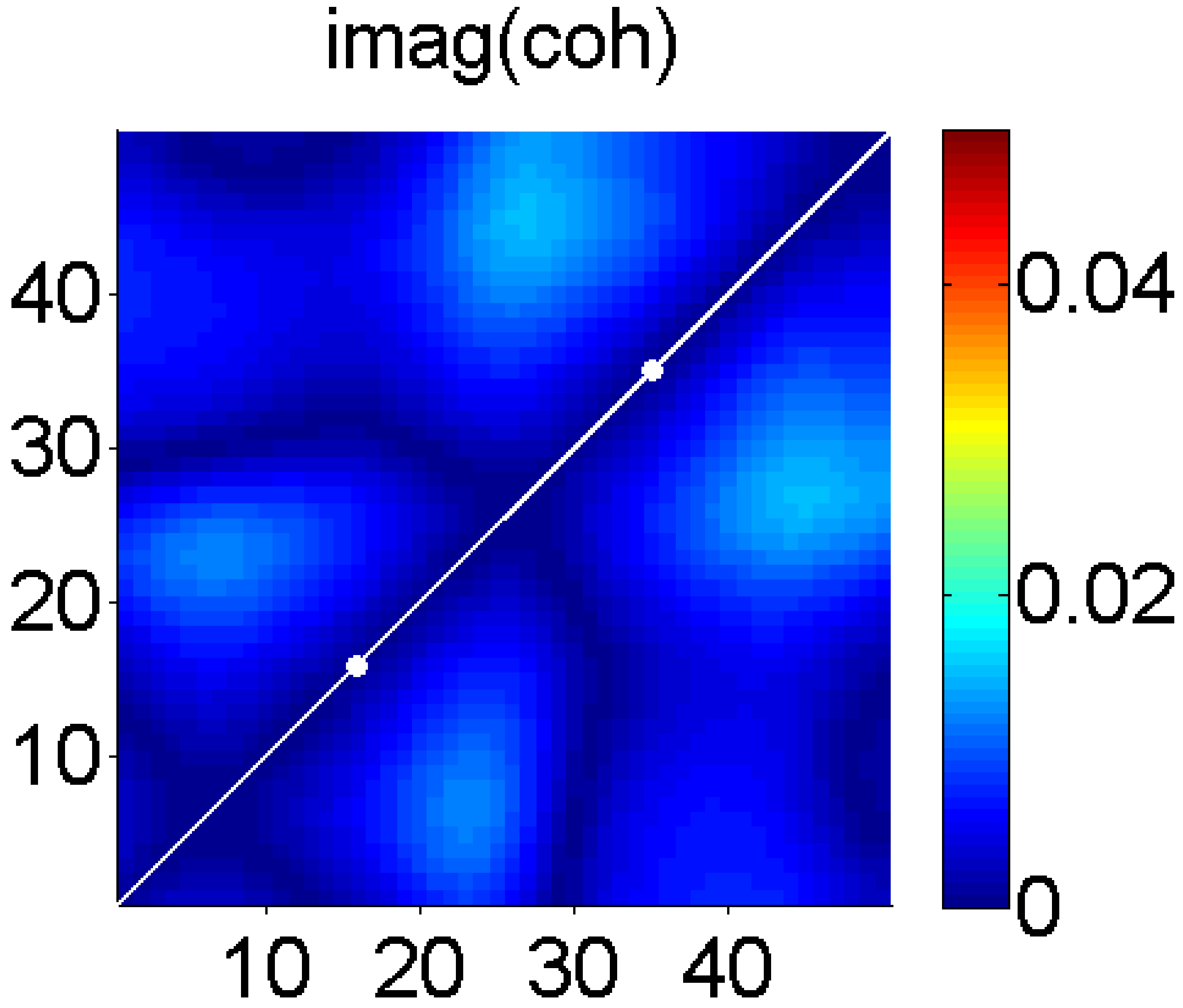

PRO version Are you a developer? Try out the HTML to PDF API pdfcrowd.com

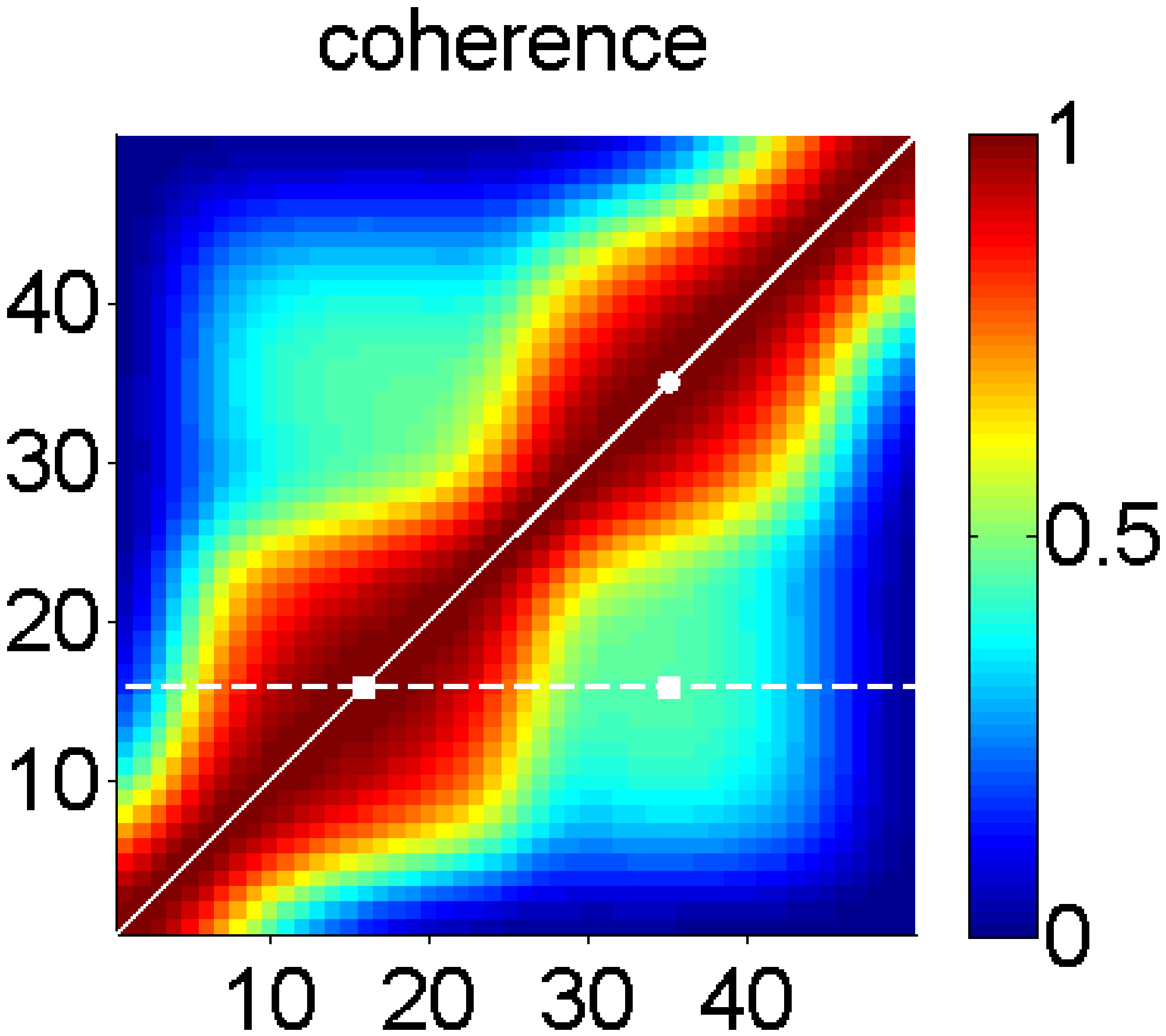

PRO version Are you a developer? Try out the HTML to PDF API pdfcrowd.com

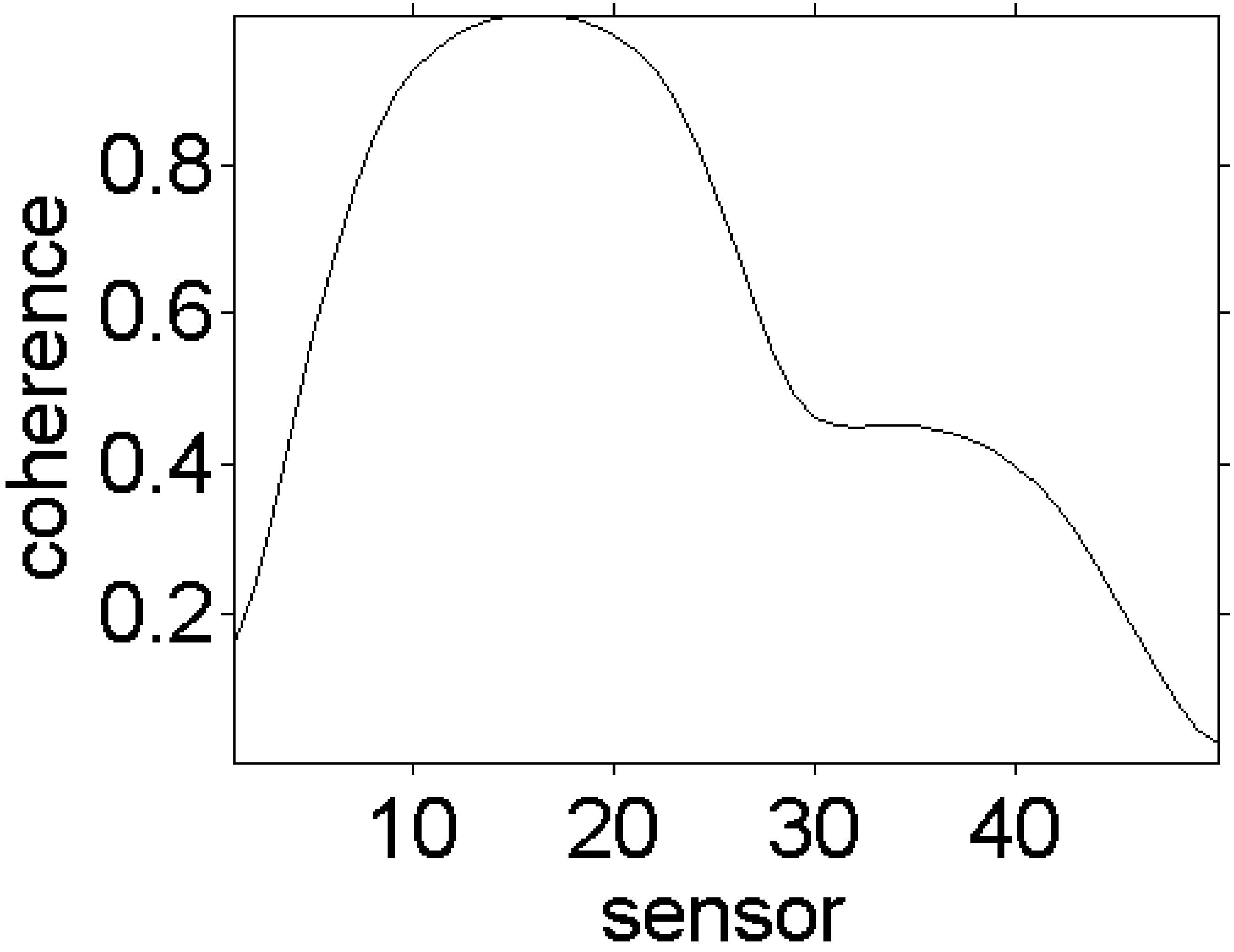

PRO version Are you a developer? Try out the HTML to PDF API pdfcrowd.com

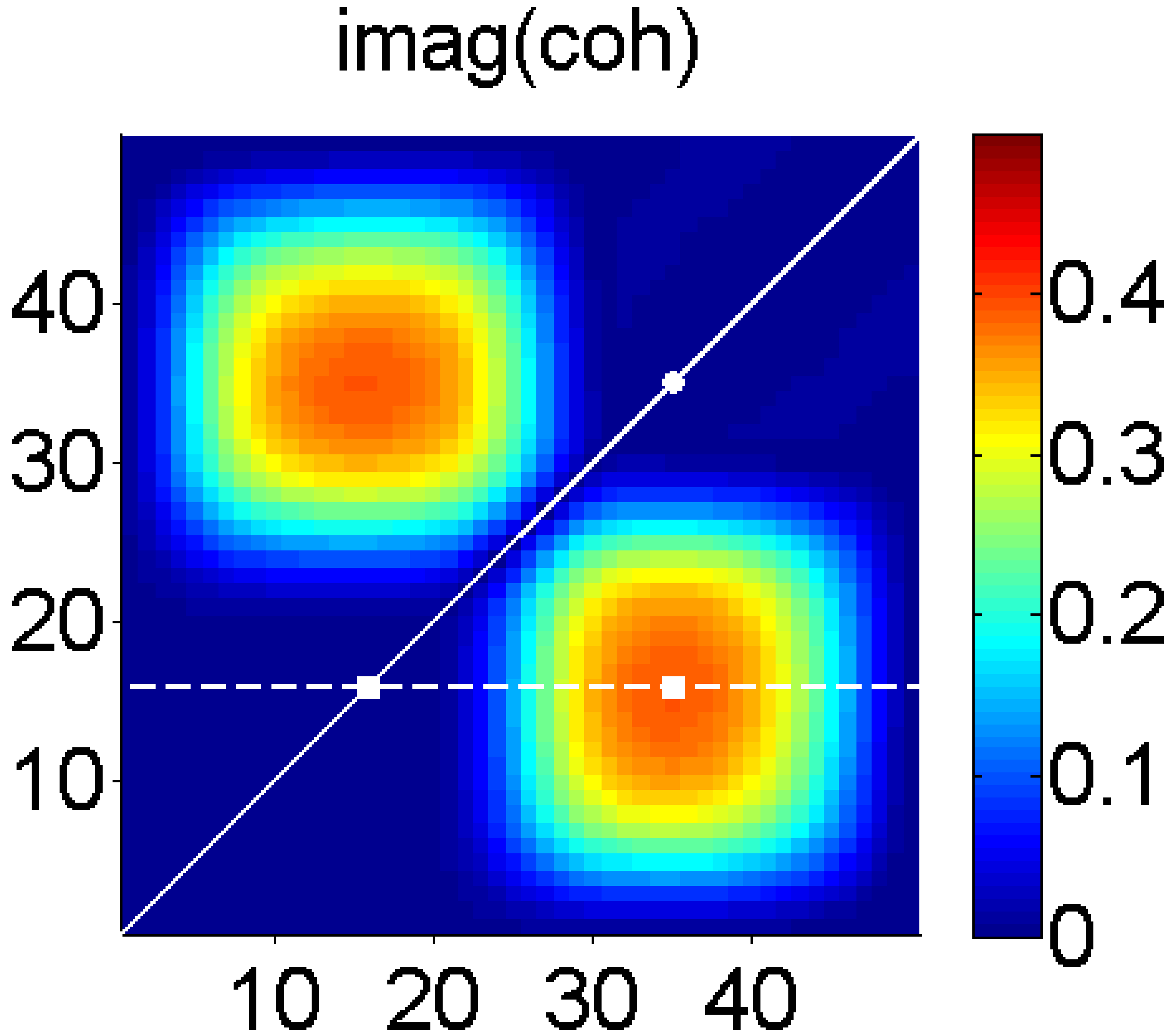

PRO version Are you a developer? Try out the HTML to PDF API pdfcrowd.com

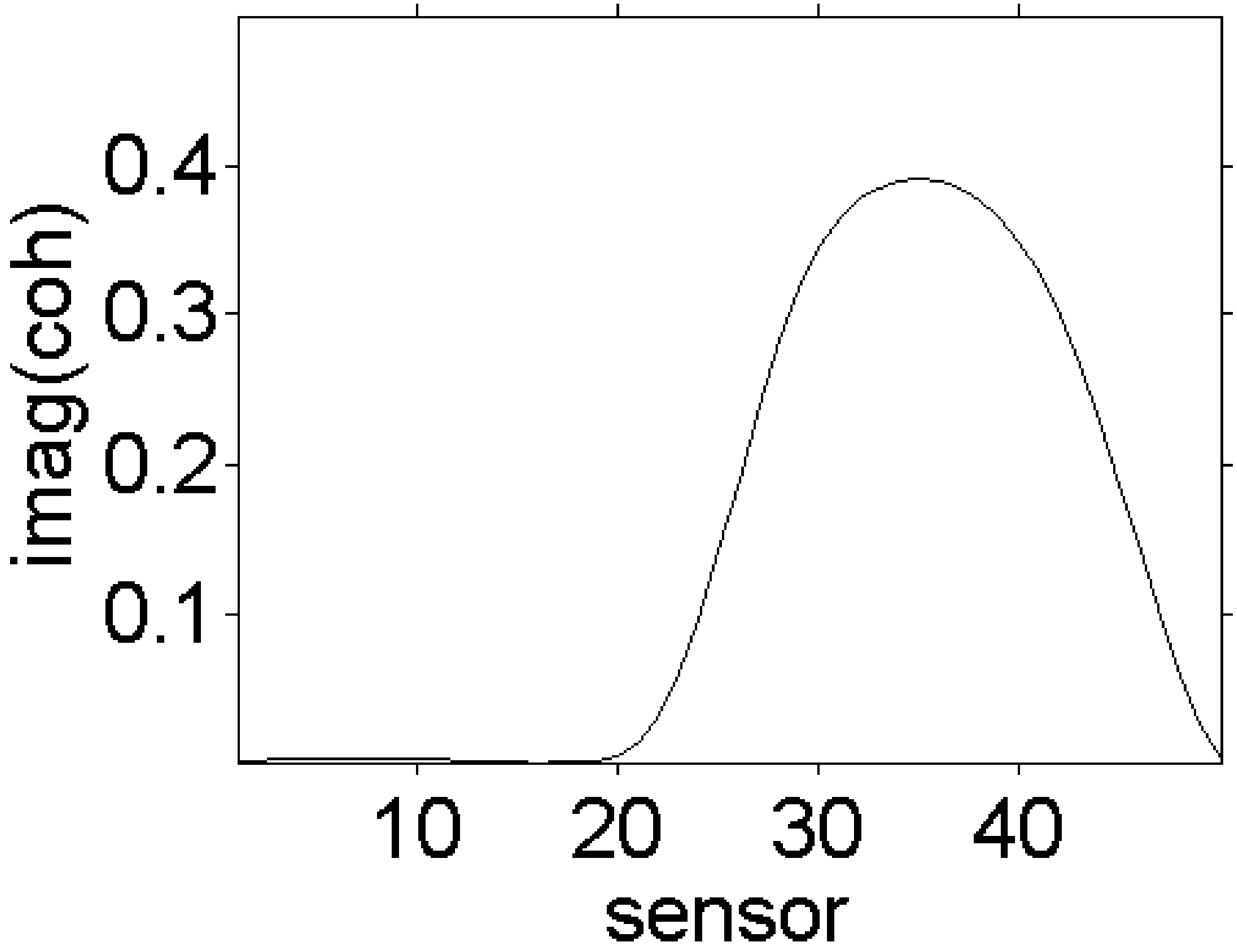

PRO version Are you a developer? Try out the HTML to PDF API pdfcrowd.com

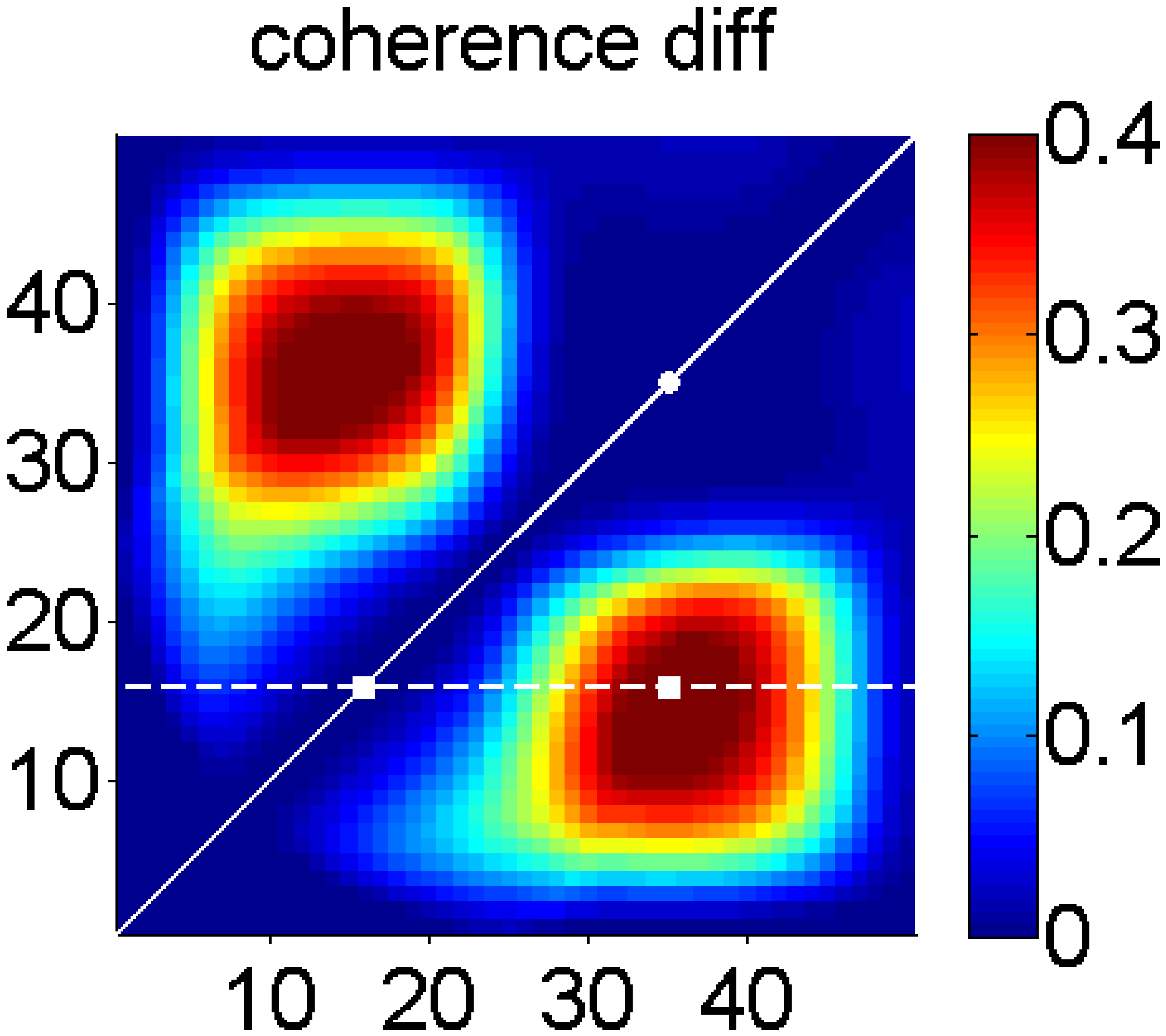

PRO version Are you a developer? Try out the HTML to PDF API pdfcrowd.com

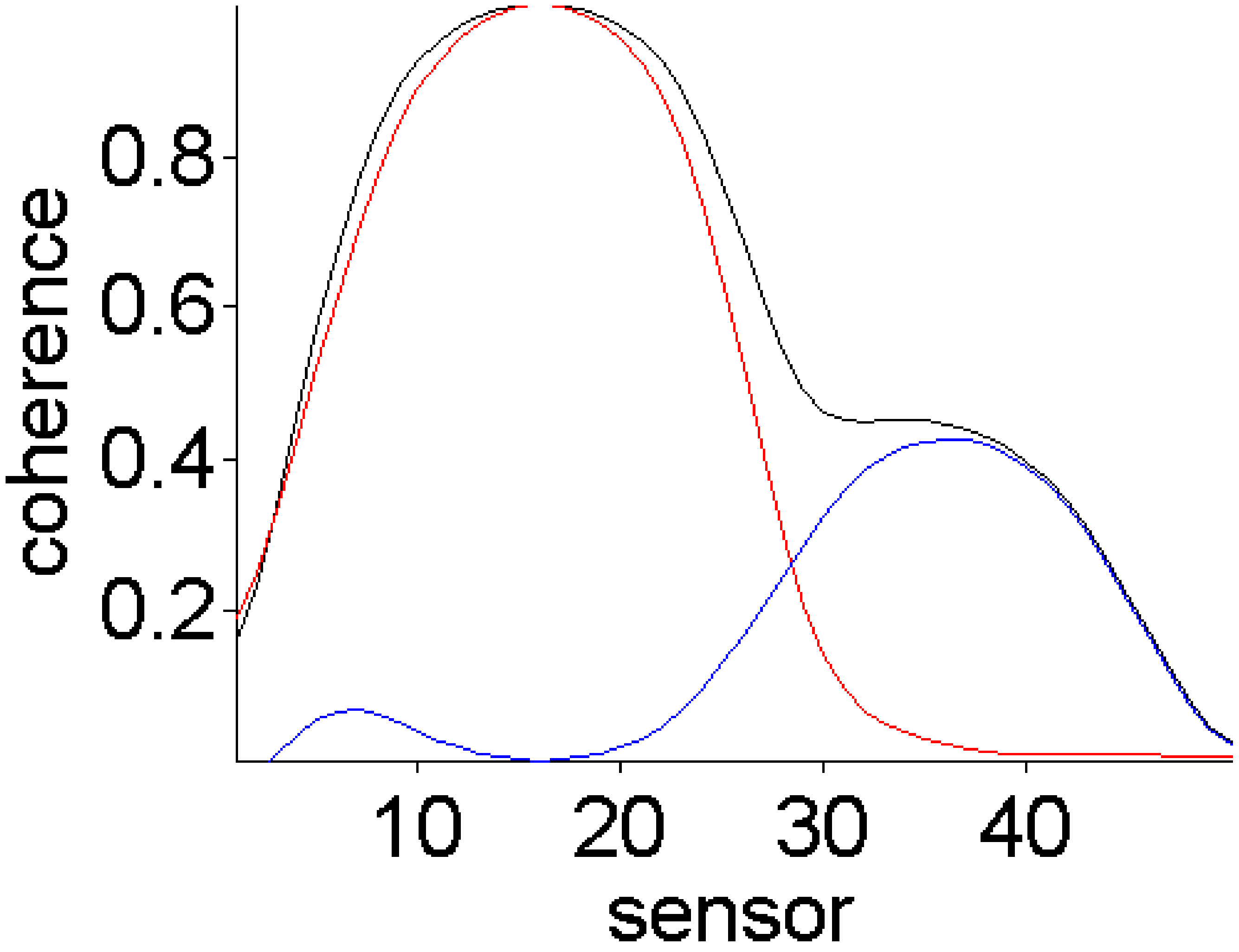

PRO version Are you a developer? Try out the HTML to PDF API pdfcrowd.com

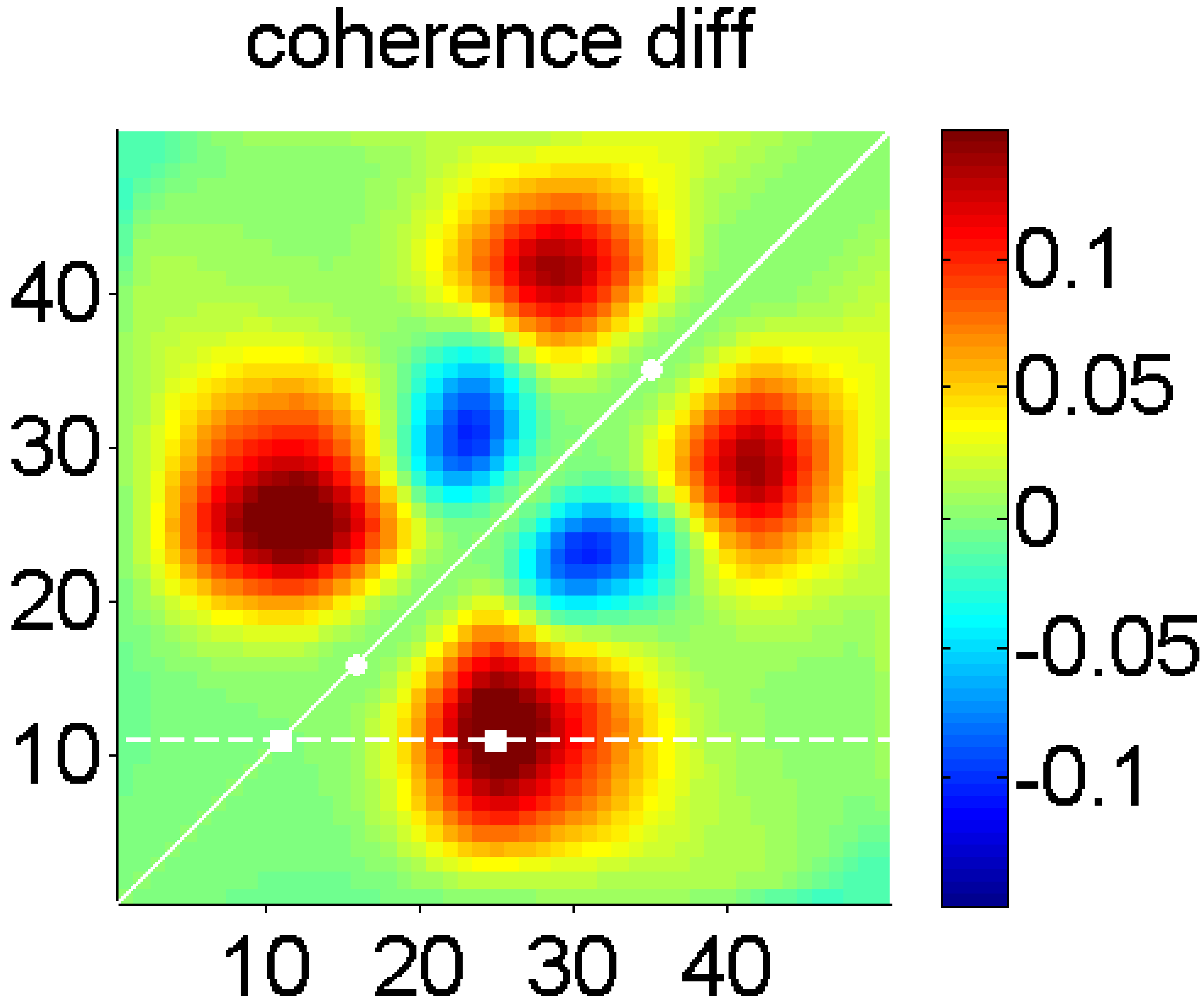

PRO version Are you a developer? Try out the HTML to PDF API pdfcrowd.com

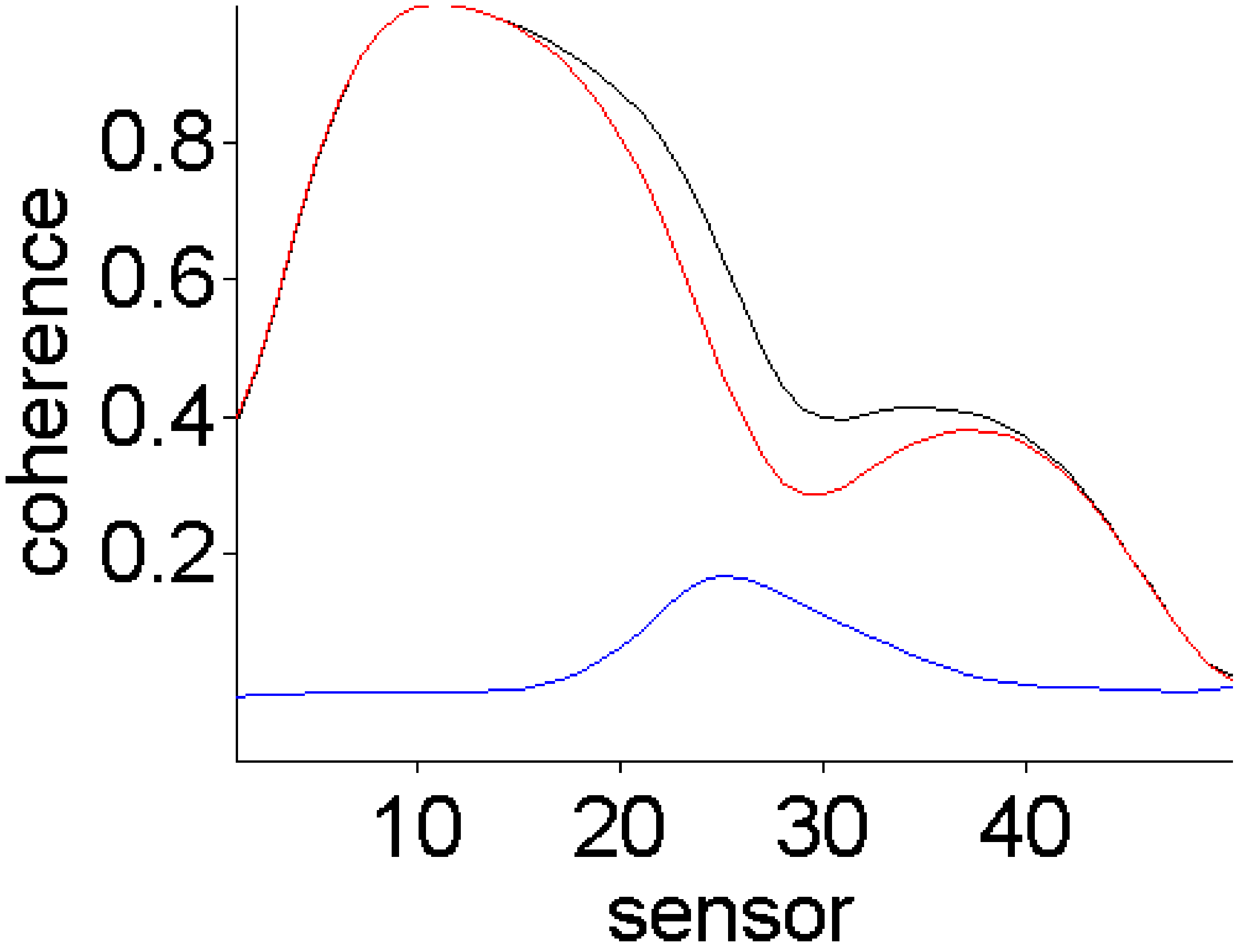

PRO version Are you a developer? Try out the HTML to PDF API pdfcrowd.com

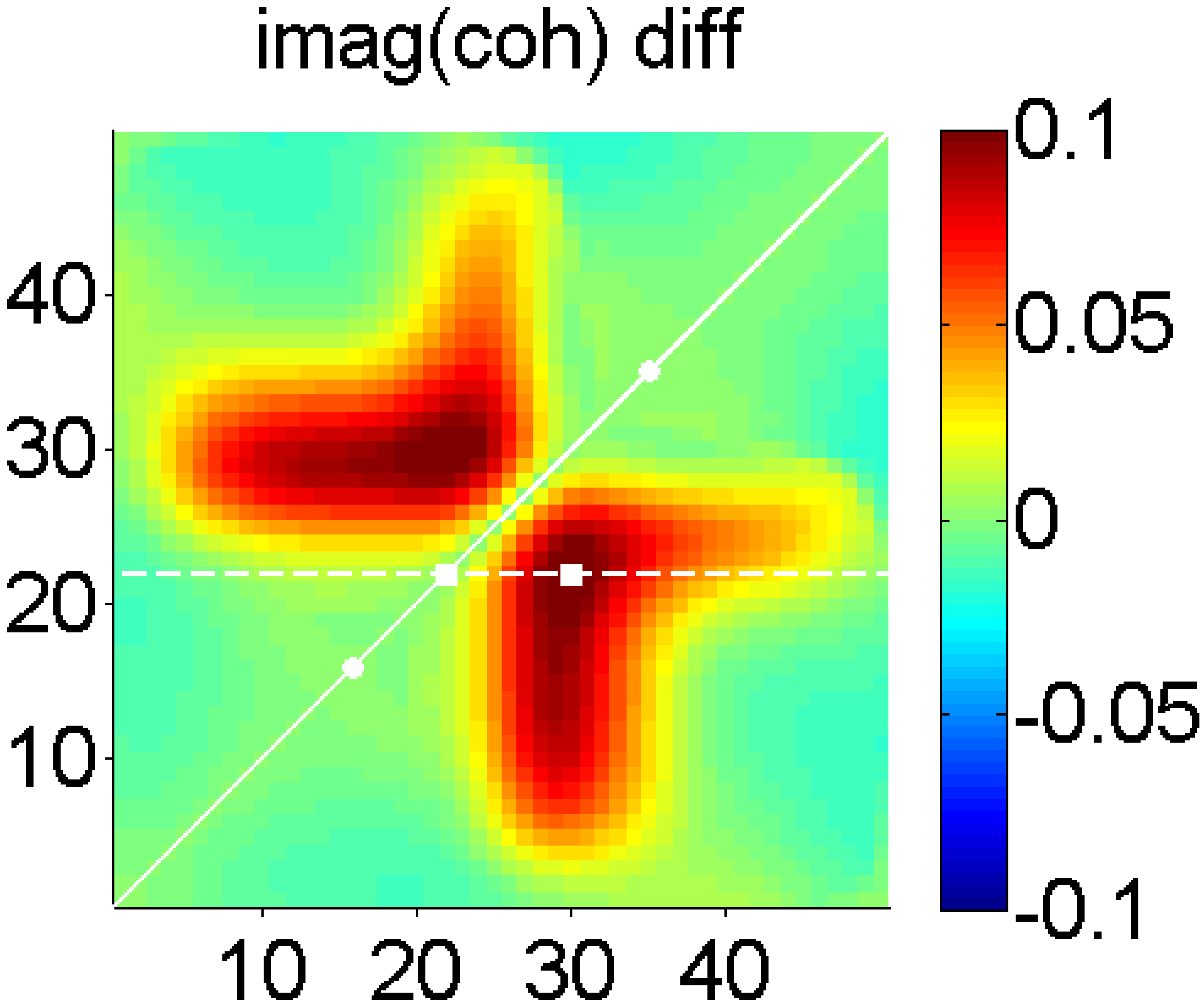

PRO version Are you a developer? Try out the HTML to PDF API pdfcrowd.com

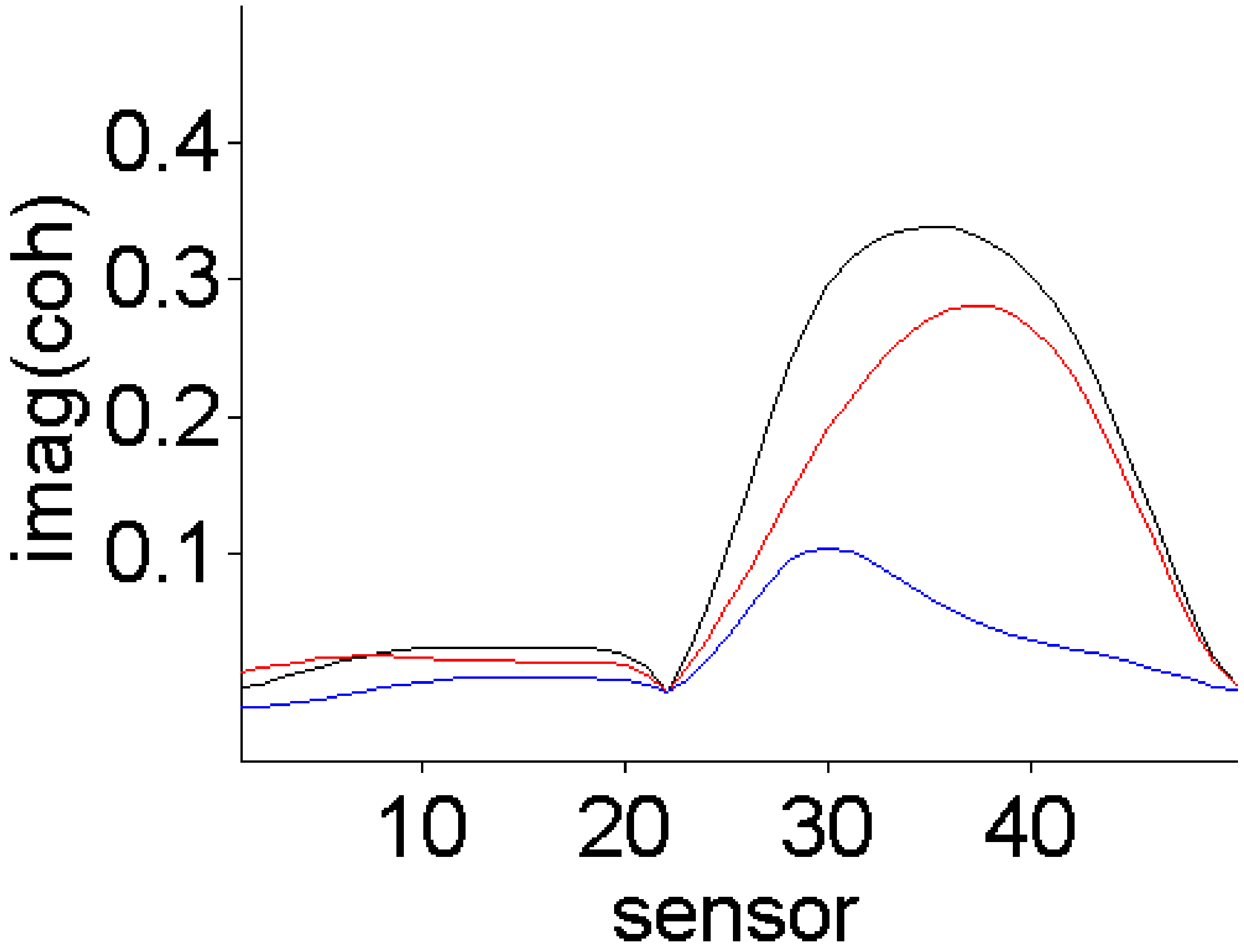

PRO version Are you a developer? Try out the HTML to PDF API pdfcrowd.com

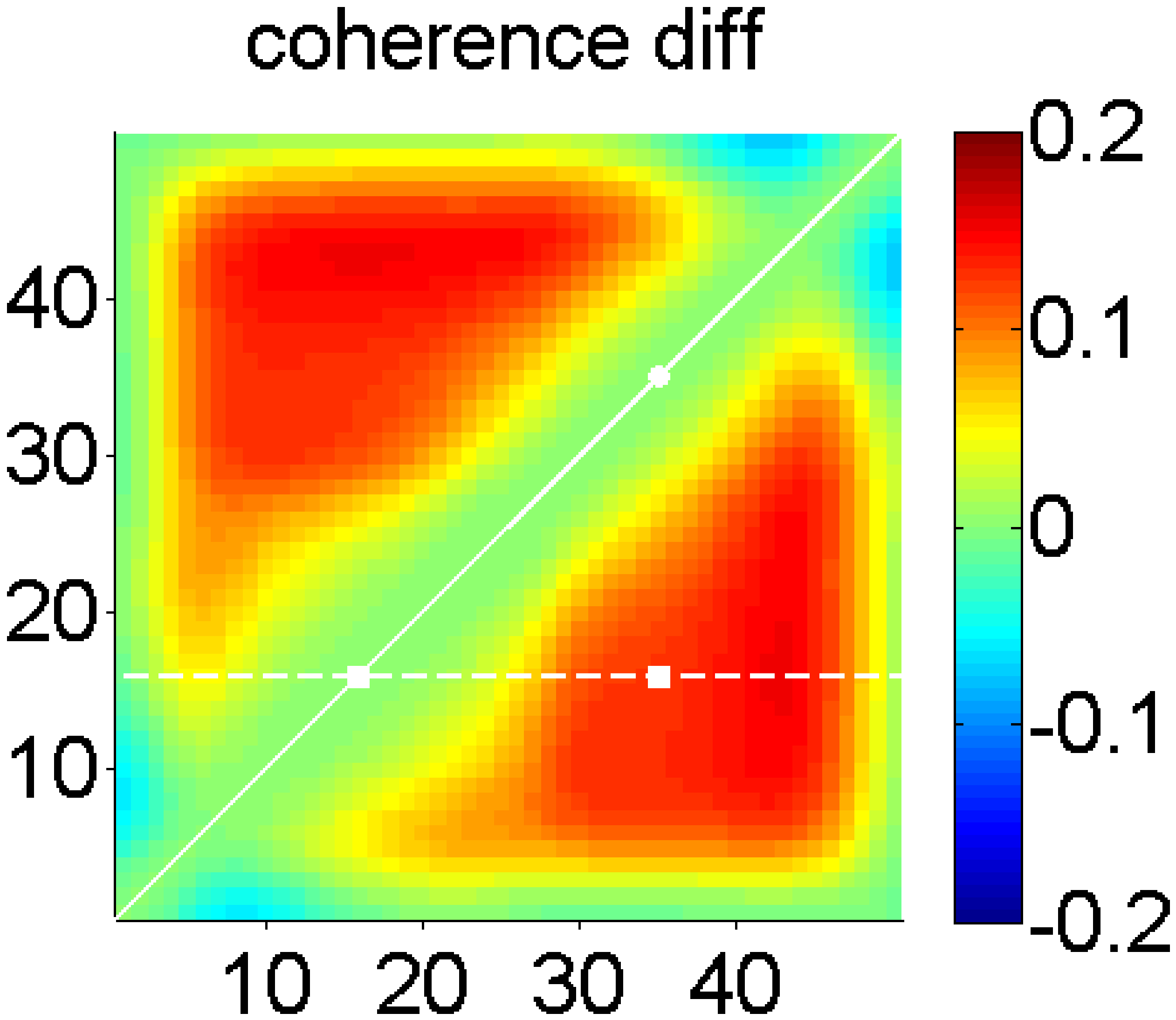

PRO version Are you a developer? Try out the HTML to PDF API pdfcrowd.com

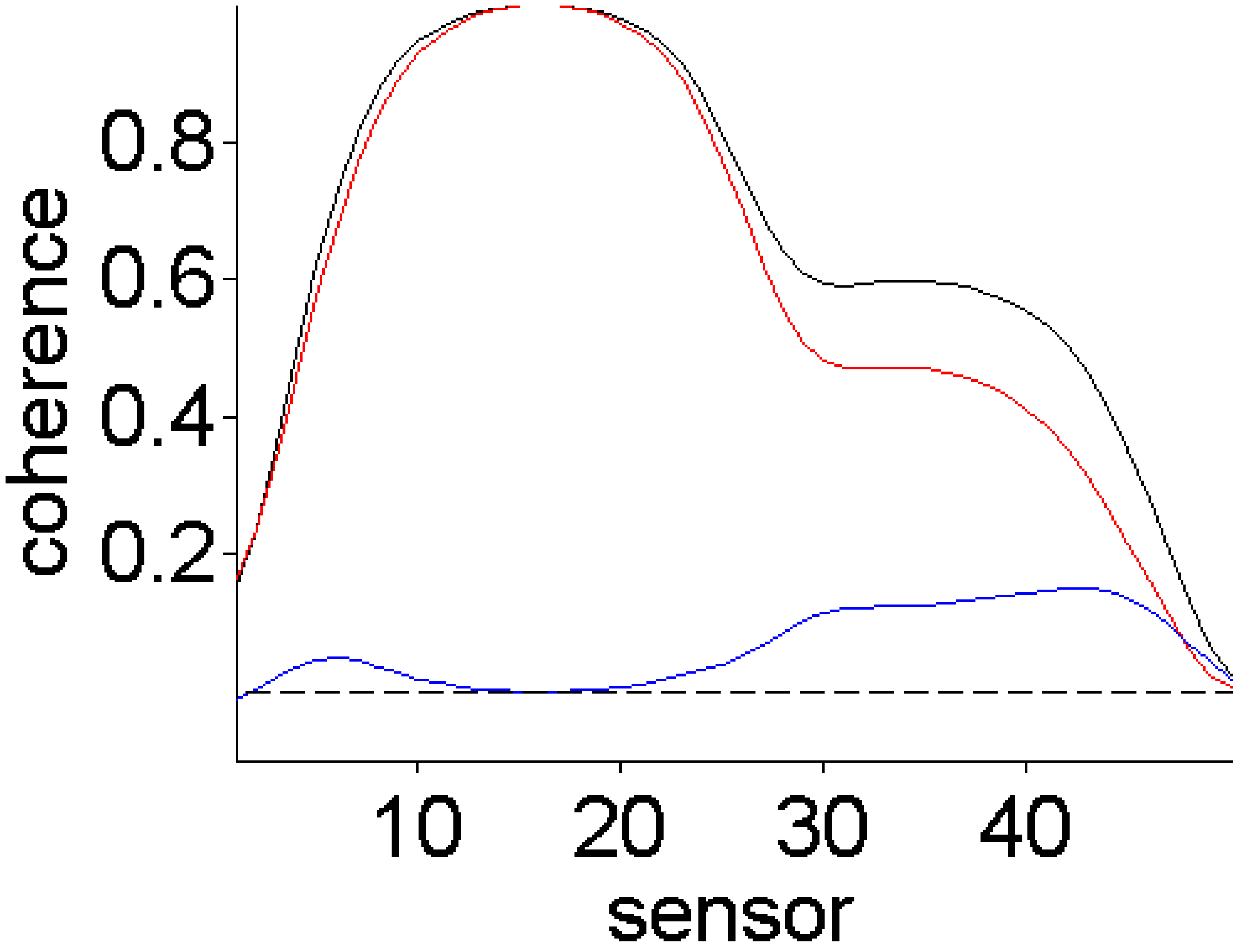

PRO version Are you a developer? Try out the HTML to PDF API pdfcrowd.com

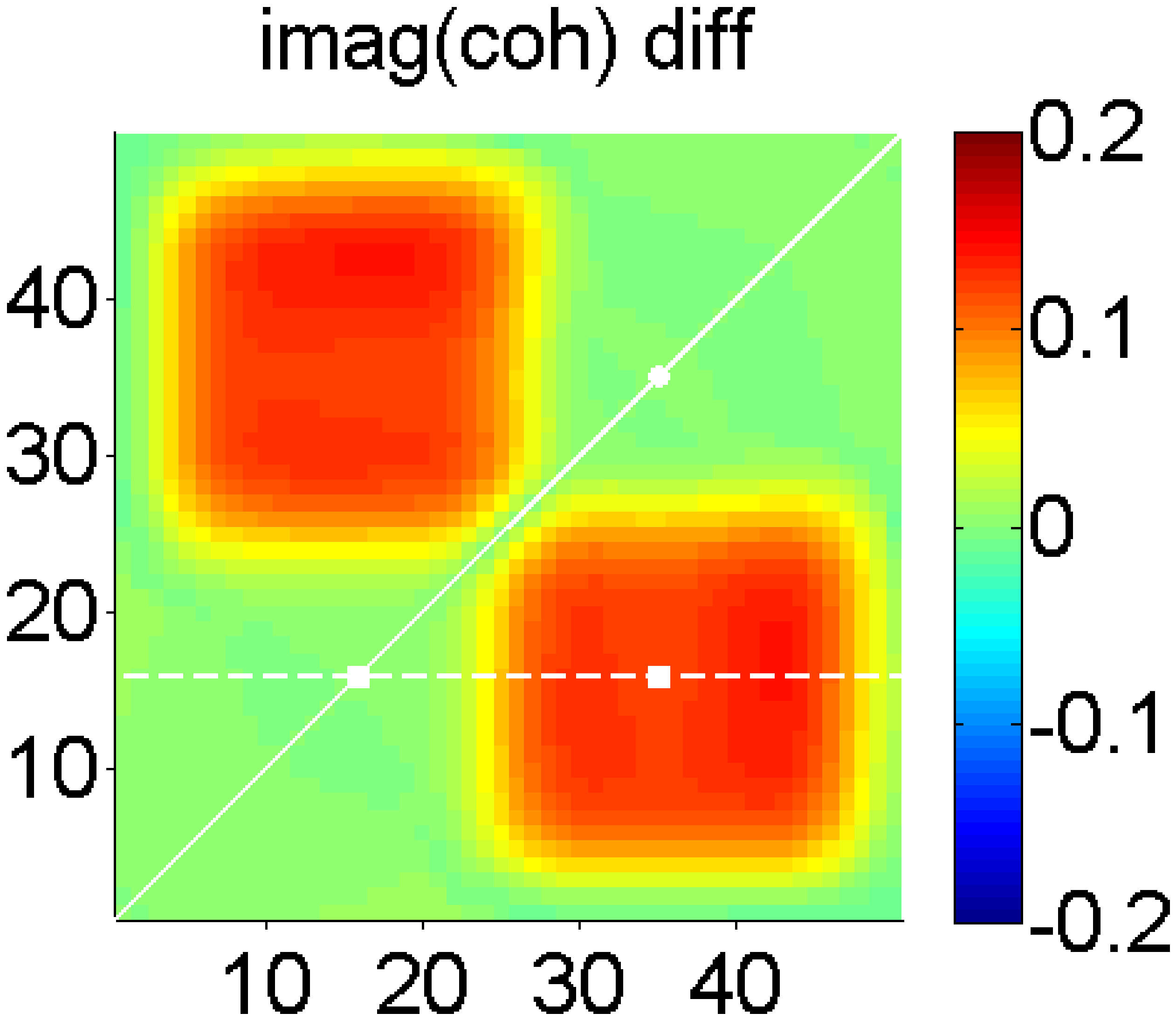

PRO version Are you a developer? Try out the HTML to PDF API pdfcrowd.com

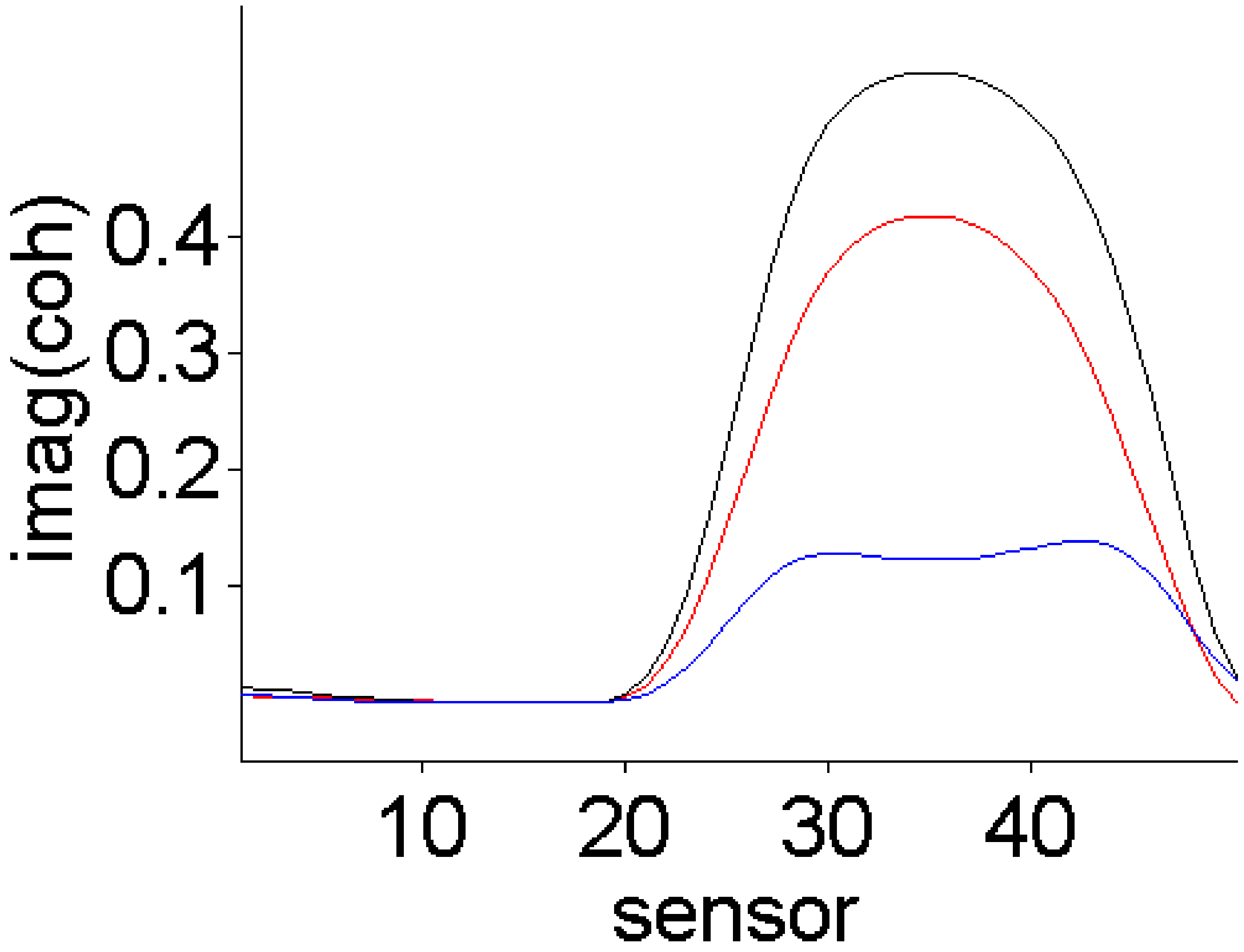

Published with MATLAB® R2013a

PRO version Are you a developer? Try out the HTML to PDF API pdfcrowd.com
